# Engineering *Saccharomyces cerevisiae* for the *de novo* Production of Halogenated Tryptophan and Tryptamine Derivatives

**DOI:** 10.1002/open.202200266

**Published:** 2023-03-16

**Authors:** Nicholas Milne, Javier Sáez‐Sáez, Annette Munch Nielsen, Jane Dannow Dyekjær, Daniela Rago, Mette Kristensen, Tune Wulff, Irina Borodina

**Affiliations:** ^1^ The Novo Nordisk Foundation Center for Biosustainability Technical University of Denmark 2800 Kgs. Lyngby Denmark; ^2^ Octarine Bio ApS Lersø Parkallé 42, 1. Sal 2100 Copenhagen Denmark

**Keywords:** halogenases, indole, in vivo halogenation, metabolic engineering, *Saccharomyces cerevisiae*

## Abstract

The indole scaffold is a recurring structure in multiple bioactive heterocycles and natural products. Substituted indoles like the amino acid tryptophan serve as a precursor for a wide range of natural products with pharmaceutical or agrochemical applications. Inspired by the versatility of these compounds, medicinal chemists have for decades exploited indole as a core structure in the drug discovery process. With the aim of tuning the properties of lead drug candidates, regioselective halogenation of the indole scaffold is a common strategy. However, chemical halogenation is generally expensive, has a poor atom economy, lacks regioselectivity, and generates hazardous waste streams. As an alternative, in this work we engineer the industrial workhorse *Saccharomyces cerevisiae* for the *de novo* production of halogenated tryptophan and tryptamine derivatives. Functional expression of bacterial tryptophan halogenases together with a partner flavin reductase and a tryptophan decarboxylase resulted in the production of halogenated tryptophan and tryptamine with chlorine or bromine. Furthermore, by combining tryptophan halogenases, production of di‐halogenated molecules was also achieved. Overall, this works paves the road for the production of new‐to‐nature halogenated natural products in yeast.

## Graphical abstract

***Saccharomyces cerevisiae*** **strains** expressing several tryptophan halogenases, a flavin reductase, and a tryptophan decarboxylase are used for the in vivo biosynthesis of halogenated tryptophan and tryptamine derivatives. The products can serve as halogenated building blocks for the synthesis of pharmaceuticals or new‐to‐nature halogenated natural products.

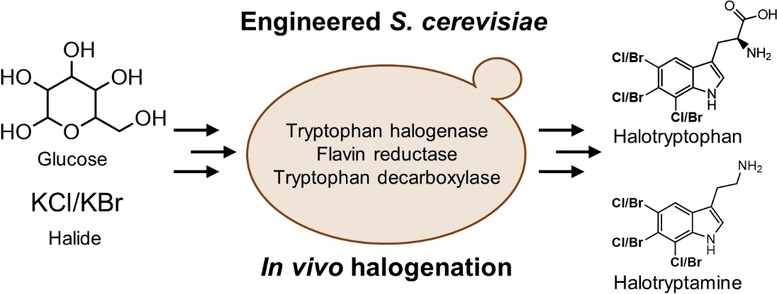

## Introduction

**Figure 1:**
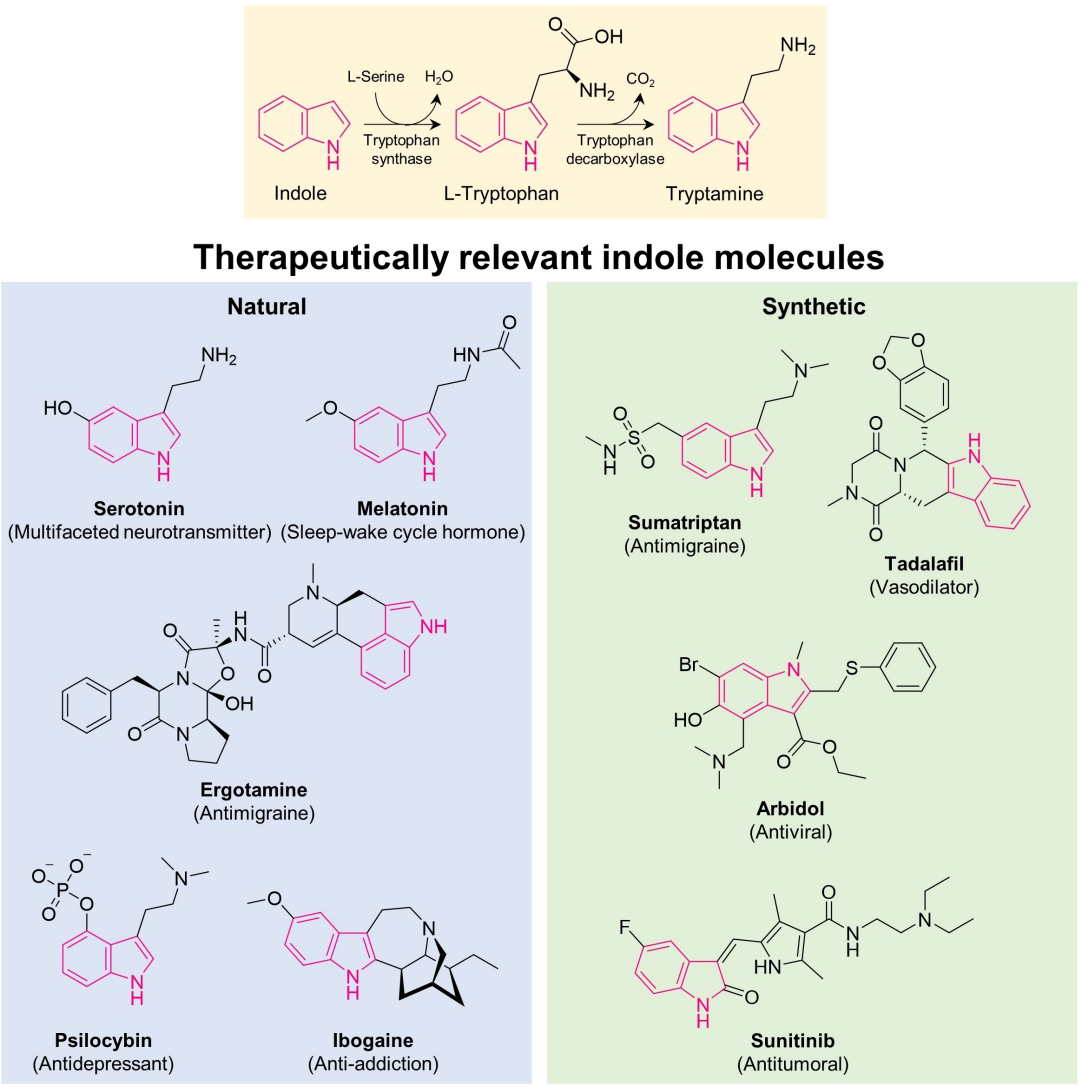
Schematic representation of tryptophan and tryptamine biosynthesis and the prevalence of the indole scaffold in naturally occurring and synthetic chemical compounds. The indole scaffold in different chemical structures is shown in pink. The therapeutic use of each of the compounds is described in brackets.

Heterocycles are fundamental structural moieties present in more than half of the biologically active compounds found in nature.^[1]^ Indole scaffolds in particular are a prominent example of a prevalent natural heterocycle with multi‐disciplinary applications as pharmaceutical drugs or agrochemicals.^[2]^ Indole is chemically constituted by a six‐membered benzene ring fused to a five‐membered pyrrole ring, and is produced by plants, fungi, and bacteria, which makes it ubiquitously present in nature.^[3]^ The prevalence of the indole scaffold in nature can be owed to the essential amino acid tryptophan, which is biosynthesized by the condensation of indole and serine.^[4]^ Tryptophan in turn serves as a precursor for multiple natural bioactive compounds, like animal hormones (substituted tryptamines including serotonin and melatonin), plant hormones (indole‐3‐acetic acid, indole‐3‐propionic acid), plant‐derived marketed pharmaceuticals (vincristine, vinblastine), and psychedelics (psilocybin, ibogaine) (Figure 1).^[5]^ The versatility of the indole core makes it stand out as a so‐called “privileged structure”, a molecular scaffold that by the modification of functional groups can be converted into a variety of ligands with selective binding capacities against multiple biological targets.^[6]^ Indeed, medicinal chemists have for decades exploited indole as a core structure for drug discovery, resulting in multiple synthetic drugs used for the treatment of various disorders, including cancer, migraine, depression and hypertension (Figure 1).[^3a^, ^7^]

In such drug discovery processes, halogenation is commonly used as a means to improve the properties of a lead drug candidate,^[8]^ and consequently, the presence of halogens is frequent in drugs, with approximately 25 % of all commercialized pharmaceuticals containing a halogen atom in their structure.^[9]^ Halogenation of drug candidates can increase membrane permeability,^[10]^ enhance blood‐brain barrier transport,^[11]^ and strengthen the binding interactions between a ligand and its target, thereby increasing the ligand potency.[^9^, ^12^] The halogenation of the indole ring in particular has been proven to tune some properties of multiple cancer drug leads isolated from marine environments.^[13]^ Furthermore, halogenated indoles serve as versatile precursors for the synthesis of more complex structures, as they facilitate cross‐coupling reactions and other synthetic transformations.^[14]^

Although multiple methods for the halogenation of indole heterocycles exist, these often involve the use of toxic reagents like transition metals,^[15]^ generate significant amounts of by‐products,^[16]^ and the regioselectivity is mostly achieved only at the 2‐ and 3‐positions on the indole ring.^[17]^ The addition of directing groups to the indole ring can also facilitate halogenation at the benzene ring, however, this strategy is associated with additional synthetic steps and reduced atom economy.[^17^, ^18^] Therefore, catalyst‐controlled methods employing enzymes that perform site‐selective halogenations is emerging as a promising approach in synthetic chemistry, as they provide a means to overcome the electronic preference for substitution at the 2‐ and 3‐positions.^[19]^ Among multiple classes of halogenating enzymes found in nature, flavin‐dependent halogenases (FDHs) stand out for their marked substrate specificity and regioselectivity.^[20]^ In addition to oxygen and halide ions, FDHs require input of FADH_2_ provided by a partner flavin‐reductase that performs NADH‐driven reduction of FAD to FADH_2_.^[20a]^ Tryptophan halogenases in particular are well‐studied FDHs able to halogenate benzo positions of the indole ring in tryptophan, with multiple studies showing the use of these enzymes to halogenate tryptophan with chlorine or bromine in the 5‐, 6‐ and 7‐positions in vitro.^[21]^ However, an in vitro large‐scale process is hindered by the low activity and stability of tryptophan halogenases as well as a need to add the expensive cofactors FAD, NADH, a partner reductase, and a cofactor‐regenerating method based on additional enzymes.^[22]^ The high cost of adding any of these cofactors would likely make the process economically infeasible. Alternatively, in vivo production strategies using recombinant microorganisms circumvent some of these limitations and could be a preferred system. The bacterium *Corynebacterium glutamicum* has been engineered for the expression of tryptophan halogenases, tryptophan decarboxylase, and trytophanase, resulting in production of significant levels of 7‐Br/Cl‐tryptophan, 7‐Br/Cl‐tryptamine, and 7‐Br/Cl‐indole.^[23]^ More recently, *Escherichia coli* has been used as chassis for the production of the fabric dye Tyrian purple and other pigments, composed of a halogenated indole skeleton.^[24]^ While these studies show the potential of prokaryotic cell factories for the production of halogenated indoles, complex derivatives thereof with interesting properties could be challenging to produce, as these often entail the functional expression of cytochrome P450 enzymes, which remains a challenge in bacteria.^[25]^ Conversely, the baker's yeast *Saccharomyces cerevisiae* is a preferred production chassis in the context of natural products, not only due to its eukaryotic cell nature that facilitates the expression of eukaryotic enzymes, but also supported by its scalability, its long industrial use as production host and its minimal production of secondary metabolites.^[26]^

The current work represents a proof‐of‐concept for the *de novo* production of halogenated indole derivatives in *S. cerevisiae*, specifically halogenated tryptophan and tryptamine.

## Results and Discussion

### Expression of tryptophan halogenases in *S. cerevisiae* leads to the production of halogenated tryptophan

**Figure 2:**
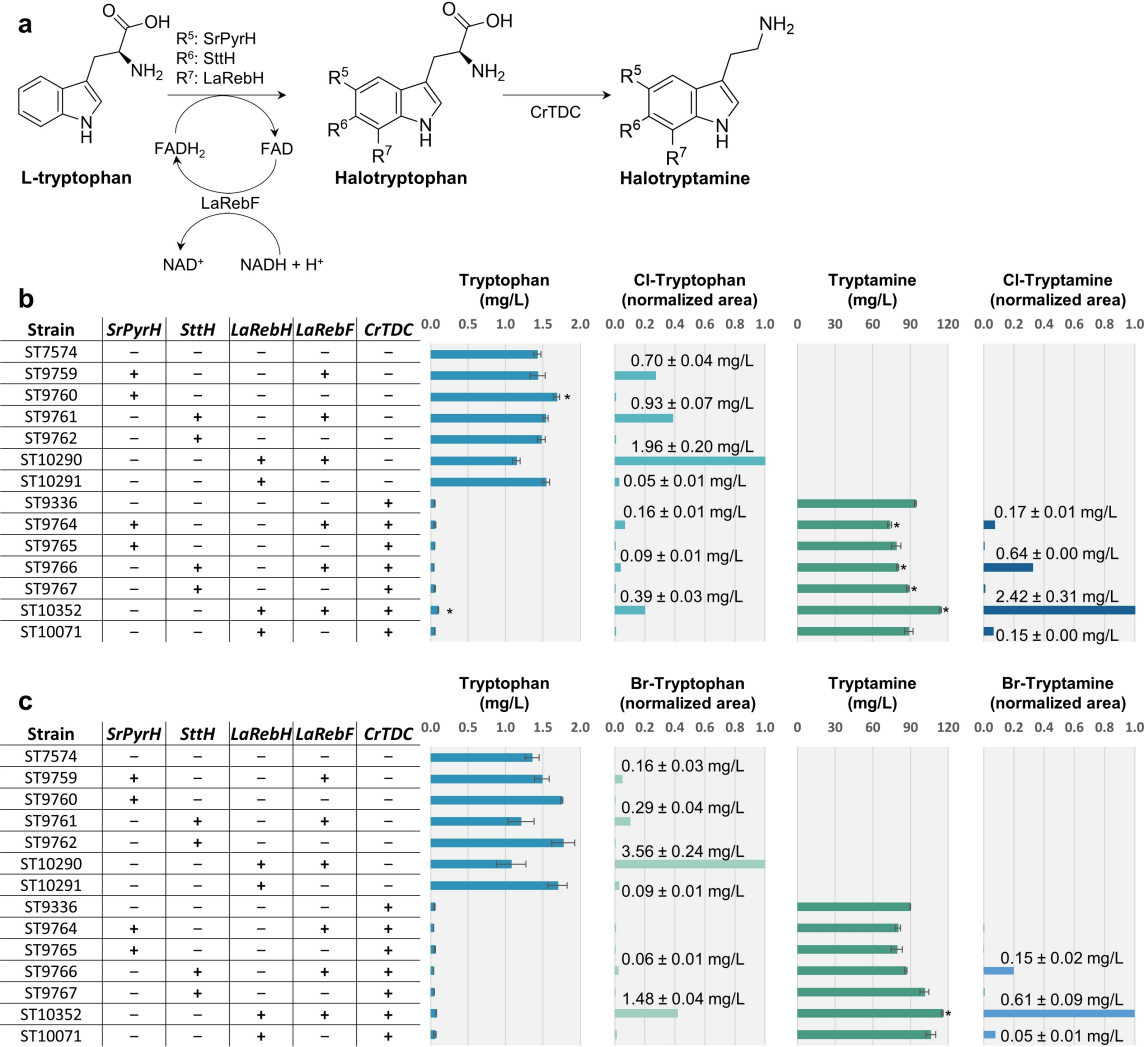
Production of halogenated tryptophan and tryptamine in recombinant yeast strains. a) Overview of heterologous biosynthesis of halogenated tryptophan and tryptamine in *S. cerevisiae* from the native precursor l‐tryptophan. R^n^ groups in halotryptophan and halotryptamine represent a Cl or Br atom in the positions indicated by *n*. Production of halogenated compounds in synthetic mineral medium supplemented with b) 25 mm KCl or c) 25 mm KBr after 72 h cultivation. “+” and “−” symbols indicate the presence or absence of the corresponding genetic modification, respectively. Cultivation broths of strains lacking CrTDC were subjected to the intracellular extraction protocol. Cultivation broths of strains expressing CrTDC were centrifuged and the supernatants were used for the analysis. Titers of halogenated products are reported as normalized peak areas, meaning that areas matching the retention time, expected m/z, and fragmentation pattern of the metabolite of interest have been normalized with respect to the highest‐producing strain of that metabolite. Titers of halogenated products not reported in mg/L were below the lowest calibration point (<0.04 mg/L). Error bars/values represent the standard deviation from two biological replicates. Asterisk (*) indicates a p‐value <0.05 (two‐tailed, unequal variance) between the strain and the control (ST7574 for strains lacking CrTDC, ST9336 for strains expressing CrTDC). Data available in the Supporting xlsx File 1.

In nature, numerous bioactive compounds with halogenated indoles in their chemical structures have been discovered, leading to the characterization of several tryptophan FDHs able to act on the 5‐, 6‐, and 7‐positions of the indole moiety of tryptophan.^[21]^ Among several described tryptophan halogenases, SrPyrH (5‐halogenase) from *Streptomyces rugosporus*, SttH (6‐halogenase) from *Streptomyces toxytricini*, and LaRebH (7‐halogenase) from *Lechevalieria aerocolonigenes* have been demonstrated to be functional in bacteria and plants when co‐transformed with a partner flavin reductase, like LaRebF from *Lechevalieria aerocolonigenes*.[^23b^, ^27^] Additionally, in plants, the tryptophan decarboxylase CrTDC from *Catharanthus roseus* accepts halogenated tryptophan as a substrate, leading to the production of halogenated tryptamine.[^27^, ^28^] Inspired by these works, we hypothesized that SttH, SrPyrH and LaRebH might also catalyze halogenation of tryptophan when co‐expressed with LaRebF in *S. cerevisiae* and that introduction of the genes into a strain expressing CrTDC would lead to production of halogenated tryptamine (Figure 2a).

To attempt production of halogenated tryptophan, integrations of SrPyrH, SttH and LaRebH into *S. cerevisiae* strain ST7574 (CEN.PK113‐7D+Cas9) were carried out with and without co‐integration of the partner flavin reductase LaRebF. The same integrations were additionally performed in strain ST9336 expressing CrTDC in order to attempt production of halogenated tryptamine.[^26b^, ^29^] A proteomic analysis confirmed high expression levels of the heterologous genes in multiple engineered strains (Figure S1, Supporting Information, and Supporting xlsx File 1). Transformants were cultivated in synthetic mineral media supplemented with 25 mm KCl or 25 mm KBr (Figure 2b‐c). We also performed computer‐aided docking simulations to investigate the binding interactions between tryptophan, the different positional isomers of halotryptophan, and the tryptophan halogenases. Likewise, we evaluated the binding of halotryptophan and halotryptamine to CrTDC (Figures S2–S3 and Tables S1–S2).

Chlorotryptophan or bromotryptophan were present in all strains expressing a tryptophan halogenase and cultivated in a medium supplemented with the corresponding halide (Figures S4–S9), even in the absence of LaRebF. However, the levels of halogenated tryptophan were up to 100‐fold higher when LaRebF was expressed, clearly demonstrating that FADH_2_ supply was the limiting factor for the halogenation. Additionally, the intracellular tryptophan titers in the strains expressing halogenases did not demonstrate significant variations compared to the control strain, except for strain ST10290 harboring the 7‐halogenase LaRebH and LaRebF. This indicates that, in general, only a limited proportion of tryptophan was halogenated, with a notable exception being the activity of LaRebH. Docking simulations suggested that indeed substrate is more favorably bound to LaRebH, potentially resulting in a higher activity (Figure S2, Table S1). Halogenation of tryptophan by SrPyrH resulted in the lowest titers, which is consistent with previously reported in vitro data on low activity and particularly low affinity for bromine. Specifically, previous studies have shown that SrPyrH activity towards bromination is 75 % lower than its activity towards chlorination.^[21b]^ More interestingly, halogenated products from the kynurenine pathway, such as halogenated L‐kynurenine, kynurenic acid, and xanthurenic acid were identified when expressing either of the halogenases, indicating that the native enzymes in yeast can incorporate halogenated tryptophan into the kynurenine pathway (Figures S10–S17).

Strains expressing a tryptophan halogenase together with CrTDC and cultivated with the corresponding halide resulted in the production of chloro‐ or bromotryptamine (Figures S18–S23). The analytical standards allowed the determination of the regioselectivity and the quantification of products demonstrating an overall low catalytic activity of the enzymes. The highest titers obtained for halogenated tryptophan and tryptamine were 3.56±0.24 mg/L 7‐bromotryptophan and 2.42±0.31 mg/L 7‐chlorotryptamine. In addition, the strains producing brominated compounds were also able to perform chlorination, even though chloride was only present in trace amounts in the medium (0.15 mm; Figure S24), indicating that chlorination could be favored, which is in line with the general consensus regarding flavin‐dependent tryptophan halogenases.^[30]^

### Tryptophan halogenases accept tryptamine as substrate

**Figure 3:**
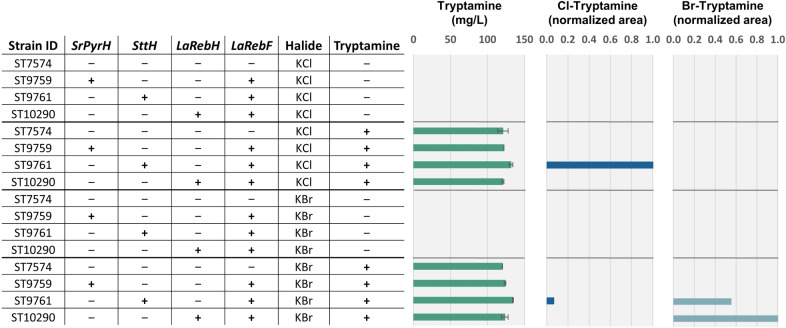
Direct halogenation of tryptamine in engineered *S. cerevisiae* strains expressing tryptophan halogenases. Cultivations were performed in synthetic mineral medium for 72 h. “+” and “−” symbols indicate the presence or absence of the corresponding genetic modification, respectively. The “halide” column shows the halide compound added in the medium at a concentration of 25 mm, while the “tryptamine” column indicates the supplementation (“+”) or absence (“−”) of 1 mm tryptamine. Cultivation broths were centrifuged and the supernatants were used for the analysis. Titers of halogenated products are reported as normalized peak areas, meaning that areas matching the retention time, expected m/z, and fragmentation pattern of the metabolite of interest have been normalized with respect to the highest‐producing strain of that metabolite. Titers of all halogenated products were below the lowest calibration point (<0.04 mg/L). Error bars represent the standard deviation from two biological replicates. Data available in the Supporting xlsx File 1.

While in plants the sequence of reactions leading to halogenated tryptamine is assumed to begin with the halogenation of tryptophan, which is subsequently decarboxylated by CrTDC yielding halotryptamine,^[27]^ some tryptophan halogenases like LaRebH have been shown to present some substrate promiscuity, being able to act directly on tryptamine.^[31]^ In order to assess whether the different halogenases we tested could accept tryptamine as substrate in *S. cerevisaie*, strains expressing individual tryptophan halogenases together with LaRebF were cultivated in synthetic mineral medium supplemented with the corresponding halide and fed with 1 mm tryptamine (Figure 3).

Interestingly, SttH was able to halogenate tryptamine with both chlorine and bromine (Figures S25–S26), while LaRebH could only perform tryptamine bromination (Figure S27). The regioselectivity of the halogenation seemed to be maintained in all reactions (Figures S25–S27). This data suggests that the tryptamine halogenations in strains harboring CrTDC and SttH or LaRebH reported in Figure 2 were likely consequence of both the decarboxylation of halogenated tryptophan and the direct halogenation of tryptamine. In either case, both enzymatic reactions involve a non‐native substrate, which has been shown to result in a poor catalytic activity. For example, the catalytic efficiency of LaRebH is significantly lower when using tryptamine as a substrate, with a 59‐fold reduction compared to tryptophan.[^27b^, ^31b^] This is reflected in the low titers of halotryptamine obtained by direct halogenation, which were found to be below 0.04 mg/L. Finally, halogenated tryptamine was not observed for strains expressing SrPyrH indicating that this enzyme is specific for tryptophan.

### Co‐expression of tryptophan halogenases results in the production of di‐halogenated tryptophan

The expression of individual tryptophan halogenases and tryptophan decarboxylase resulted in the production of halotryptophan and halotryptamine. In order to investigate whether production of multi‐halogenated tryptophan or tryptamine would be possible in *S. cerevisiae*, co‐integrations of *SrPyrH*, *SttH*, *LaRebH* and *LaRebF* were carried out in strains ST7574 and ST9336, resulting in strains expressing several tryptophan halogenases with or without CrTDC.

**Figure 4:**
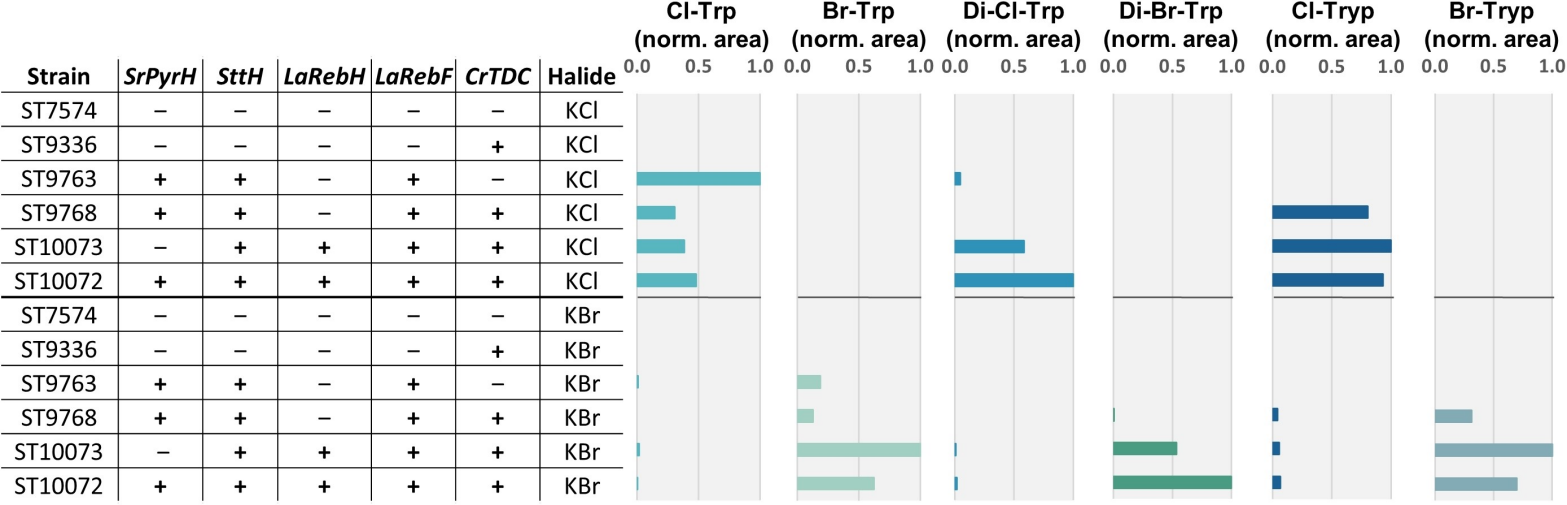
Production of dihalogenated tryptophan in engineered *S. cerevisiae* strains. Cultivations were performed in synthetic mineral medium for 72 h. “+” and “−” symbols indicate the presence or absence of the corresponding genetic modification, respectively. The “halide” column shows the halide compound added in the medium at a concentration of 25 mm. Cultivation broths of strains lacking *CrTDC* were subjected to the intracellular extraction protocol. Cultivation broths of strains expressing *CrTDC* were centrifuged and the supernatants were used for the analysis. Titers of halogenated products are reported as normalized peak areas, meaning that areas matching the retention time (except for dihalotryptophan), expected m/z, and fragmentation pattern of the metabolite of interest have been normalized with respect to the highest‐producing strain of that metabolite. Trp: tryptophan; Tryp: tryptamine. Data available in the Supporting xlsx File 1.

In medium containing the corresponding halide, strains expressing two or three tryptophan halogenases and LaRebF produced a metabolite with mass and fragmentation pattern matching dichloro‐ or dibromotryptophan, suggesting that two tryptophan halogenases worked in conjunction to perform di‐halogenation of tryptophan (Figures 4, S28–S31). However, no metabolite matching trihalotryptophan was detected in strains harboring three halogenases. Furthermore, when CrTDC was co‐expressed, dihalotryptamine was not produced, indicating both that CrTDC cannot accept dihalotryptophan as substrate and that halotryptamine cannot be halogenated a second time by the remaining halogenases. Interestingly, strain ST9763 expressing two halogenases produced dichlorotryptophan, while ST9678 expressing the same halogenases in addition to CrTDC produced chlorotryptamine but not dichlorotryptophan, indicating a higher catalytic activity of CrTDC towards the shared substrate, namely chlorotryptophan. When these two strains were cultivated in KBr containing medium, dibromotryptophan was produced by strain ST9768, but surprisingly not by strain ST9763.

## Discussion

In this study, conceptual *de novo* production of a diverse range of halogenated tryptophan and tryptamine derivatives was achieved in the baker's yeast *S. cerevisiae* by the functional expression of bacterial tryptophan halogenases and a partner flavin reductase providing FADH_2_. While the expression of the flavin reductase LaRebF was demonstrated to be fundamental for the production of halogenated metabolites, trace amounts were also detected even in the absence of LaRebF (Figure 2). The cofactor FADH_2_ provided by LaRebF is natively produced in the citric acid cycle in *S. cerevisiae*, but as this takes place in the mitochondria and the halogenases are expressed in the cytosol, it is unlikely that the FADH_2_ generated this way could be responsible for the observed halogenations.^[32]^ Other studies have similarly reported results indicating the presence of FADH_2_ in the cytosol of *S. cerevisiae* without identifying the source.^[33]^

While the co‐expression of tryptophan halogenases along with LaRebF and CrTDC resulted in the production of halogenated compounds, the low titers obtained and the concomitant accumulation of tryptophan and tryptamine suggested limited performance in *S. cerevisiae* (Figure 2). Indeed, as previously described in literature, the decarboxylation of halogenated tryptophan by CrTDC has been reported to be up to 30‐fold lower in terms of catalytic efficiency compared to the native substrate tryptophan in vitro.^[27b]^ Likewise, the direct halogenation of tryptamine has a turnover number 48‐fold lower than tryptophan halogenation for LaRebH.^[31b]^ Therefore, the use of enzyme variants with increased activity or shifted substrate specificity could help relieving sub‐optimal enzymatic steps.^[34]^ In this regard, the noncanonical aromatic amino acid decarboxylase PcncAAAD from the hallucinogenic psilocybin mushroom *Psilocybe cubensis* has been demonstrated to display higher chlorotryptamine production than CrTDC.^[35]^

Similarly, the flavin reductase LaRebF has been systematically used in most of the works involving tryptophan halogenases, but many other flavin reductases have been characterized and could show better results in the reduction of FAD in yeast.^[36]^ The endogenous cytosolic supply of FAD in *S. cerevisiae* has also been demonstrated to be limiting the production of compounds whose biosynthesis requires this cofactor, which can be alleviated by overexpressing native genes involved in its biosynthesis.^[37]^ Another factor that could be affecting the halogenation reaction is the intracellular availability of Cl^−^ and Br^−^, dependent on the transport kinetics for these anions.^[38]^ The uptake kinetics of these anions into the cells has been shown to be affected by pH and temperature.^[39]^ Thus, adjustments to these factors, as well as the overexpression of uptake transporters or the increase of halide concentrations in the medium, could improve their intracellular availability. In addition, engineering in the shikimate pathway has been proven to increase the titers of tryptophan‐derived products,[^29^, ^40^] which combined with the expression of engineered halogenase variants and a better cofactor supply could improve the production of halogenated compounds.

Although low titers were obtained, this study demonstrates the feasibility of *S. cerevisiae* as a production chassis for halogenated indoles. Some of the products obtained like 6‐bromotryptamine could have direct applications in the pharmaceutical industry. The derivative 6‐bromotryptamine A has been demonstrated to inhibit the activity of acetylcholinesterase in vitro and prevent scopolamine‐induced short‐term cognitive impairments in mice, thereby alleviating two main pathological events in the progress of Alzheimer's disease.^[41]^ Therefore, the biosynthesis of 6‐bromotryptamine in *S. cerevisiae* would offer an alternative to the current chemical synthesis methods.^[41]^ Similarly, 5‐bromotryptamine could be converted to 5‐bromo‐DMT, a metabolite found in marine sponges that has sedative properties.^[42]^

The identification of halogenated molecules within the kynurenine pathway presents opportunities for the manipulation of yeast to produce these compounds. This is of particular interest as imbalances in the kynurenine pathway have been associated with various psychiatric disorders, including depression and schizophrenia.^[43]^ 4‐Chloro‐kynurenine, for example, has been found to have potential as a therapeutic prodrug for major depressive disorder.^[44]^ Additionally, other halogenated kynurenine derivatives such as bromo‐kynurenine play a role in the striking green biofluorescence observed in certain species of shark.^[45]^

More importantly, tryptamine serves as a precursor for more than 3,000 secondary metabolites including monoterpenoid indole alkaloids (MIAs) like vinblastine or vincristine, important chemotherapy agents.^[46]^ By feeding with halogenated indoles or expressing tryptophan halogenases in the medicinal plant *C. roseus*, production of multiple halogenated MIAs has been previously demonstrated in vivo, as most of the biosynthetic enzymes accept halogenated intermediates, while in some steps an engineered variant is required.[^27b^, ^47^] In addition, functional expression of the enzymes leading to the MIA common intermediate strictosidine has been successfully achieved in yeast,^[26b]^ which in combination with the results of the present work proves that *S. cerevisiae* could be a promising host for the production of new‐to‐nature halogenated natural products useful in drug discovery processes.

## Conclusions

In this work, the successful production of various halogenated tryptophan and tryptamine derivatives was achieved in the industrial workhorse *Saccharomyces cerevisiae*. Functional expression of bacterial tryptophan halogenases together with a partner flavin reductase and a tryptophan decarboxylase resulted in the production of halogenated tryptophan and tryptamine with chlorine or bromine. Additionally, we showed that some tryptophan halogenases present some substrate promiscuity and can halogenate tryptamine directly and that the co‐expression of two tryptophan halogenases results in the production of dihalotryptophan. Overall, this work demonstrates the suitability of *S. cerevisiae* for the production of halogenated natural products.

## Experimental Section

### Strains and media

Haploid *Saccharomyces cerevisiae* strain CEN.PK113‐7D (MATa *URA3 HIS3 LEU2 TRP1 MAL2‐8c SUC2*) was used as parental strain in this study.^[48]^ *S. cerevisiae* cultures were grown in liquid Yeast Peptone Dextrose (YPD). The media were supplemented with 200 mg/L G418 for selection of the Cas9‐plasmid and with 100 mg/L nourseothricin for selection of gRNA plasmids when required. Synthetic mineral medium for cultivation was prepared with 7.5 g/L (NH_4_)_2_SO_4_, 14.4 g/L KH_2_PO_4_, 0.5 g/L MgSO_4_ ⋅ 7H_2_O, 1 mL/L vitamins, 2 mL/L trace elements and with 20 g/L glucose.^[49]^ The pH was adjusted to 6.0 with NaOH. In the halogenation experiments, the media were supplemented with 25 mm KCl, 25 mm KBr or 1 mm tryptamine as required.

*Escherichia coli DH5α* was used for all plasmid cloning and propagation. *E. coli* strains were grown in Lysogeny Broth (LB) media supplemented with 100 mg/L ampicillin. Agar plates for both *S. cerevisiae* and *E. coli* cultivations were prepared as described above with addition of 20 g/L agar. Frozen stocks of *S. cerevisiae* and *E. coli* were prepared by adding glycerol to a final concentration of 25 % (v/v) to overnight cultures and storing aliquots at −80 °C.

### Construction of plasmids and strains

Relevant heterologous genes were codon‐optimized for *S. cerevisiae* using the JCat online tool^[50]^ and ordered as synthetic DNA strings from GeneArt (Thermo Fisher Scientific) (Table S3). The EasyClone‐MarkerFree cloning system^[51]^ was used to construct integration plasmids. Integration fragments and gRNA plasmids for CRISPR/Cas9‐mediated integration were constructed as described by Jessop Fabre et al.^[51]^ BioBricks were amplified using USER‐compatible primers and Phusion U Hot Start DNA Polymerase (Thermo Fisher Scientific). USER reactions were carried out according to New England Biolabs standard protocol. The USER reactions were then transformed into competent *E. coli DH5α* cells by heat shock for plasmid assembly and propagation. Plasmids were purified from *E. coli* cultures using NucleoSpin plasmid miniprep kit (Macherey Nagel) and correct plasmid assembly was verified by Sanger sequencing (Eurofins Scientific). Integration fragments were linearized with FastDigest NotI restriction enzyme (Thermo Fisher Scientific). Integration fragments and gRNA plasmids when relevant were transformed into *S. cerevisiae* strains expressing Cas9 using the LiAc/SS carrier DNA/PEG method.^[52]^ When involving nourseothricin selection, transformations were recovered in YPD+G418 media at 30 °C with 250 rpm shaking before plating. Transformations were plated on YPD supplemented with G418 and nourseothricin. Integrations of the vectors into the correct sites on the genome were verified by colony PCR using Red Taq (VWR Life Science) DNA polymerase. To prepare transformed strains for subsequent CRISPR/Cas9 mediated integrations, gRNAs were removed by re‐streaking on YPD plates supplemented with G418 to maintain the selection for Cas9‐vector and removal of the gRNAs was verified by replica plating. A list of all strains, plasmids, BioBricks and primers used and constructed in this study is available in the Supporting Information (Tables S4–S7).

### Cultivation and extraction

Cultivations of *S. cerevisiae* strains were performed in synthetic mineral medium. Single colonies were inoculated into 400 μL media in 96‐deep well plates with air‐penetrable lids (Enzyscreen) and incubated for 48 h at 30 °C and 300 rpm. From these pre‐cultures, 10 μL were transferred to 490 μL media supplemented with relevant substrates when required and incubated for 72 h in 96‐deep well plates with air‐penetrable lids at 30 °C and 300 rpm. Cultivations were performed in biological duplicates when metabolites were to be quantified.

For extraction of intracellular products, the cultivation broths were subjected to cell lysis, which was carried out by adding a small aliquot of acid‐washed glass beads (212−300 μm, Sigma‐Aldrich) and running the samples for two cycles of 20 sec at 5,500 rpm on a Precellys 24 Tissue Homogenizer (Bertin Instruments). The lysed cell broths were centrifuged at 17,000 g for 5 min and the supernatants were analyzed by LC‐MS/MS. For the quantification of tryptamine and halogenated derivatives, the cultivation broths were centrifuged at 17,000 g for 5 min and the supernatants were analyzed by LC‐MS/MS.

For the proteomics analysis, single colonies were inoculated in 2 mL of synthetic mineral medium and grown overnight in 13 mL pre‐culture tubes. The next day, 24‐deep well plates with air‐penetrable lids (Enzyscreen) containing 2 mL of synthetic mineral medium were inoculated to an OD of ∼0.1. Cultures were grown for 20 h at 30 °C and 300 rpm. 400 μL of broth, corresponding to approximately 2×10^7^ cells, was centrifuged at 3,000 g for 10 min, the supernatant was discarded and cell pellets were frozen at −80 °C until analysis.

### Analytical methods

The LC‐MS/MS analysis of the samples was performed on a Vanquish Duo UHPLC binary system (Thermo Fisher Scientific) coupled to IDX‐Orbitrap Mass Spectrometer (Thermo Fisher Scientific, USA). The chromatographic separation was achieved using a Waters ACQUITY BEH C18 (10 cm×2.1 mm, 1.7 μm) equipped with an ACQUITY BEH C18 guard column kept at 40 °C and using a flow rate of 0.35 mL/min as previously described.^[53]^ A binary mobile phase system composed of 0.1 % formic acid in MilliQ water (solvent A) and 0.1 % formic acid in acetonitrile (solvent B) was employed. The gradient program began with 2 % B for 0.8 min, followed by a linear increase to 5 % B at 3.3 min. Subsequently, solvent B was increased until reaching 100 % at 10 min, held for 1 min, and then returned to the initial conditions. The column was allowed to re‐equilibrate for 2.7 min before the next injection. The injection volume was 1 μL. The MS measurement was carried out in positive‐heated electrospray ionization (HESI) mode with a voltage of 3500 V, the vaporizer temperature was set at 350 °C, the ion transfer tube temperature at 325 °C, and the sheath gas at 50 (a.u.). Acquisition was performed in full MS/MS spectra (Data dependent Acquisition‐driven MS/MS) in the mass range of 70–1000 Da. The DDA acquisition settings were the following: automatic gain control (AGC) target value set at 4e5 for the full MS and 5e4 for the MS/MS spectral acquisition, the mass resolution was set to 120,000 for full scan MS and 60,000 for MS/MS events. Precursor ions were fragmented by stepped High‐energy collision dissociation (HCD) using collision energies of 20, 40, and 60 eV. A lock mass of an internal calibrant was acquired throughout each injection to provide real‐time adjustment of the instrument‘s m/z calibration. Peaks were integrated using QuanBrowser Thermo Xcalibur 4.2 (Thermo Fisher Scientific).

Analytical standards were purchased from Sigma‐Aldrich (l‐tryptophan, tryptamine), Carbosynth (5‐chlorotryptophan, 6‐chlorotryptophan, 5‐bromotryptophan, 7‐bromotryptophan, 5‐chlorotryptamine hydrochloride, 6‐chlorotryptamine, 7‐chlorotryptamine hydrochloride, 6‐bromotryptamine hydrochloride, 7‐bromotryptamine hydrochloride), Toronto Research Chemicals (7‐chlorotrpytophan), BLDpharm (6‐bromotryptophan), and VWR (5‐bromotryptamine hydrochloride).

The calibration curves were generated using five concentration points per compound, over a concentration range of 0.04 to 6.25 mg/L, with the exception of tryptamine, which ranged from 1.72 to 160 mg/L. Each calibration point was determined through a single injection, which were randomly interspersed throughout the analytical run. The calibration curve data was modeled using ordinary least squares regression using the statsmodels 0.13.5 package in Python (Supporting xlsx File 1).The statistical significance of differences between measurements from biological replicate samples were calculated by using a two‐tailed t‐test assuming unequal variances.

### Proteomic analysis

The frozen cell pellets derived from the cultivations were thawed on ice. Oxide beads (Glen Mills, NJ, USA) and 100 μL of 95 °C Guanidinium HCl (6 m Guanidinium hydrochloride (GuHCl), 5 mm tris(2‐carboxyethyl)phosphine (TCEP), 10 mm chloroacetamide (CAA), 100 mm Tris−HCl pH 8.5) was added to all samples. Full disruption of the cells was achieved in a Mixer Mill (MM 400 Retsch, Haan, Germany) set at 25 Hz for 5 min at room temperature, followed by 10 min in a thermo mixer at 95 °C at 600 rpm. Cell debris was removed by centrifugation at 5,000 g for 10 min. A total of 100 μg protein were used for overnight tryptic digestion (constant shaking, 400 rpm, for 8 h), after which 10 μL of 10 % TFA was added. Samples were then ready for de‐salting using a uSOLA C18 plate (Thermo Fisher Scientific).

Desalted samples were injected into Orbitrap Exploris 480 mass spectrometer (Thermo Fisher Scientific) using a CapLC system (Thermo Fisher Scientific). First, samples were captured at a flow of 10 μL/min on a precolumn (μ‐precolumn C18 PepMap 100, 5 μm, 100 Å) and then at a flow of 1.2 μL/min the peptides were separated on a 15 cm C18 EASY‐Spray column (PepMap RSLC C18 2 μm, 100 Å, 150 μm×15 cm). The applied gradient went from 4 % acetonitrile in water (buffered with 0.1 % formic acid) to 76 % over a total of 60 min. While spraying the samples into the mass spectrometer the instrument operated in data dependent mode using the following settings: MS‐level scans were performed with Orbitrap resolution set to 60,000; AGC Target 3.0e6; maximum injection time 50 ms; intensity threshold 5.0e3; dynamic exclusion 25 sec. Data dependent MS2 selection was performed in Top 20 Speed mode with HCD collision energy set to 28 % (AGC target 1.0e4, maximum injection time 22 ms, Isolation window 1.2 m/z).

For analysis of the Thermo rawfiles, Proteome Discoverer 2.4 was used with the following settings: Fixed modifications: Carbamidomethyl (C) and Variable modifications: oxidation of methionine residues. First search mass tolerance 20 ppm and a MS/MS tolerance of 20 ppm. Trypsin as enzyme and allowing one missed cleavage. FDR was set at 0.1 %. The Match between runs window was set to 0.7 min. Quantification was only based on unique peptides and normalization between samples was based on total peptide amount. For the searches, a protein database consisting of the *Saccharomyces cerevisiae* reference proteome (UP000002311) and the heterologous proteins was used.

### Docking simulations

l‐tryptophan, 5‐Cl‐l‐tryptophan, 5‐Br‐l‐tryptophan, 6‐Cl‐l‐tryptophan, 6‐Br‐l‐tryptophan, 7‐Cl‐l‐tryptophan and 7‐Br‐l‐tryptophan were docked to crystal structures of SrPyrH (PDBID 2WET:B), SttH (PDB ID 5HY5:A) and LaRebH (PDB ID 2E4G:B). l‐tryptophan, tryptamine, 5‐Cl‐l‐tryptophan, 5‐Cl‐tryptamine, 5‐Br‐l‐tryptophan, 5‐Br‐tryptamine, 6‐Cl‐l‐tryptophan, 6‐Cl‐tryptamine, 6‐Br‐l‐tryptophan, 6‐Br‐tryptamine, 7‐Cl‐l‐tryptophan, 7‐Cl‐tryptamine, 7‐Br‐l‐tryptophan, and 7‐Br‐l‐tryptamine were docked to CrTDC (PDB ID 6EEW:AB).

The ligands were based on structures of l‐tryptophan (CHEBI_16828) and tryptamine (CHEBI_16765) by using MarvinSketch 19.19 (ChemAxon, http://www.chemaxon.com) to add Cl and Br at the 5‐, 6‐, and 7‐ positions, respectively, and calculate protonation state. The Optimize PyMOL plugin (The PyMOL Molecular Graphics System, Version 1.8 Schrödinger, LLC) was used to energy minimize the ligands, which finally were prepared for docking using AutoDockTools version 1.5.6 to merge non‐polar hydrogens, and add Gasteiger charges. The molecules were saved as rigid pdbqt files to retain their l‐configuration.^[54]^

Co‐crystallized ligands and water molecules were removed for the crystal structures of SrPyrH (PDBID 2WET:B), SttH (PDB ID 5HY5:A), LaRebH (PDB ID 2E4G:B) and CrTDC (PDB ID 6EEW:AB) and hydrogens were added with PyMOL. To mimic both chlorination and bromination, crystal structure Cl atoms were exchanged with Br. The positions of the active sites for SrPyrH, LaRebH and CrTDC were identified by calculating the center of mass of the bound l‐tryptophan ligand. No ligand was bound in the SttH crystal structure, so the active site position was identified as the center of mass of the active site residues Lys79, Glu363, His96 and Phe98. The LaRebH crystal structure does contain FAD and Cl/Br, so co‐factor and ions were added to the structure by superimposition with 2AR8. The structures were prepared for docking using AutoDockTools version 1.5.6 by merging non‐polar hydrogens and adding Gasteiger charges. The structures were saved as rigid pdbqt files to retain the position of the active site Lys75 and Glu354 (SrPyrH numbering) amino acids.

The molecules were docked to the structures using AutoDock Vina 1.1.2 using a box size of 10×10×10 centered in the active site.^[55]^ The protein‐ligand interactions were analyzed using binana 2.1.^[56]^

## Conflict of interest

N. M. and I. B. are inventors on patent application PCT/EP2020/075823. N .M. has a financial interest in Octarine Bio ApS.

1

## Supporting information

As a service to our authors and readers, this journal provides supporting information supplied by the authors. Such materials are peer reviewed and may be re‐organized for online delivery, but are not copy‐edited or typeset. Technical support issues arising from supporting information (other than missing files) should be addressed to the authors.

Supporting Information

## Data Availability

The data that support the findings of this study are available in the supplementary material of this article.
