# Supplementary material for: Engineering Saccharomyces cerevisiae for the de novo Production of Halogenated Tryptophan and Tryptamine Derivatives
Source: ChemistryOpen. 2023 Mar 16;12(4):e202200266. doi: 10.1002/open.202200266 (PMC10068768; doi:10.1002/open.202200266)
Supplement: Supplementary file 1 — Supporting Information [file OPEN-12-e202200266-s001.pdf]

ChemistryOpen

Supporting Information

Engineering *Saccharomyces cerevisiae* for the *de novo* Production of Halogenated Tryptophan and Tryptamine Derivatives

Nicholas Milne,* Javier Sáez-Sáez, Annette Munch Nielsen, Jane Dannow Dyekjær, Daniela Rago, Mette Kristensen, Tune Wulff, and Irina Borodina*

Table S1. Binana2 calculated interactions for docking of L-tryptophan and 5-halo-tryptophan in SrPyrH, L-Tryptophan and 6-halo-tryptophan in SttH, and L-tryptophan and 7-halo-tryptophan in LaRebH. Docking is carried out with either Br or Cl in each of the structures.

Protein	Interactions	Molecule	Molecule	Protein	Molecule	Molecule
**SrPyrH-Br**		**L-Tryptophan**	**5-Br-Tryptophan**	**SrPyrH-Cl**	**L-Tryptophan**	**5-Cl-Tryptophan**
	Hydrophobic	Phe49, Ile78, His92, Phe94, Phe451, Tyr454	Phe49, Thr51, His92, Phe94, Phe451, Tyr454		Phe49, Ile78, His92, Phe94, Phe451, Tyr454	Phe49, Thr51, His92, Phe94, Phe451, Tyr454
	Salt bridge	-	-		-	-
	Hydrogen bond	Amine N-Ser50 NH 3.4 Å Amine NH-Ser50 OG 3.4 Å	Amine NH-Ser50 OG 3.2 Å		Amine N-Ser50 NH 3.5 Å Amine NH-Ser50 OG 3.4 Å	Amine N-Ser50 NH 3.3 Å Amine NH-Ser50 OG 3.3 Å
	Halogen bond	-	Br-Ser355 OG 4.7 Å		-	Cl-Ser355 O 5.5 Å Cl-Ser355 OG 4.5 Å
	Cation-π-interaction	-	-		-	-
	π-π-stacking	Pyrrole-His92 4.3 Å Benzene-His92 4.7 Å	Benzene-His92 4.4 Å		Pyrrole-His92 4.3 Å Benzene-His92 4.7 Å	Benzene-His92 4.4 Å
	T-stacking	Pyrrole-Phe49 5.5 Å	Pyrrole-Phe49 5.4 Å		Pyrrole-Phe49 5.8 Å	Pyrrole-Phe49 5.4 Å
**SttH-Br**		**L-Tryptophan**	**6-Br-tryptophan**	**SttH-Cl**	**L-Tryptophan**	**6-Cl-tryptophan**
	Hydrophobic	Phe53, Ser54, Lys79, Val82, His96, Pro97, Phe98, Glu363, Pro461	Phe53, Ser54, Lys79, Val82, His96, Pro97, Phe98, Glu363, Pro461		Phe53, Ser54, Ala81, Val82, His96, Phe98, Glu363, Leu460, Tyr463	Phe53, Ser54,Lys79, Val82, His96, Pro97, Phe98, Glu363, Pro461
	Salt bridge	-	-			-
	Hydrogen bond	Carboxylate O-Tyr463 OH 2.5 Å	Carboxylate O-Tyr463 OH 2.5 Å		Carboxylate O-Tyr463 OH 3.1 Å	Carboxylate O-Tyr463 OH 2.5 Å
	Halogen bond	-	-		-	-
	Cation-π-interaction	Amine-Phe53 3.0 Å Pyrrole-His96 3.0 Å	Amine-Phe53 3.1 Å Pyrrole-His96 3.0 Å		Pyrrole-His96 3.0 Å Benzene-His96 3.2 Å	Amine-Phe93 3.1 Å Pyrrole-His96 3.0 Å
	π-π-stacking:	Pyrrole-His96 3.1 Å Benzene-His96 3.9 Å	Pyrrole-His96 3.1 Å Benzene-His96 3.9 Å		-	Pyrrole-His96 3.0 Å Benzene-His96 3.9 Å
	T-stacking:	Pyrrole-Phe98 5.0 Å	Pyrrole-Phe98 5.0 Å		-	Pyrrole-Phe98 5.0 Å
**LaRebH-Br**		**L-Tryptophan**	**7-Br-tryptophan**	**LaRebH-Cl**	**L-Tryptophan**	**7-Cl-tryptophan**
	Hydrophobic	Ile52, Lys79, Ile82, His109, Ser110, Phe111, Glu357, Ser358, Tyr454, Asn470	Ile52, Lys79, Ile82, His109, Ser110, Phe111, Glu357, Tyr454, Asn470		Ile52, Lys79, Ile82, His109, Ser110, Phe111, Glu357, Ser358, Tyr454, Asn470	Ile52, Lys79, Ile82, His109, Ser110, Phe111, Glu357, Tyr454, Asn470
	Salt bridge	Amine-Glu461 5.1 Å	Amine-Glu461 4.8 Å		Amine-Glu461 5.1 Å	Amine-Glu461 5.0 Å
	Hydrogen bond	Indole NH-His109 0 3.3 Å	Indole NH-His109 0 3.5 Å		Indole NH-His109 0 3.2 Å	Indole NH-His109 O 3.4 Å

	Halogen bond	-	Br-Ile82 N 4.8 Å		-	Br-Ile82 N 4.7Å
	Cation-π-interaction	Pyrrole-His109 3.4 Å	Pyrrole-His109 3.6 Å		Pyrrole-His109 3.5 Å	Pyrrole-His109 3.5 Å
	π-π-stacking:	Pyrrole-His109 3.6 Å<br>Benzene His109 4.5 Å	Pyrrole-His109 3.6 Å<br>Benzene His109 4.5 Å<br>Benzene Phe111 4.5 Å		Pyrrole-His109 3.5Å<br>Benzene His109 4.5 Å	Pyrrole-His109 3.6 Å<br>Benzene His109 4.6 Å
	T-stacking:	-	-		-	-

Table S2: Binana2 calculated interactions for docking of 5-, 6- and 7-halo-tryptophan and 5-, 6- and 7-halo-tryptamine to CrTDC.

**CrTDC**	**5-Br-tryptophan**	**5-Br-tryptamine**	**5-Cl-tryptophan**	**5-Cl-tryptamine**
Hydrophobic	Trp92A, Phe100A, His318A, LLP319A, Val122B, Phe124B	Phe100A, Phe101A, Thr262A, His318A, Val122B, Phe124B, Thr369B, Gly370B	Phe100A, His318A, LLP319A, Leu325A, Val122B, Phe124B, Thr369B, Gly370B	Phe100A, His318A, LLP319A, Val122B, Phe124B, Thr369B, Gly370B
Salt bridge	-	-	-	-
Hydrogen bond	Indole N-Phe101A NH 3.6 Å<br>Indole NH-Phe101 O 2.8 Å	Amine NH -Phe101A N 3.7 Å<br>Indole NH-Gly370B N 3.7 Å	Indole NH-Gly370B N 3.1 Å	Indole NH-Gly370B N 3.6 Å<br>Phe101A N Amine NH 3.9
Halogen bond	-	Br-Pro102A O 5.3 Å	Cl Pro102A O 4.8 Å<br>Cl-Ala103A N 5.2 Å	Cl-Pro102A 5.0 Å<br>Cl-Ala103A 5.4 Å
Cation-π-interaction	Pyrrole-LLP 4.6 Å<br>Benzene-LLP 4.3 Å	Pyrrole-LLP 4.5Å<br>Benzene-LLP 4.4 Å	-	Pyrrole-LLP 4.5 Å<br>Benzene-LLP 4.4 Å
π-π-stacking:	-	Pyrrole-Phe124B 4.9 Å	-	Pyrrole-Phe124B 4.9 Å
T-stacking:	-	Pyrrole-LLP Pyridine 6.1 Å	-	Pyrrole-LLP Pyridine 6.3 Å
**CrTDC**	**6-Br-tryptophan**	**6-Br-tryptamine**	**6-Cl-tryptophan**	**6-Cl-tryptamine**
Hydrophobic	Phe101A, LLP319A, Val122B, Thr369B	Trp92A, Phe101A, His318A, LLP319A, Phe124B, Thr369B, Gly370B	Trp92A, Phe101A, LLP319A, Val122B, Thr369B	Trp92A, Phe100A, Phe101A, Thr262A, LLP319A, Val122B, Thr369B
Salt bridge	-	-	-	-
Hydrogen bond	Indole NH-Gly370B N 3.6 Å	-	-	Amine NH-Phe100A O 3.9 Å
Halogen bond	-	Br-His318A O 4.6 Å	Cl-Ala103A N 5.4 Å	-
Cation-π-interaction	Benzene-LLP319A 3.9 Å	Benzene-LLP319A 4.1 Å<br>Amine-Phe124B 5.5 Å	Benzene-LLP319A 4.0 Å	Amine-Phe101A 4.1 Å<br>Benzene-LLP319A 4.2 Å<br>Pyrrole-LLP319A 4.3 Å
π-π-stacking:	-	-	-	-
T-stacking:	-	Pyrrole-LLP Pyridine 6.5Å	-	-
**CrTDC**	**7-Br-tryptophan**	**7-Br-tryptamine**	**7-Cl-tryptophan**	**7-Cl-tryptamine**
Hydrophobic	Phe101A, LLP319A, Val122B, Thr369B	Trp92A, Phe100A, Phe101A, His318A, LLP319A, Val122B, Phe124B, Thr369B	Phe101A, LLP319A, Val122B, Phe124B, Thr369B	Trp92A, Phe100A, Phe101A, His318A, LLP319A, Val122B, Phe124B, Thr369B
Salt bridge	-	-	-	-
Hydrogen bond	-	Amine NH-Phe101A N 3.8 Å<br>Indole NH-Gly370B N 4.0 Å	-	Amine NH-Phe101A N 3.8Å<br>Indole NH-Gly370B N 3.9 Å
Halogen bond	-	-	-	-
Cation-π-interaction	Benzene-LLP319A 3.9 Å	Benzene-LLP319A 4.5 Å	Benzene-LLP319A 4.0 Å	Benzene-LLP319A 4.6 Å

		Pyrrole-LLP319A 4.4 Å		Pyrrole-LLP319A 4.4 Å
π-π-stacking:	-	Pyrrole-Phe124B 5.0 Å	-	Pyrrole-Phe124B 5.0 Å
T-stacking:	-	-	-	-

Table S3. DNA sequences of synthetic genes codon optimized for *S. cerevisiae*.

SrPyrH	ATGATCAGATCTGTTGTTATCGTTGGTGGTGGTACTGCTGGTTGGATGACTGCTTCTTACTTGAAGGCTGCTTTCGAC<br>GACAGAATCGACGTTACTTTGGTTGAATCTGGTAACGTTAGAAGAATCGGTGTTGGTGAAGCTACTTTCTCTACTGTTA<br>GACACTTCTTCGACTACTTGGGTTTGGACGAAAGAGAATGGTTGCCAAGATGTGCTGGTGGTTACAAGTTGGGTATCA<br>GATTCGAAAACTGGTCTGAACCAGGTGAATACTTCTACCACCCATTCGAAAGATTGAGAGTTGTTGACGGTTTCAACAT<br>GGCTGAATGGTGGTTGGCTGTTGGTGACAGAAGAACTTCTTTCTCTGAAGCTTGTTACTTGACTCACAGATTGTGTGA<br>AGCTAAGAGAGCTCCAAGAATGTTGGACGGTTCTTTGTTCGCTTCTCAAGTTGACGAATCTTTGGGTAGATCTACTTTG<br>GCTGAACAAAGAGCTCAATTCCCATACGCTTACCACTTCGACGCTGACGAAGTTGCTAGATACTTGTCTGAATACGCT<br>ATCGCTAGAGGTGTTAGA<br>CACGTTGTTGACGACGTTCAACACGTTGGTCAAGACGAAAGAGGTTGGATCTCTGGTGTTCACACTAAGCAACACGGT<br>GAAATCTCTGGTGACTTGTTCGTTGACTGTACTGGTTTCAGAGGTTTGTTGATCAACCAAACTTTGGGTGGTAGATTCC<br>AATCTTTCTCTGACGTTTTGCCAAACAACAGAGCTGTTGCTTTGAGAGTTCCAAGAGAAAACGACGAAGACATGAGAC<br>CATACACTACTGCTACTGCTATGTCTGCTGGTTGGATGTGGACTATCCCATTGTTCAAGAGAGACGGTAACGGTTACG<br>TTTACTCTGACGAATTCATCTCTCCAGAAGAAGCTGAAAGAGAATTGAGATCTACTGTTGCTCCAGGTAGAGACGACTT<br>GGAAGCTAACCACATCCAAATGAGAATCGGTAGAAACGAAAGAACTTGGATCAACAACTGTGTTGCTGTTGGTTTGTC<br>TGCTGCTTTCGTTGAACCATTGGAATCTACTGGTATCTTCTTCATCCAACACGCTATCGAACAATTGGTTAAGCACTTC<br>CCAGGTGAAAGATGGGACCCAGTTTTGATCTCTGCTTACAACGAAAGAATGGCTCACATGGTTGACGGTGTTAAGGAA<br>TTCTTGGTTTTGCACTACAAGGGTGCTCAAAGAGAAGACACTCCATACTGGAAGGCTGCTAAGACTAGAGCTATGCCA<br>GACGGTTTGGCTAGAAAGTTGGAATTGTCTGCTTCTCACTTGTTGGACGAACAAACTATCTACCCATACTACCACGGTT<br>TCGAAACTTACTCTTGGATCACTATGAACTTGGGTTTGGGTATCGTTCCAGAAAGACCAAGACCAGCTTTGTTGCACAT<br>GGACCCAGCTCCAGCTTTGGCTGAATTCGAAAGATTGAGAAGAGAAGGTGACGAATTGATCGCTGCTTTGCCATCTTG<br>TTACGAATACTTGGCTTCTATCCAATAG
SttH	ATGAACACTAGAAACCCAGACAAGGTTGTTATCGTTGGTGGTGGTACTGCTGGTTGGATGACTGCTTCTTACTTGAAG<br>AAGGCTTTCGGTGAAAGAGTTTCTGTTACTTTGGTTGAATCTGGTACTATCGGTACTGTTGGTGTTGGTGAAGCTACTT<br>TCTCTGACATCAGACACTTCTTCGAATTCTTGGACTTGAGAGAAGAAGAATGGATGCCAGCTTGTAACGCTACTTACAA<br>GTTGGCTGTTAGATTCCAAGACTGGCAAAGACCAGGTCACCACTTCTACCACCCATTCGAACAAATGAGATCTGTTGA<br>CGGTTTCCCATTGACTGACTGGTGGTTGCAAAACGGTCCAACTGACAGATTCGACAGAGACTGTTTCGTTATGGCTTC<br>TTTGTGTGACGCTGGTAGATCTCCAAGATACTTGAACGGTTCTTTGTTGCAACAAGAATTCGACGAAAGAGCTGAAGA<br>ACCAGCTGGTTTGACTATGTCTGAACACCAAGGTAAGACTCAATTCCCATACGCTTACCACTTCGAAGCTGCTTTGTTG<br>GCTGAATTCTTGTCTGGTTACTCTAAGGACAGAGGTGTTAAGCACGTTGTTGACGAAGTTTTGGAAGTTAAGTTGGAC<br>GACAGAGGTTGGATCTCTCACGTTGTTACTAAGGAACACGGTGACATCGGTGGTGACTTGTTCGTTGACTGTACTGGT<br>TTCAGAGGTGTTTTGTTGAACCAAGCTTTGGGTGTTCCATTCGTTTCTTACCAAGACACTTTGCCAAACGACTCTGCTG<br>TTGCTTTGCAAGTTCCATTGGACATGGAAGCTAGAGGTATCCCACCATACACTAGAGCTACTGCTAAGGAAGCTGGTT<br>GGATCTGGACTATCCCATTGATCGGTAGAATCGGTACTGGTTACGTTTACGCTAAGGACTACTGTTCTCCAGAAGAAG<br>CTGAAAGAACTTTGAGAGAATTCGTTGGTCCAGAAGCTGCTGACGTTGAAGCTAACCACATCAGAATGAGAATCGGTA<br>GATCTGAACAATCTTGGAAGAACAACTGTGTTGCTATCGGTTTGTCTTCTGGTTTCGTTGAACCATTGGAATCTACTGG<br>TATCTTCTTCATCCACCACGCTATCGAACAATTGGTTAAGCACTTCCCAGCTGGTGACTGGCACCCACAATTGAGAGC<br>TGGTTACAACTCTGCTGTTGCTAACGTTATGGACGGTGTTAGAGAATTCTTGGTTTTGCACTACTTGGGTGCTGCTAGA<br>AACGACACTAGATACTGGAAGGACACTAAGACTAGAGCTGTTCCAGACGCTTTGGCTGAAAGAATCGAAAGATGGAA<br>GGTTCAATTGCCAGACTCTGAAAACGTTTTCCCATACTACCACGGTTTGCCACCATACTCTTACATGGCTATCTTGTTG<br>GGTACTGGTGCTATCGGTTTGAGACCATCTCCAGCTTTGGCTTTGGCTGACCCAGCTGCTGCTGAAAAGGAATTCACT<br>GCTATCAGAGACAGAGCTAGATTCTTGGTTGACACTTTGCCATCTCAATACGAATACTTCGCTGCTATGGGTCAAAGA<br>GTTTAG
LaRebH	ATGTCTGGTAAGATCGACAAGATCTTGATCGTTGGTGGTGGTACTGCTGGTTGGATGGCTGCTTCTTACTTGGGTAAG<br>GCTTTGCAAGGTACTGCTGACATCACTTTGTTGCAAGCTCCAGACATCCCAACTTTGGGTGTTGGTGAAGCTACTATC<br>CCAAACTTGCAAACTGCTTTCTTCGACTTCTTGGGTATCCCAGAAGACGAATGGATGAGAGAATGTAACGCTTCTTACA<br>AGGTTGCTATCAAGTTCATCAACTGGAGAACTGCTGGTGAAGGTACTTCTGAAGCTAGAGAATTGGACGGTGGTCCAG<br>ACCACTTCTACCACTCTTTCGGTTTGTTGAAGTACCACGAACAAATCCCATTGTCTCACTACTGGTTCGACAGATCTTA<br>CAGAGGTAAGACTGTTGAACCATTCGACTACGCTTGTTACAAGGAACCAGTTATCTTGGACGCTAACAGATCTCCAAG<br>AAGATTGGACGGTTCTAAGGTTACTAACTACGCTTGGCACTTCGACGCTCACTTGGTTGCTGACTTCTTGAGAAGATT<br>CGCTACTGAAAAGTTGGGTGTTAGACACGTTGAAGACAGAGTTGAACACGTTCAAAGAGACGCTAACGGTAACATCGA<br>ATCTGTTAGAACTGCTACTGGTAGAGTTTTCGACGCTGACTTGTTCGTTGACTGTTCTGGTTTCAGAGGTTTGTTGATC<br>AACAAGGCTATGGAAGAACCATTCTTGGACATGTCTGACCACTTGTTGAACGACTCTGCTGTTGCTACTCAAGTTCCA<br>CACGACGACGACGCTAACGGTGTTGAACCATTCACTTCTGCTATCGCTATGAAGTCTGGTTGGACTTGGAAGATCCCA<br>ATGTTGGGTAGATTCGGTACTGGTTACGTTTACTCTTCTAGATTCGCTACTGAAGACGAAGCTGTTAGAGAATTCTGTG<br>AAATGTGGCACTTGGACCCAGAAACTCAACCATTGAACAGAATCAGATTCAGAGTTGGTAGAAACAGAAGAGCTTGGG<br>TTGGTAACTGTGTTTCTATCGGTACTTCTTCTTGTTTCGTTGAACCATTGGAATCTACTGGTATCTACTTCGTTTACGCT<br>GCTTTGTACCAATTGGTTAAGCACTTCCCAGACAAGTCTTTGAACCCAGTTTTGACTGCTAGATTCAACAGAGAAATCG<br>AAACTATGTTCGACGACACTAGAGACTTCATCCAAGCTCACTTCTACTTCTCTCCAAGAACTGACACTCCATTCTGGAG<br>AGCTAACAAGGAATTGAGATTGGCTGACGGTATGCAAGAAAAGATCGACATGTACAGAGCTGGTATGGCTATCAACGC<br>TCCAGCTTCTGACGACGCTCAATTGTACTACGGTAACTTCGAAGAAGAATTCAGAAACTTCTGGAACAACTCTAACTAC<br>TACTGTGTTTTGGCTGGTTTGGGTTTGGTTCCAGACGCTCCATCTCCAAGATTGGCTCACATGCCACAAGCTACTGAA

	TCTGTTGACGAAGTTTTCGGTGCTGTTAAGGACAGACAAAGAAACTTGTTGGAAACTTTGCCATCTTTGCACGAATTCTTGAGACAACAACACGGTAGATAG
LaRebF	ATGACTATCGAATTCGACAGACCAGGTGCTCACGTTACTGCTGCTGACCACAGAGCTTTGATGTCTTTGTTCCCAACTGGTGTTGCTGTTATCACTGCTATCGACGAAGCTGGTACTCCACACGGTATGACTTGTACTTCTTTGACTTCTGTTACTTTGGACCCACCAACTTTGTTGGTTTGTTTGAACAGAGCTTCTGGTACTTTGCACGCTGTTAGAGGTGGTAGATTCGGTGTTAACTTGTTGCACGCTAGAGGTAGAAGAGCTGCTGAAGTTTTCTCTACTGCTGTTCAAGACAGATTCGGTGAAGTTAGATGGGAACACTCTGACGTTACTGGTATGCCATGGTTGGCTGAAGACGCTCACGCTTTCGCTGGTTGTGTTGTTAGAAAGTCTACTGTTGTTGGTGACCACGAAATCGTTTTGGGTGAAGTTCACGAAGTTGTTAGAGAACACGACTTGCCATTGTTGTACGGTATGAGAGAATTCGCTGTTTGGACTCCAGAAGGTTAG

Table S4. List of *S. cerevisiae* strains used in this study.

Name	Parental strain	Added DNA element	Relevant genotype	Source
CEN.PK113-7D	-	-	Mata *MAL2-8c SUC2 URA3 HIS3 LEU2 TRP1*	[1]
ST7574	CEN.PK113-7D	pCfB2312	*MAL2-8c SUC2 URA3 HIS3 LEU2 TRP1* + pCfB2312 (Cas9)	[2]
ST9336	ST7574	pCfB8881	*MAL2-8c SUC2 URA3 HIS3 LEU2 TRP1 XI-3:: CrTDC*	This study
ST9759	ST7574	pCfB9331	*MAL2-8c SUC2 URA3 HIS3 LEU2 TRP1 XI-1:: SrPyrH, LaRebF*	This study
ST9760	ST7574	pCfB9332	*MAL2-8c SUC2 URA3 HIS3 LEU2 TRP1 XI-1:: SrPyrH*	This study
ST9761	ST7574	pCfB9333	*MAL2-8c SUC2 URA3 HIS3 LEU2 TRP1 XII-5:: SttH, LaRebF*	This study
ST9762	ST7574	pCfB9334	*MAL2-8c SUC2 URA3 HIS3 LEU2 TRP1 XII-5:: SttH*	This study
ST9763	ST7574	pCfB9332, pCfB9333	*MAL2-8c SUC2 URA3 HIS3 LEU2 TRP1 XI-1:: SrPyrH XII-5:: SttH, LaRebF*	This study
ST9764	ST9336	pCfB9331	*MAL2-8c SUC2 URA3 HIS3 LEU2 TRP1 XI-3:: CrTDC XI-1:: SrPyrH, LaRebF*	This study
ST9765	ST9336	pCfB9332	*MAL2-8c SUC2 URA3 HIS3 LEU2 TRP1 XI-3:: CrTDC XI-1:: SrPyrH*	This study
ST9766	ST9336	pCfB9333	*MAL2-8c SUC2 URA3 HIS3 LEU2 TRP1 XI-3:: CrTDC XII-5:: SttH, LaRebF*	This study
ST9767	ST9336	pCfB9334	*MAL2-8c SUC2 URA3 HIS3 LEU2 TRP1 XI-3:: CrTDC XII-5:: SttH*	This study
ST9768	ST9336	pCfB9332, pCfB9333	*MAL2-8c SUC2 URA3 HIS3 LEU2 TRP1 XI-3:: CrTDC XI-1:: SrPyrH XII-5:: SttH, LaRebF*	This study
ST10071	ST9336	pCfB9712	*MAL2-8c SUC2 URA3 HIS3 LEU2 TRP1 XI-3:: CrTDC XII-4: :LaRebH*	This study
ST10072	ST9768	pCfB9712	*MAL2-8c SUC2 URA3 HIS3 LEU2 TRP1 XI-3:: CrTDC XI-1:: SrPyrH XII-5:: SttH, LaRebF XII-4:: LaRebH*	This study
ST10073	ST9766	pCfB9712	*MAL2-8c SUC2 URA3 HIS3 LEU2 TRP1 XI-3:: CrTDC XII-5:: SttH, LaRebF XII-4:: LaRebH*	This study
ST10290	ST7574	pCfB9713	*MAL2-8c SUC2 URA3 HIS3 LEU2 TRP1 XII-4:: LaRebH, LaRebF*	This study
ST10352	ST9336	pCfB9713	*MAL2-8c SUC2 URA3 HIS3 LEU2 TRP1 XI-3:: CrTDC XII-4:: LaRebH, LaRebF*	This study

Table S5. List of plasmids used in this study.

Name	Parent plasmid, BioBricks	Relevant characteristics	Origin
**Templates for PCR amplification**			
p1977	-	*pTDH3-pTEF1* fused promoters	[3]
pCfB8793	-	CrTDC template	[4]
**gRNA plasmids for targeting genomic integration sites by CRISPR-Cas9**			
pCfB3043	-	2μm ori NatMX pSNR52-XI-1- gRNA-tSUP4	[3]
pCfB3045	-	2μm ori NatMX pSNR52-XI-3 gRNA-tSUP4	[3]
pCfB3049	-	2μm ori NatMX pSNR52-XII-4 gRNA-tSUP4	[3]

pCfB3050	-	2µm ori NatMX pSNR52-XII-5 gRNA-tSUP4	[3]
pCfB9077	pTAJAK-71, BB3959, BB4027	2µm ori NatMX pSNR52-XI-1 gRNA-tSUP4 pSNR52-XII-5 gRNA-tSUP4	This study
**Episomal yeast expression plasmids**			
pCfB2312		2µm ori *pTEF1->cas9* KanMX	[3]
pTAJAK-71		2µm ori NatMX	[3]
**Backbone plasmids for EasyClone-MarkerFree plasmid assembly**			
pCfB2904	-	pXI-3-USER	[3]
pCfB2909	-	pXII-5-USER	[3]
pCfB3036	-	pXI-1-USER	[3]
pCfB3040	-	pXII-4-USER	[3]
**Plasmids for integration into yeast genome**			
pCfB8881	pCfB2904, BB3816, BB8	*XI-3:: CrTDC<-pTEF1*	This study
pCfB9331	pCfB3036, BB4336, BB464, BB4338	*XI-1:: SrPyrH<-pTDH3-pTEF1->LaRebF*	This study
pCfB9332	pCfB3036, BB4336, BB410	*XI-1:: SrPyrH<-pTDH3*	This study
pCfB9333	pCfB2909, BB4337, BB464, BB4338	*XII-5:: SttH<-pTDH3-pTEF1->LaRebF*	This study
pCfB9334	pCfB2909, BB410	*XII-5:: SttH<-pTDH3*	This study
pCfB9712	pCfB3040, BB4441, BB410	*XII-4:: LaRebH<-pTDH3*	This study
pCfB9713	pCfB3040, BB4441, BB464, BB4338	*XII-4:: LaRebH<-pTDH3-pTEF1->LaRebF*	This study

Table S6. List of BioBricks used in this study.

Name	Description	Fwd primer	Rev primer	Template
BB8	*TEF1* promoter	PR-5	PR-6	p1977
BB410	*TDH3* promoter	PR-1852	PR-1853	p1977
BB464	*TDH3-TEF1* fused promoters	PR-1853	PR-1565	p1977
BB3816	*CrTDC* gene	PR-23893	PR-23894	pCfB8793
BB3959	XI-1 gRNA	PR-10525	PR-10530	pCfB3043
BB4027	XII-5 gRNA	PR-10526	PR-10529	pCfB3050
BB4336	*SrPyrH* gene	PR-26290	PR-26291	Synthetic DNA (GeneArt)
BB4337	*SttH* gene	PR-26292	PR-26293	Synthetic DNA (GeneArt)
BB4338	*LaRebF* gene	PR-26294	PR-26295	Synthetic DNA (GeneArt)
BB4441	*LaRebH* gene	PR-26840	PR-26841	Synthetic DNA (GeneArt)

Table S7. List of primers used in this study.

Name	Sequence (5'→3')	Purpose
**Primers for BioBrick amplification**		
PR-5	ACCTGCACUTTGTAATTAAAACTTAG	Fwd primer to amplify BB8
PR-6	CACGCGAUGCACACACCATAGCTTC	Rev primer to amplify BB8
PR-1565	ATGACAGAUTTGTAATTAAAACTTAG	Fwd primer to amplify BB464
PR-1852	CACGCGAUATAAAAAACACGCTTTTTCAG	Fwd primer to amplify BB410
PR-1853	ACCTGCACUTTTGTTTGTTTATGTGTGTTTATTC	Rev primer to amplify BB0410 and BB0464
PR-10525	CGTGCGAUAGGGAACAAAAGCTGGAGCT	Fwd primer to amplify BB3959
PR-10526	AGTGCAGGUAGGGAACAAAAGCTGGAGCT	Fwd primer to amplify BB4027
PR-10529	CACGCGAUTAACTAATTACATGACTCGA	Rev primer to amplify BB4027
PR-10530	ACCTGCACUTAACTAATTACATGACTCGA	Rev primer to amplify BB3959
PR-23893	AGTGCAGGUAAAACAATGGGTTCTATTGATTCTACCAACG	Fwd primer to amplify BB3816
PR-23894	CGTGCGAUTCAGGCTTCTTTCAACAAGTC	Rev primer to amplify BB3816

PR-26290	AGTGCAGGUAAAACAATGATCAGATCTGTTGTTATCGTTGGTG	Fwd primer to amplify BB4336
PR-26291	CGTGCGAUCTATTGGATAGAAGCCAAGTATTCG	Rev primer to amplify BB4336
PR-26292	AGTGCAGGUAAAACAATGAACACTAGAAACCCAG	Fwd primer to amplify BB4337
PR-26293	CGTGCGAUCTAAACTCTTTGACCCATAGC	Rev primer to amplify BB4337
PR-26294	ATCTGTCAUAAAACAATGACTATCGAATTCGACAGACC	Fwd primer to amplify BB4338
PR-26295	CACGCGAUCTAACCTTCTGGAGTCCAAAC	Rev primer to amplify BB4338
PR-26840	AGTGCAGGUAAAACAATGTCTGGTAAGATCGACAAG	Fwd primer to amplify BB4441
PR-26841	CGTGCGAUCTATCTACCGTGTTGTTGTCTC	Rev primer to amplify BB4441
**Primers for verification of correct EasyClone plasmid assembly**		
PR-22955	GACGGTAGGTATTGATTGTAATTCTG	*pTDH3* Fwd diagnostic PCR primer
PR-339	GCTCATTAGAAAGAAAGCATAGC	*pTEF1* Fwd diagnostic PCR primer
PR-224	GAAATTCGCTTATTTAGAAGTGTC	*tADH1* Rev diagnostic PCR primer
PR-225	CTCCTTCCTTTTCGGTTAGAG	*tCYC1* Rev diagnostic PCR primer
PR-23875	ACTGTTGGGAAGGGCGATC	gRNA cassette Fwd diagnostic PCR primer
PR-23876	AGCGCCCAATACGCAAAC	gRNA cassette Rev diagnostic PCR primer
**Primers for genotyping correct genomic integration of expression cassettes**		
PR-2221	GTTGACACTTCTAAATAAGCGAATTTC	Universal Rev primer binding in *S. cerevisiae* integration cassettes
PR-897	GAACTGACGTCGAAGGCTCT	Fwd primer for diagnostic PCR of EasyClone plasmid integration at XII-4 site
PR-898	CGTGAAATCTCTTTGCGGTAG	Rev primer for diagnostic PCR of EasyClone plasmid integration at XII-4 site
PR-899	CCACCGAAGTTGATTTGCTT	Fwd primer for diagnostic PCR of EasyClone plasmid integration at XII-5 site
PR-900	GTGGGAGTAAGGGATCCTGT	Rev primer for diagnostic PCR of EasyClone plasmid integration at XII-5 site
PR-907	CTTAATGGGTAGTGCTTGACACG	Fwd primer for diagnostic PCR of EasyClone plasmid integration at XI-1 site
PR-908	GAAGACCCATGGTTCCAAGGA	Rev primer for diagnostic PCR of EasyClone plasmid integration at XI-1 site
PR-911	GTGCTTGATTTGCGTCATTC	Fwd primer for diagnostic PCR of EasyClone plasmid integration at XI-3 site
PR-912	CACATTGAGCGAATGAAACG	Rev primer for diagnostic PCR of EasyClone plasmid integration at XI-3 site

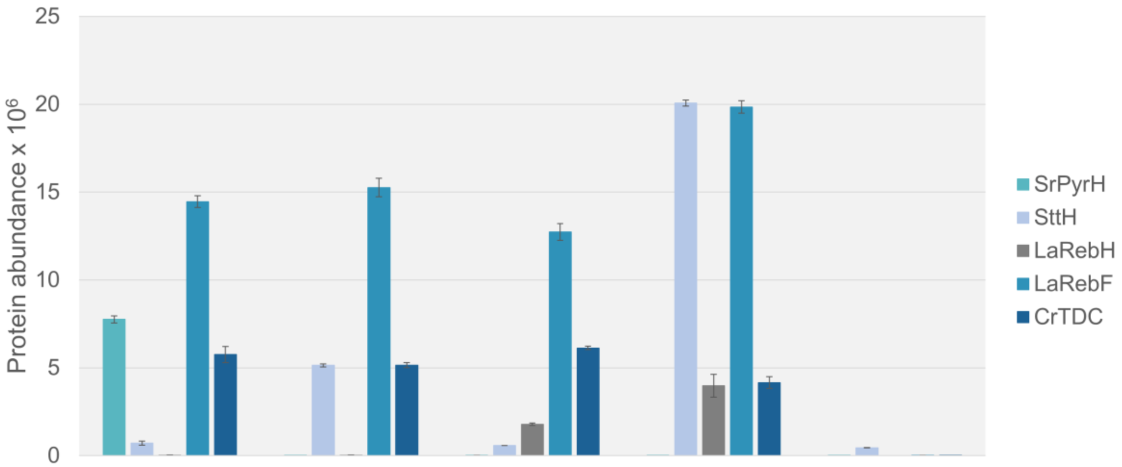

	ST9764	ST9766	ST10352	ST10073	ST7574
SrPyrH	+	−	−	−	−
SttH	−	+	−	+	−
LaRebH	−	−	+	+	−
LaRebF	+	+	+	+	−
CrTDC	+	+	+	+	−

Figure S1. Normalized protein abundance of heterologous proteins in multiple *S. cerevisiae* engineered strains. "+" and "−" symbols indicate the presence or absence of the corresponding genetic modification, respectively. Error bars represent the standard deviation from two biological replicates. Complete dataset, including normalized abundances of yeast native proteins within the proteome, is shown in the Supplementary File 1.

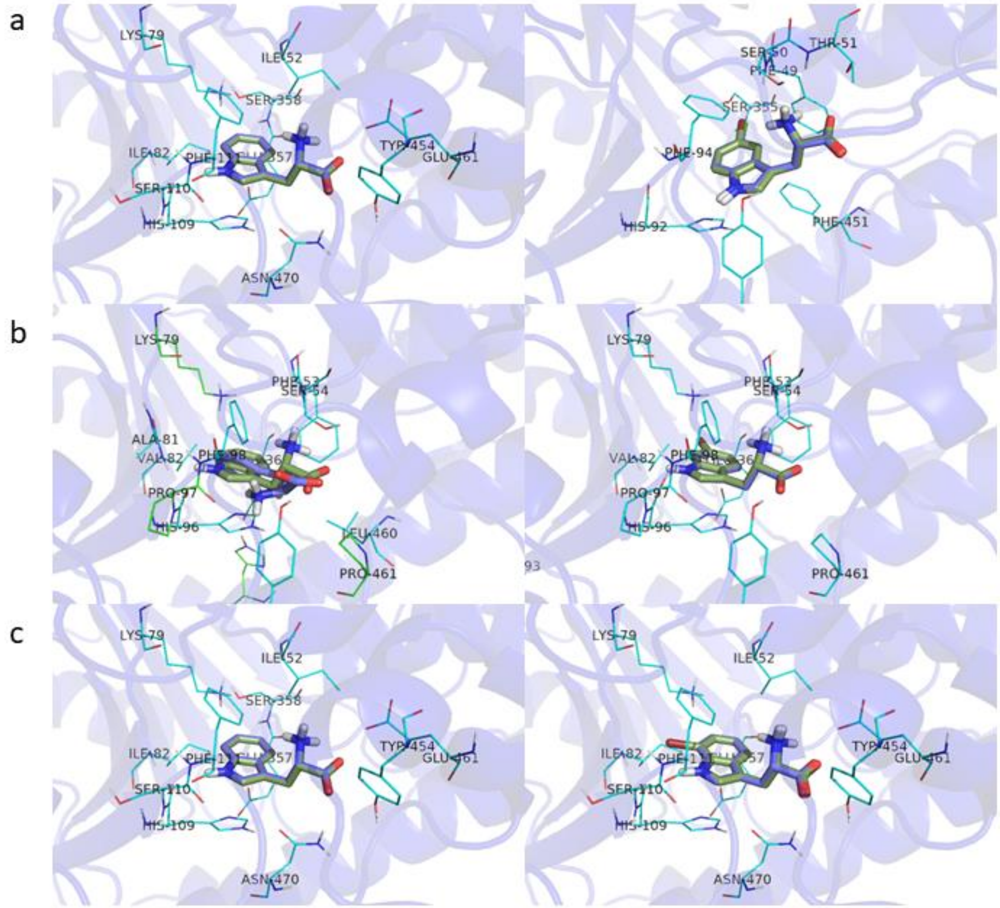

Figure S2. L-tryptophan and corresponding halotryptophan docked to a) SrPyrH, b) SttH, c) LaRebH. Chlorotryptophan is shown with green sticks and bromotryptophan is shown as slate blue.

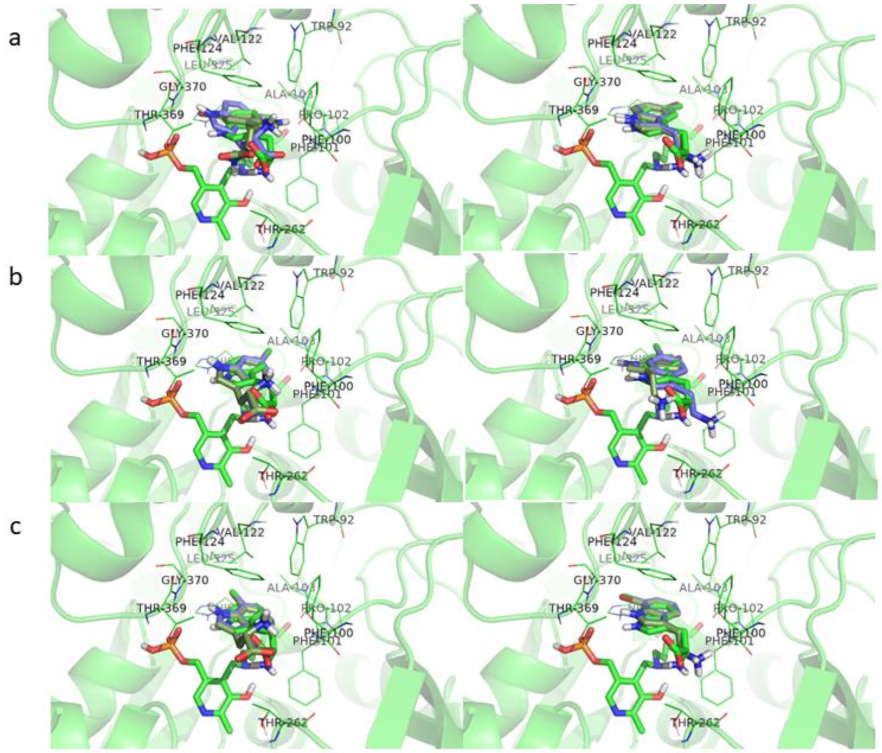

Figure S3. Docking of halotryptophan and halotryptamine to CrTDC. Chlorotryptophan is shown with green sticks and bromotryptophan is shown as slate blue. The crystal structure cofactor LLP and bound L-tryptophan is shown in bright green. a) 5-halotryptophan to the left-hand side, 5-halotryptamine to the right-hand side. b) 6-halotryptophan to the left-hand side, 6-halotryptamine to the right-hand side. c) 7-halotryptophan to the left-hand side, 7-halotryptamine to the right-hand side.

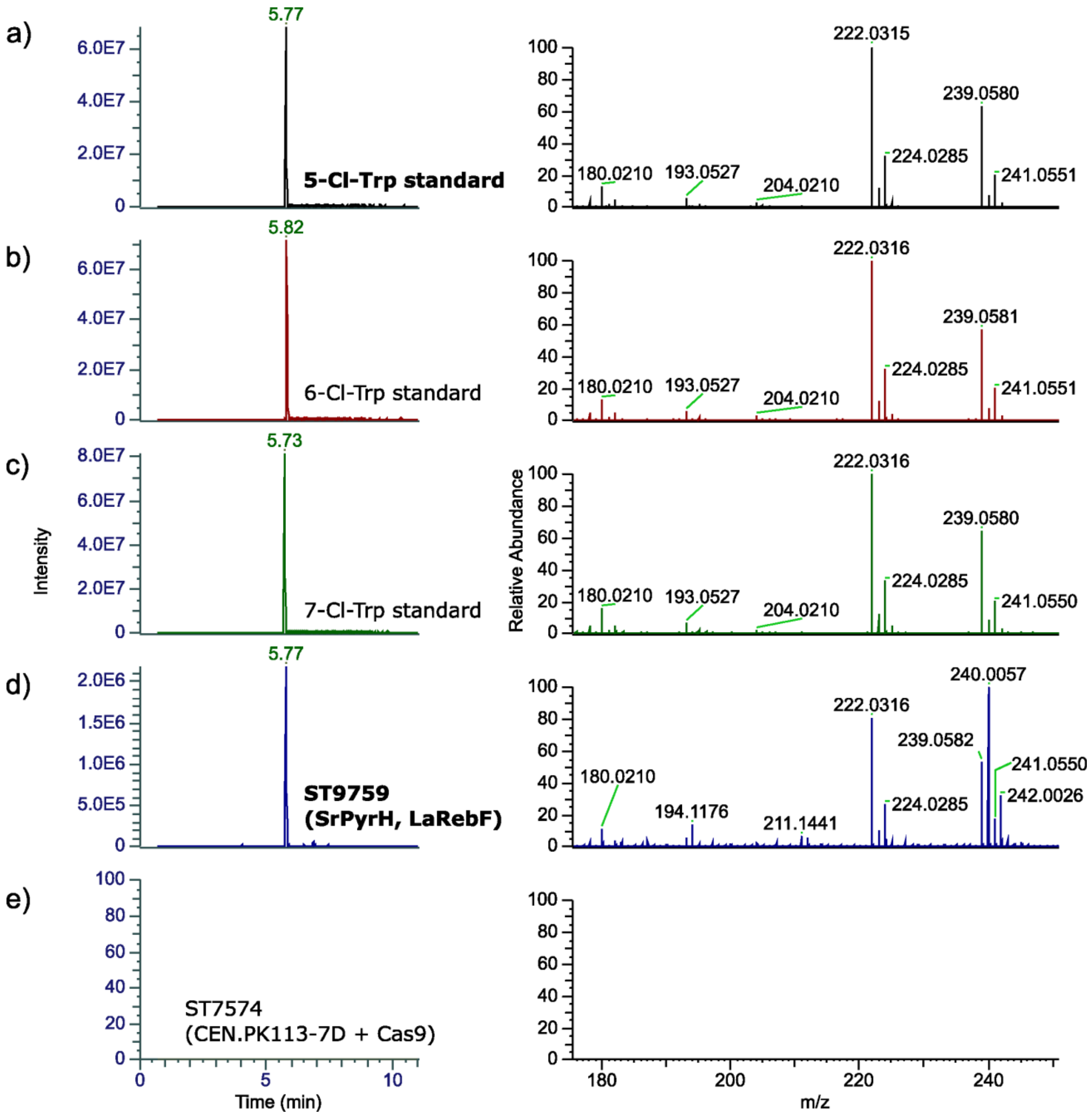

Figure S4. Production of 5-chlorotryptophan in engineered *S. cerevisiae* strains. LC-MS extracted ion chromatograms and corresponding mass spectra of the main peak for a) 5-chlorotryptophan standard, b) 6-chlorotryptophan standard, c) 7-chlorotryptophan standard, d) ST9759 (*SrPyrH*, *LaRebF*), e) ST7574 (Wild-type control, CEN.PK113-7D + Cas9). Theoretical m/z of $[M+H]^+$ and $[M+H-NH_3]^+$ adducts with ^{35}Cl and ^{37}Cl isotopes is 239.0582/222.0316 (most abundant) and 241.0552/224.0287, respectively. Note the presence of chlorinated xanthurenic acid as the main halogenated product, with observed m/z $[M+H]^+$ of 240.0057 (^{35}Cl) and 242.0026 (^{37}Cl).

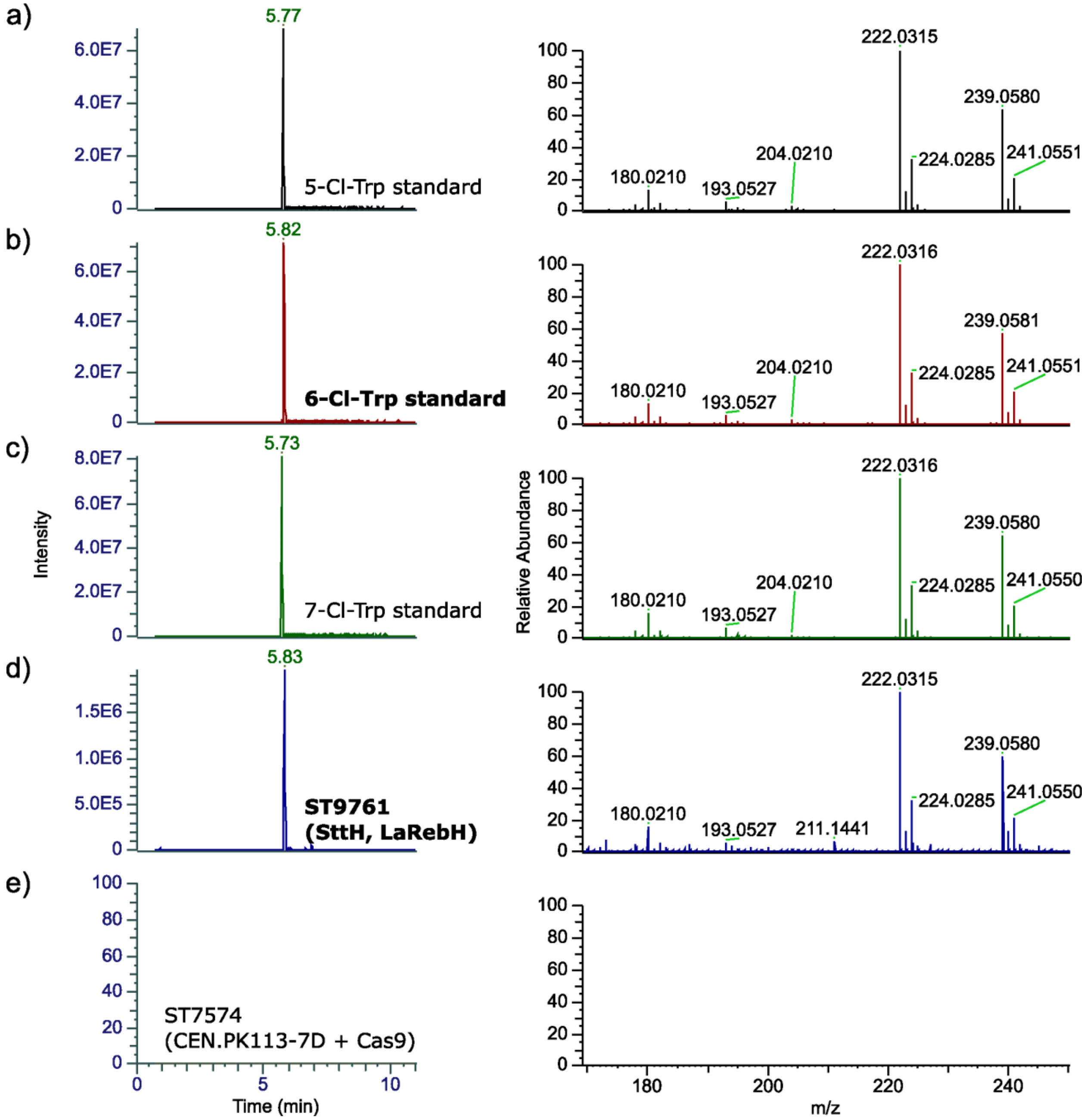

Figure S5. Production of 6-chlorotryptophan in engineered *S. cerevisiae* strains. LC-MS extracted ion chromatograms and corresponding mass spectra of the main peak for a) 5-chlorotryptophan standard, b) 6-chlorotryptophan standard, c) 7-chlorotryptophan standard, d) ST9761 (*SttH*, *LaRebF*), e) ST7574 (Wild-type control, CEN.PK113-7D + Cas9). Theoretical m/z of $[M+H]^+$ and $[M+H-NH_3]^+$ adducts with ^{35}Cl and ^{37}Cl isotopes is 239.0582/222.0316 (most abundant) and 241.0552/224.0287, respectively.

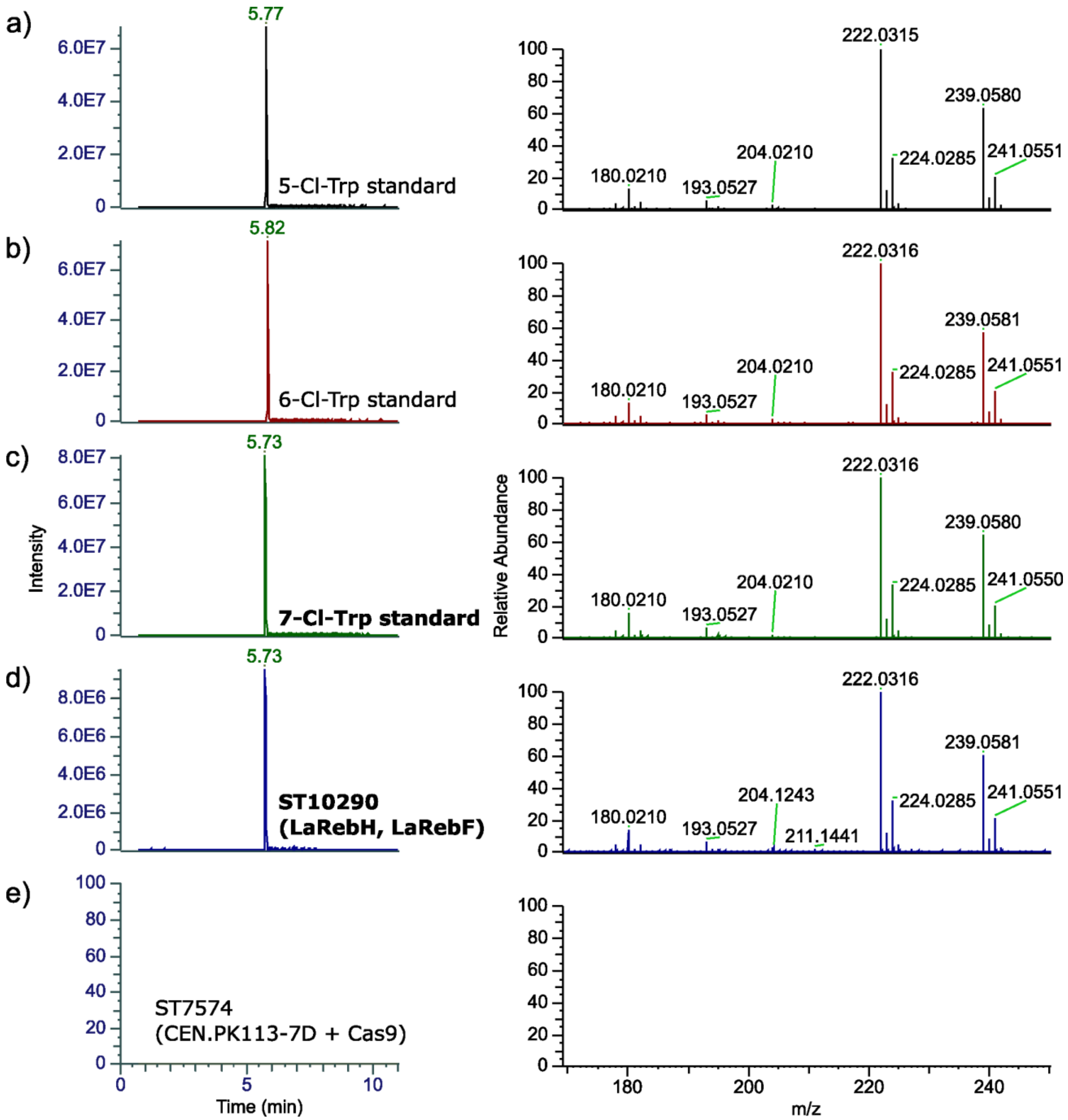

Figure S6. Production of 7-chlorotryptophan in engineered *S. cerevisiae* strains. LC-MS extracted ion chromatograms and corresponding mass spectra of the main peak for a) 5-chlorotryptophan standard, b) 6-chlorotryptophan standard, c) 7-chlorotryptophan standard, d) ST10290 (*LaRebH*, *LaRebF*), e) ST7574 (Wild-type control, CEN.PK113-7D + Cas9). Theoretical m/z of $[M+H]^+$ and $[M+H-NH_3]^+$ adducts with ^{35}Cl and ^{37}Cl isotopes is 239.0582/222.0316 (most abundant) and 241.0552/224.0287, respectively.

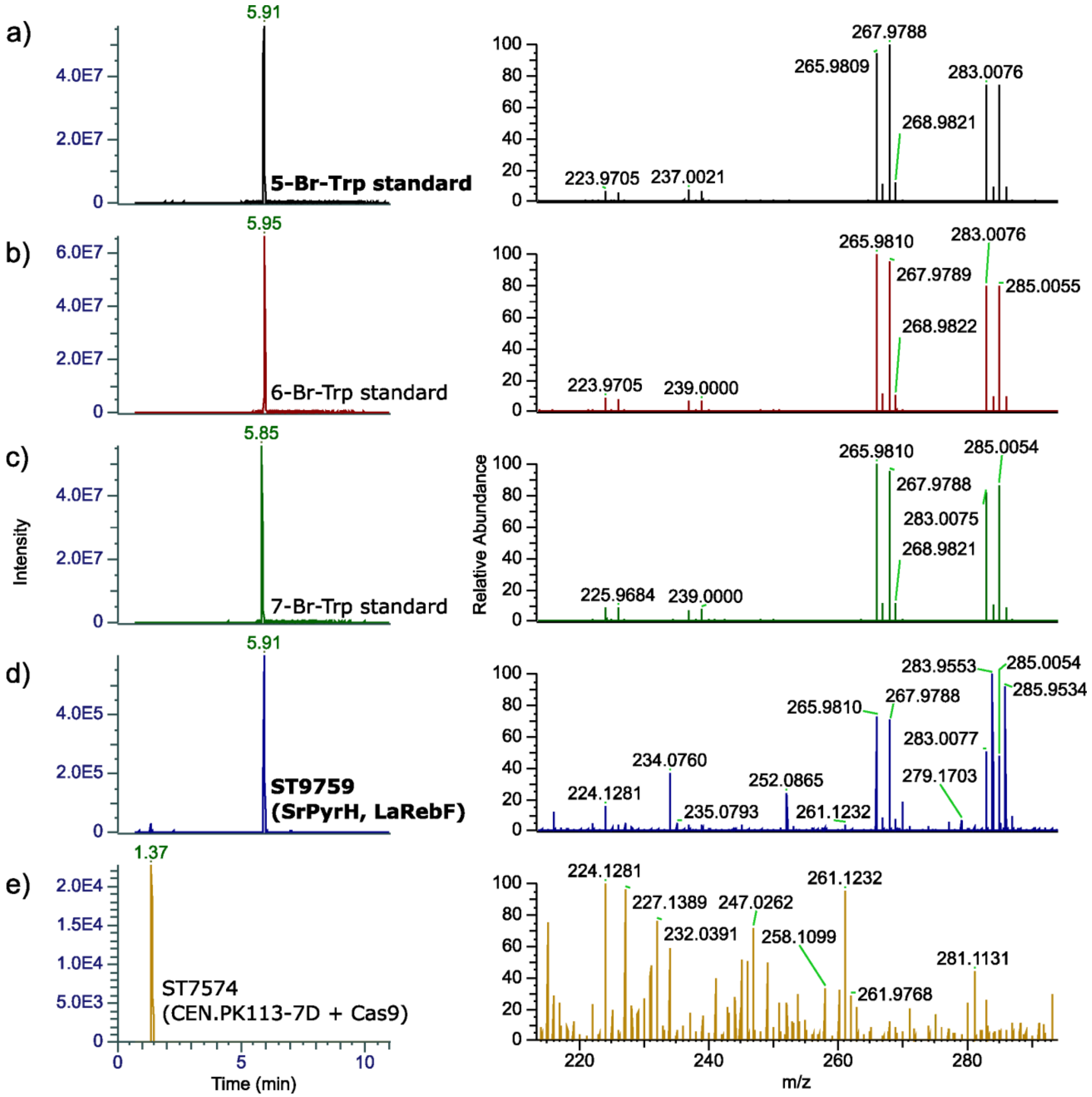

Figure S7. Production of 5-bromotryptophan in engineered *S. cerevisiae* strains. LC-MS extracted ion chromatograms and corresponding mass spectra of the main peak for a) 5-bromotryptophan standard, b) 6-bromotryptophan standard, c) 7-bromotryptophan standard, d) ST9759 (*SrPyrH*, *LaRebF*), e) ST7574 (Wild-type control, CEN.PK113-7D + Cas9). Theoretical m/z of $[M+H]^+$ and $[M+H-NH_3]^+$ adducts with ^{79}Br and ^{81}Br isotopes is 283.0077/265.9811 (most abundant) and 285.0056/267.9791, respectively. Note the presence of brominated xanthurenic acid as the main halogenated product, with observed $[M+H]^+$ m/z of 283.9553 (^{79}Br) and 285.9534 (^{81}Br).

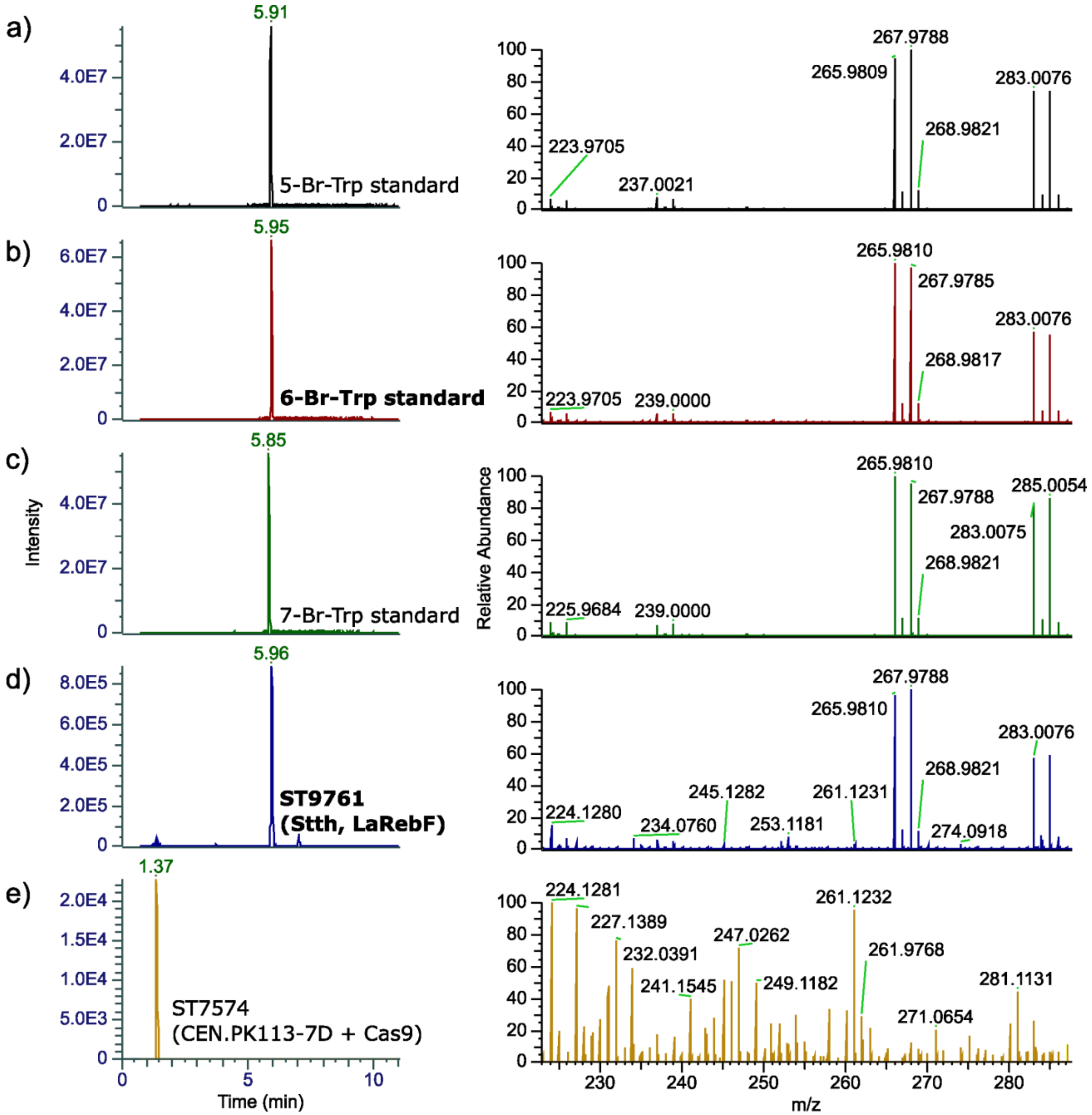

Figure S8. Production of 6-bromotryptophan in engineered *S. cerevisiae* strains. LC-MS extracted ion chromatograms and corresponding mass spectra of the main peak for a) 5-bromotryptophan standard, b) 6-bromotryptophan standard, c) 7-bromotryptophan standard, d) ST9761 (*SttH*, *LaRebF*), e) ST7574 (Wild-type control, CEN.PK113-7D + Cas9). Theoretical m/z of $[M+H]^+$ and $[M+H-NH_3]^+$ adducts with ^{79}Br and ^{81}Br isotopes is 283.0077/265.9811 (most abundant) and 285.0056/267.9791, respectively.

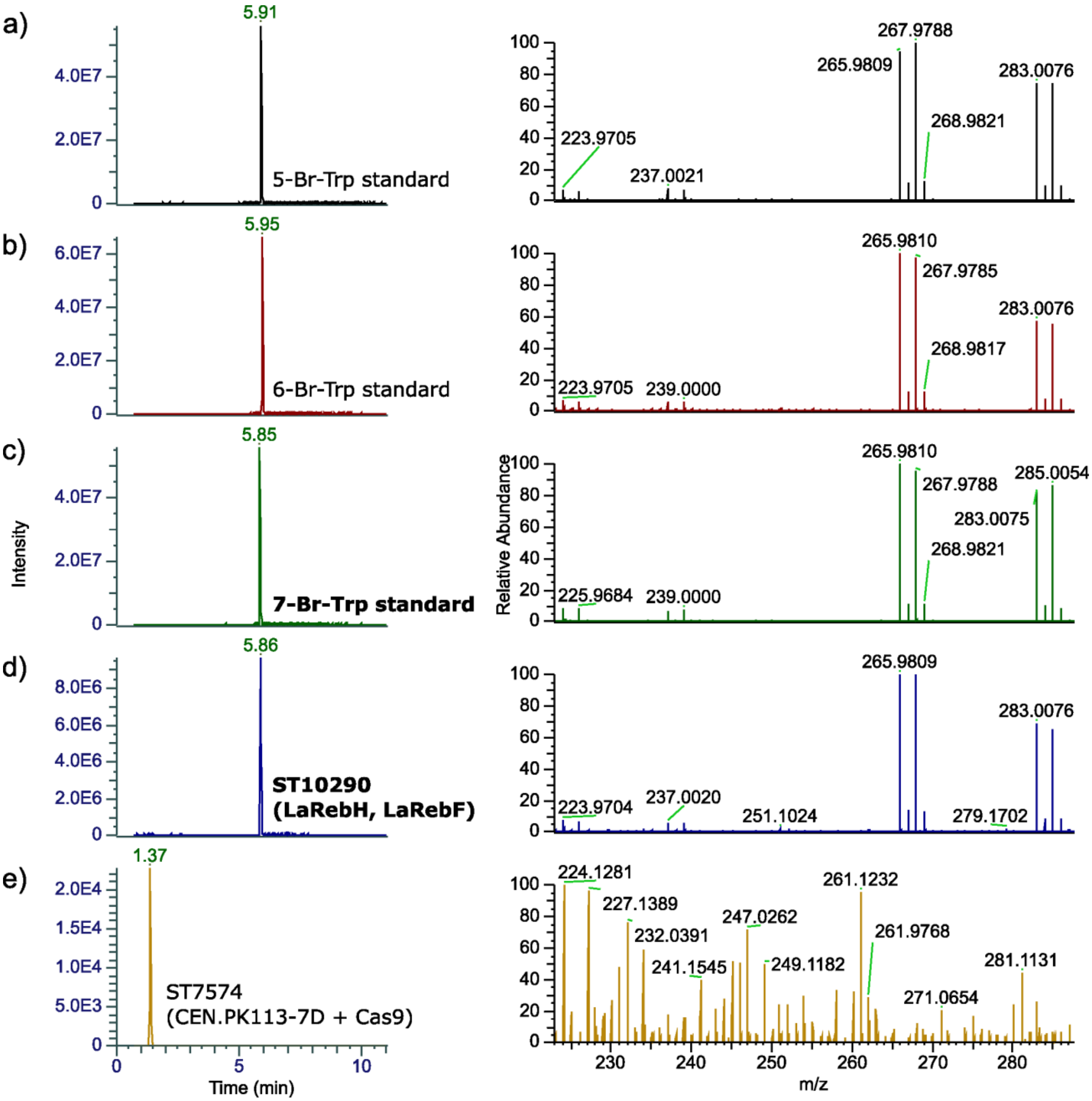

Figure S9. Production of 7-bromotryptophan in engineered *S. cerevisiae* strains. LC-MS extracted ion chromatograms and corresponding mass spectra of the main peak for a) 5-bromotryptophan standard, b) 6-bromotryptophan standard, c) 7-bromotryptophan standard, d) ST10290 (*LaRebH*, *LaRebF*), e) ST7574 (Wild-type control, CEN.PK113-7D + Cas9). Theoretical m/z of $[M+H]^+$ and $[M+H-NH_3]^+$ adducts with ^{79}Br and ^{81}Br isotopes is 283.0077/265.9811 (most abundant) and 285.0056/267.9791, respectively.

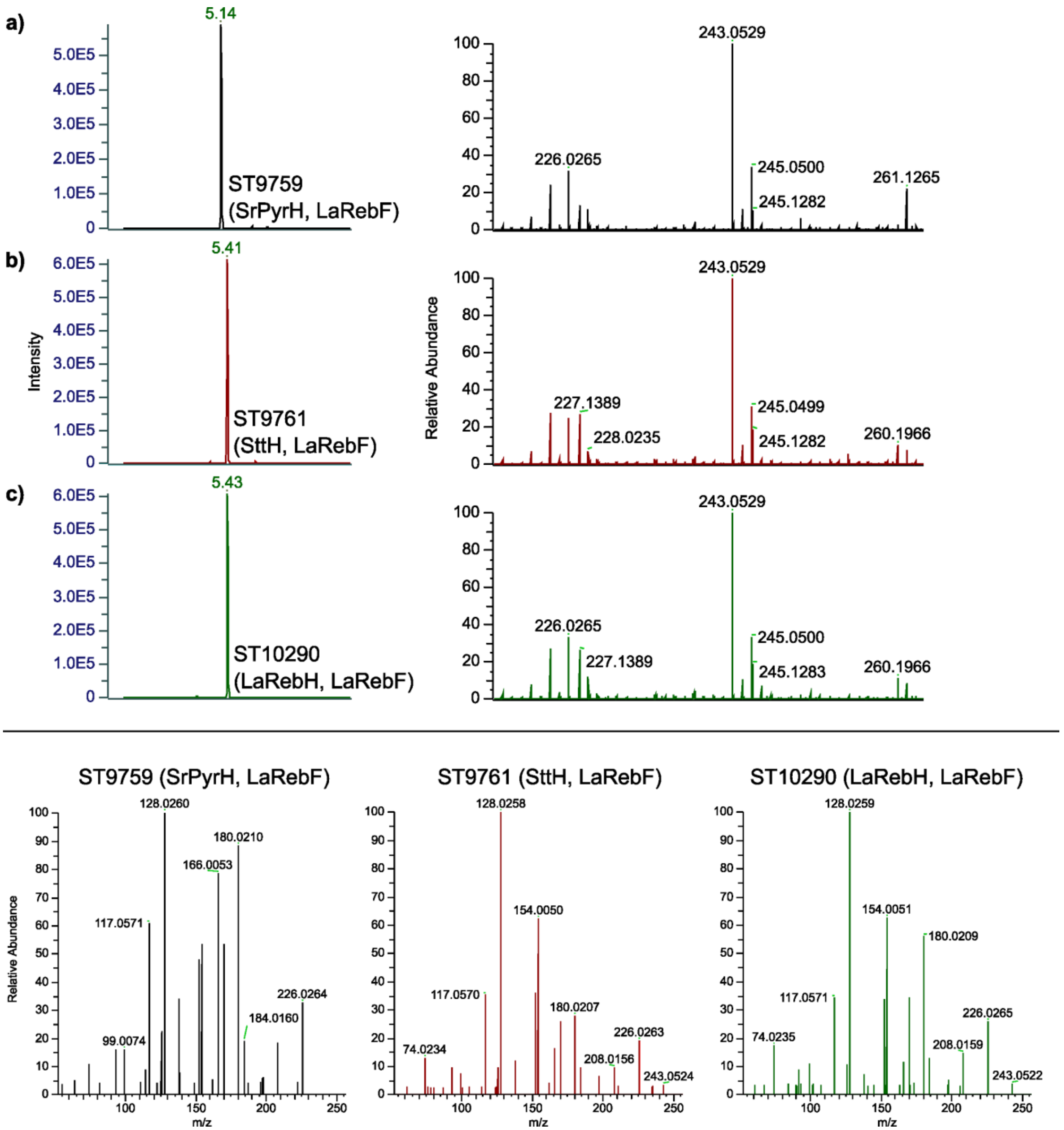

Figure S10. Production of chlorinated L-kynurenine in engineered *S. cerevisiae* strains. LC-MS extracted ion chromatograms and corresponding mass spectra of the main peak for a) ST9759 (*SrPyrH*, *LaRebF*), b) ST9761 (*SttH*, *LaRebF*), c) ST10290 (*LaRebH*, *LaRebF*). Theoretical m/z of $[M+H]^+$ adducts with ^{35}Cl and ^{37}Cl isotopes is 243.0537 (most abundant) and 245.0507, respectively. Bottom panel: MS^2 of precursor ion corresponding to $[M+H]^+$ adduct containing ^{35}Cl. n.d.: not detected or precursor ion not fragmented due to low intensity.

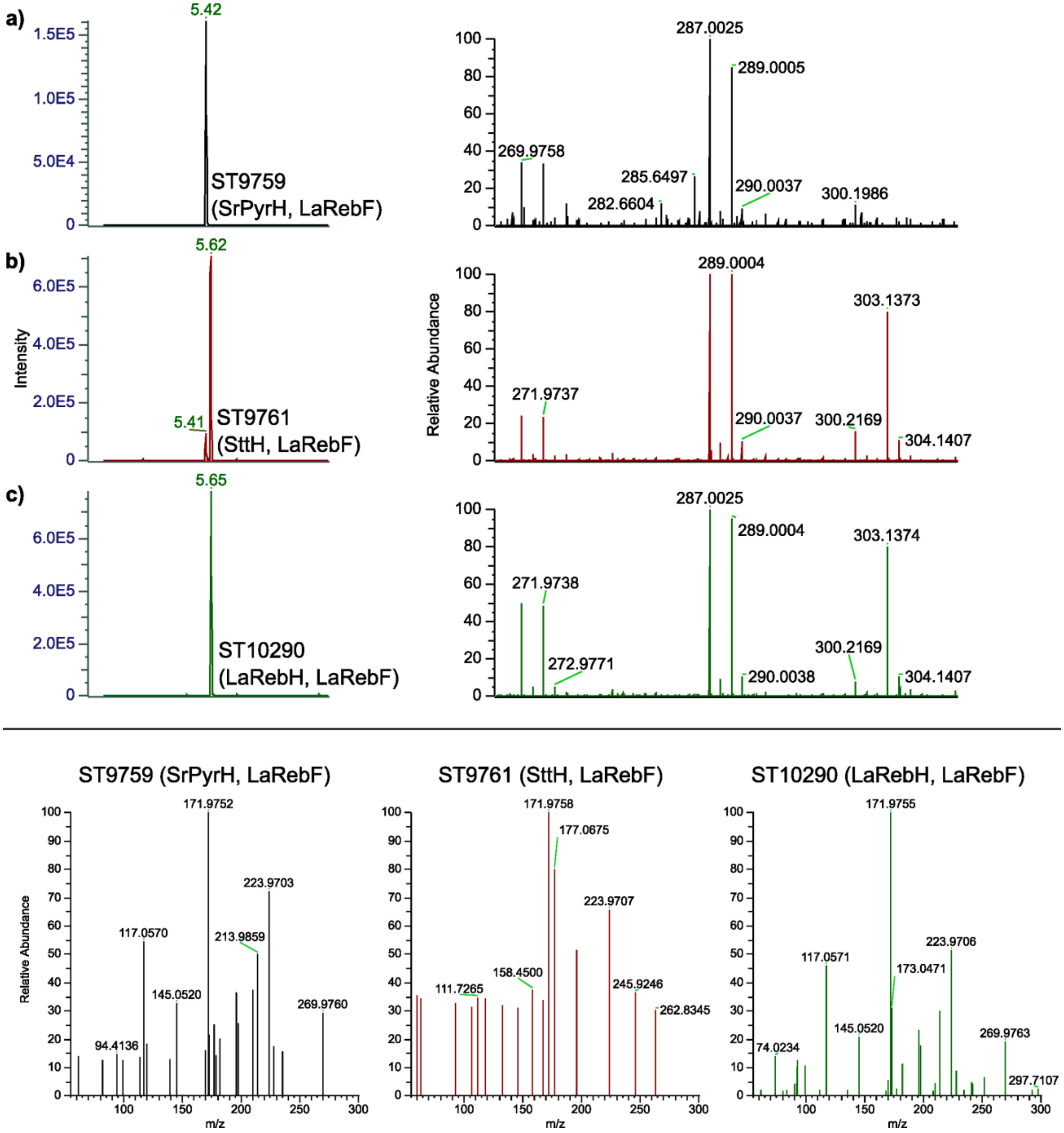

Figure S11. Production of brominated L-kynurenine in engineered *S. cerevisiae* strains. LC-MS extracted ion chromatograms and corresponding mass spectra of the main peak for a) ST9759 (*SrPyrH*, *LaRebF*), b) ST9761 (*SttH*, *LaRebF*), c) ST10290 (*LaRebH*, *LaRebF*). Theoretical m/z of $[M+H]^+$ adducts with ^{79}Br and ^{81}Br isotopes is 287.0031 (most abundant) and 289.0011, respectively. Bottom panel: MS^2 of precursor ion corresponding to $[M+H]^+$ adduct containing ^{79}Br. n.d.: not detected or precursor ion not fragmented due to low intensity.

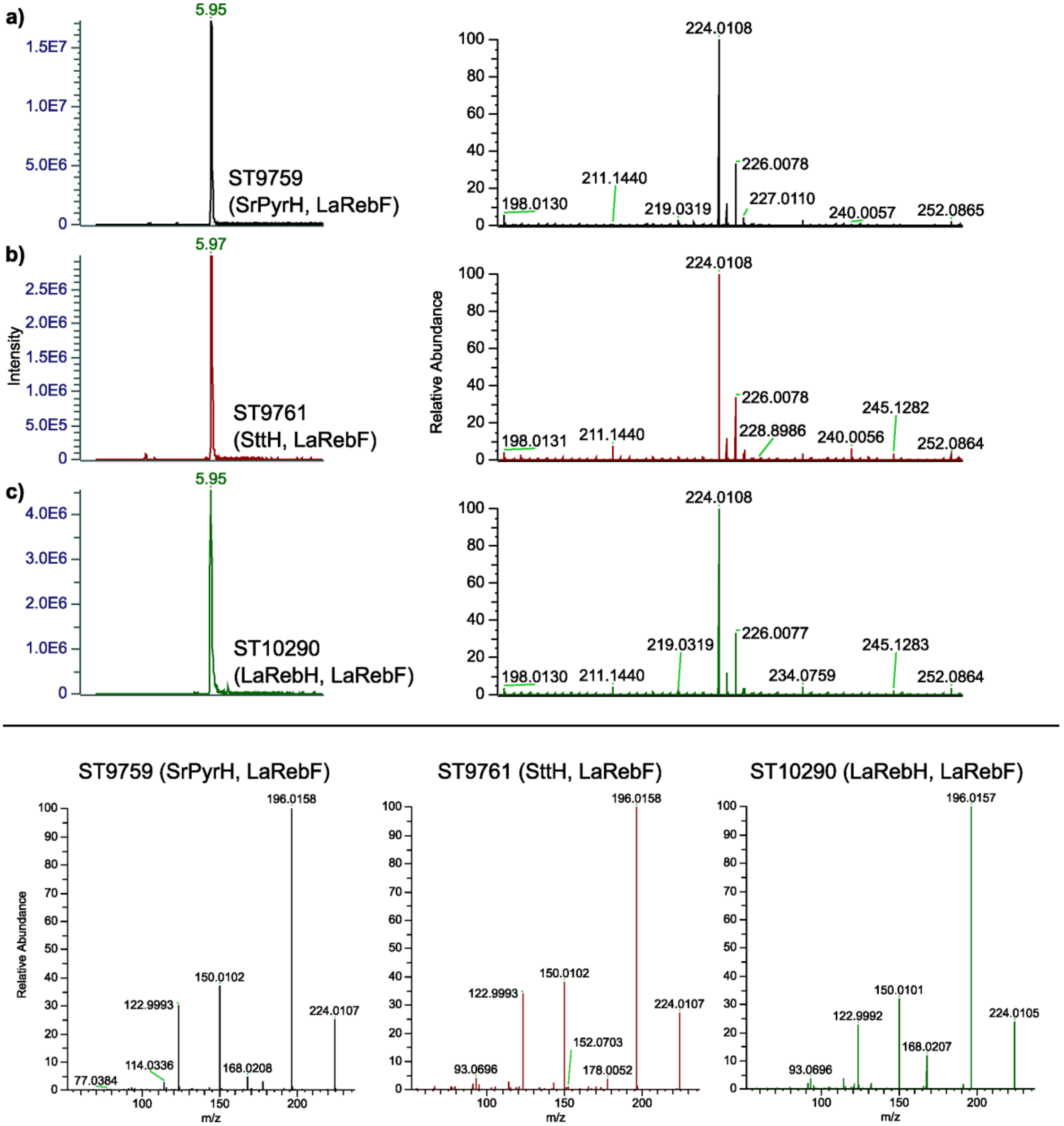

Figure S12. Production of chlorinated kynurenic acid in engineered *S. cerevisiae* strains. LC-MS extracted ion chromatograms and corresponding mass spectra of the main peak for a) ST9759 (*SrPyrH*, *LaRebF*), b) ST9761 (*SttH*, *LaRebF*), c) ST10290 (*LaRebH*, *LaRebF*). Theoretical m/z of $[M+H]^+$ adducts with ^{35}Cl and ^{37}Cl isotopes is 224.0115 (most abundant) and 226.0085, respectively. Bottom panel: MS^2 of precursor ion corresponding to $[M+H]^+$ adduct containing ^{35}Cl. n.d.: not detected or precursor ion not fragmented due to low intensity.

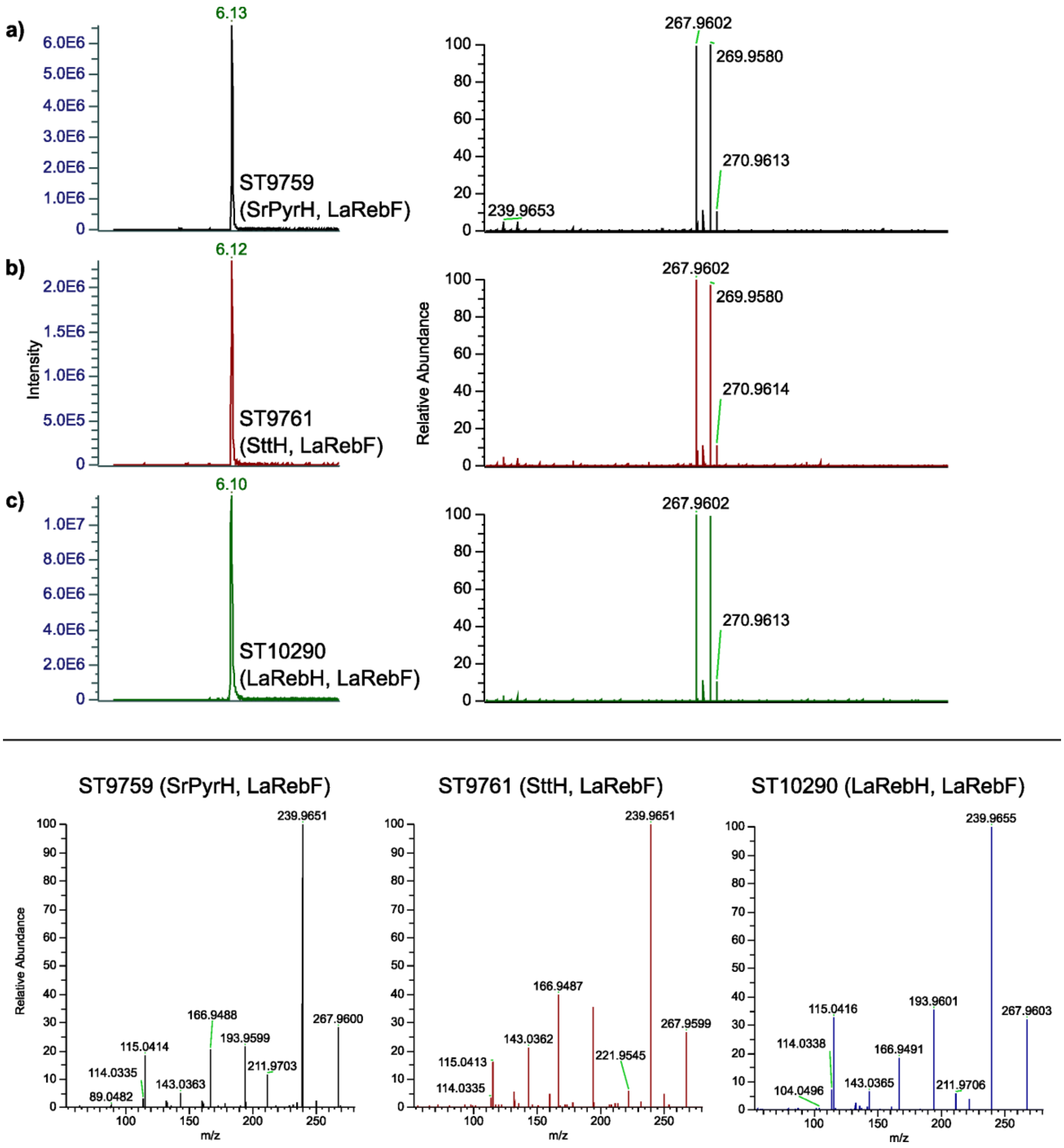

Figure S13. Production of brominated kynurenic acid in engineered *S. cerevisiae* strains. LC-MS extracted ion chromatograms and corresponding mass spectra of the main peak for a) ST9759 (*SrPyrH*, *LaRebF*), b) ST9761 (*SttH*, *LaRebF*), c) ST10290 (*LaRebH*, *LaRebF*). Theoretical m/z of $[M+H]^+$ adducts with ^{79}Br and ^{81}Br isotopes is 267.9609 (most abundant) and 269.9589, respectively. Bottom panel: MS^2 of precursor ion corresponding to $[M+H]^+$ adduct containing ^{79}Br. n.d.: not detected or precursor ion not fragmented due to low intensity.

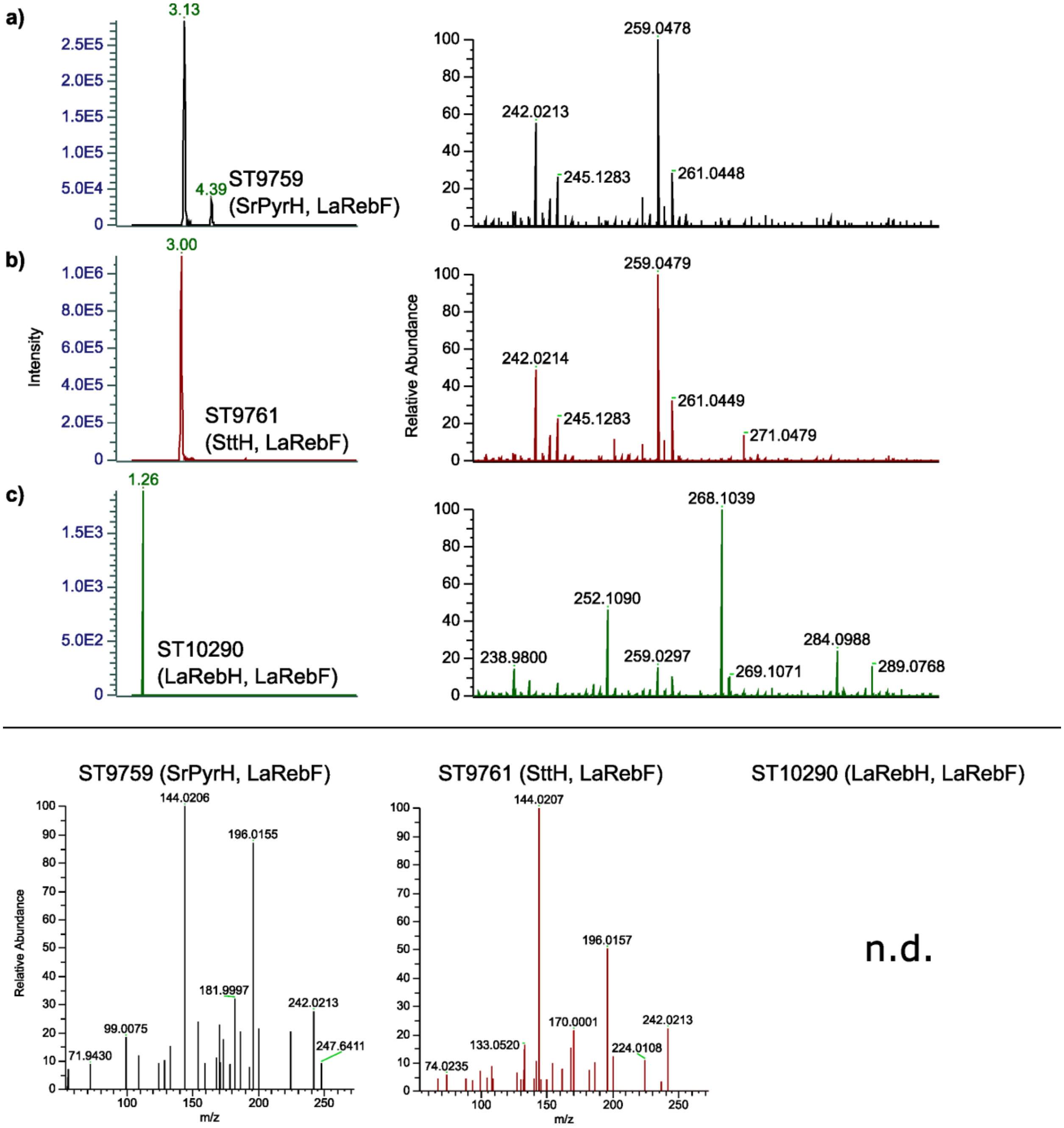

Figure S14. Production of chlorinated 3-hydroxy-L-kynurenine in engineered *S. cerevisiae* strains. LC-MS extracted ion chromatograms and corresponding mass spectra of the main peak for a) ST9759 (*SrPyrH*, *LaRebF*), b) ST9761 (*SttH*, *LaRebF*), c) ST10290 (*LaRebH*, *LaRebF*), compound not detected. Theoretical m/z of [M+H]$^+$ adducts with ^{35}Cl and ^{37}Cl isotopes is 259.0486 (most abundant) and 261.0456, respectively. Bottom panel: MS^2 of precursor ion corresponding to [M+H]$^+$ adduct containing ^{35}Cl. n.d.: not detected or precursor ion not fragmented due to low intensity.

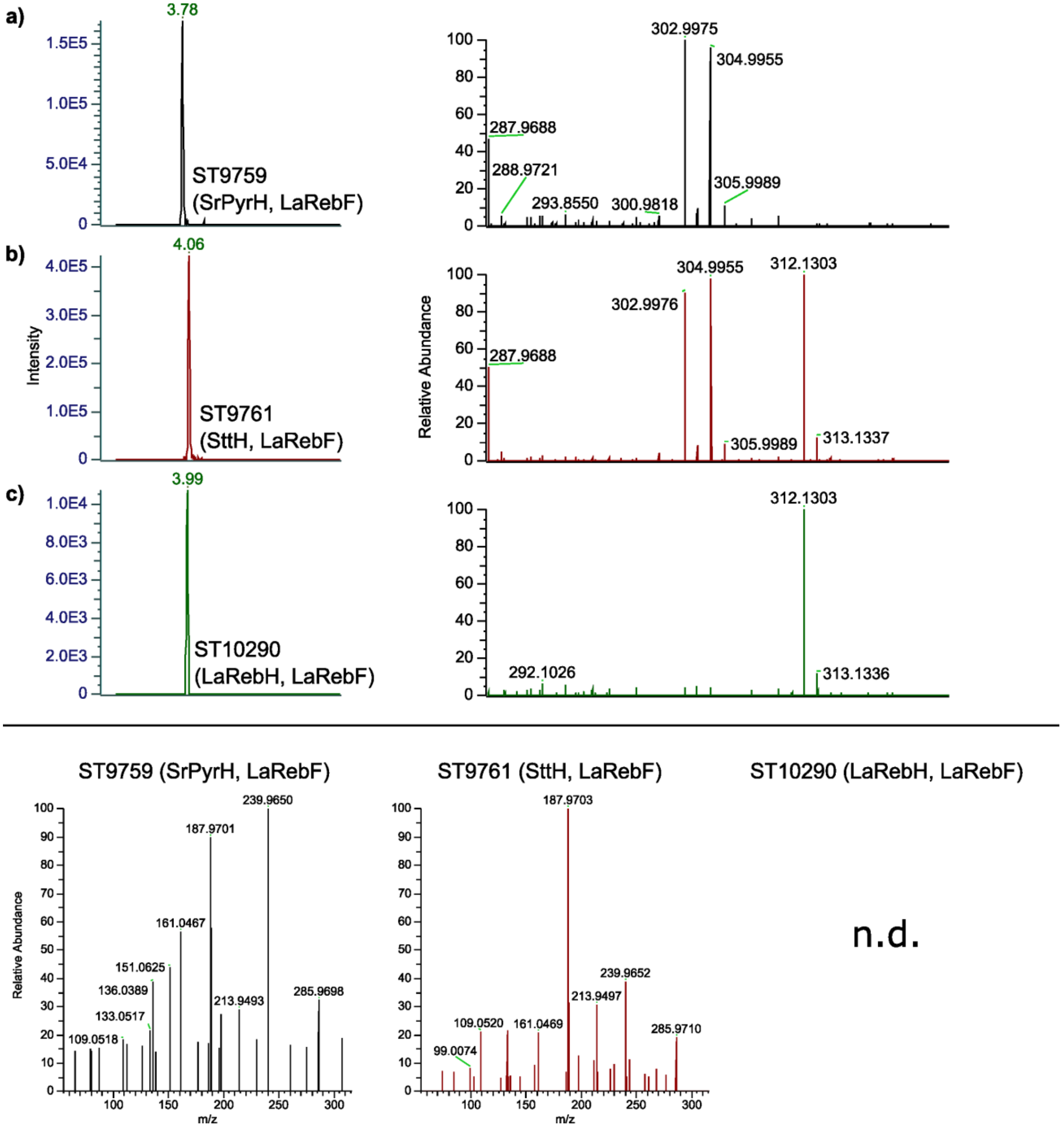

Figure S15. Production of brominated 3-hydroxy-L-kynurenine in engineered *S. cerevisiae* strains. LC-MS extracted ion chromatograms and corresponding mass spectra of the main peak for a) ST9759 (*SrPyrH*, *LaRebF*), b) ST9761 (*SttH*, *LaRebF*), c) ST10290 (*LaRebH*, *LaRebF*)), compound not detected. Theoretical m/z of $[M+H]^+$ adducts with ^{79}Br and ^{81}Br isotopes is 302.9980 (most abundant) and 304.9960, respectively. Bottom panel: MS^2 of precursor ion corresponding to $[M+H]^+$ adduct containing ^{79}Br. n.d.: not detected or precursor ion not fragmented due to low intensity.

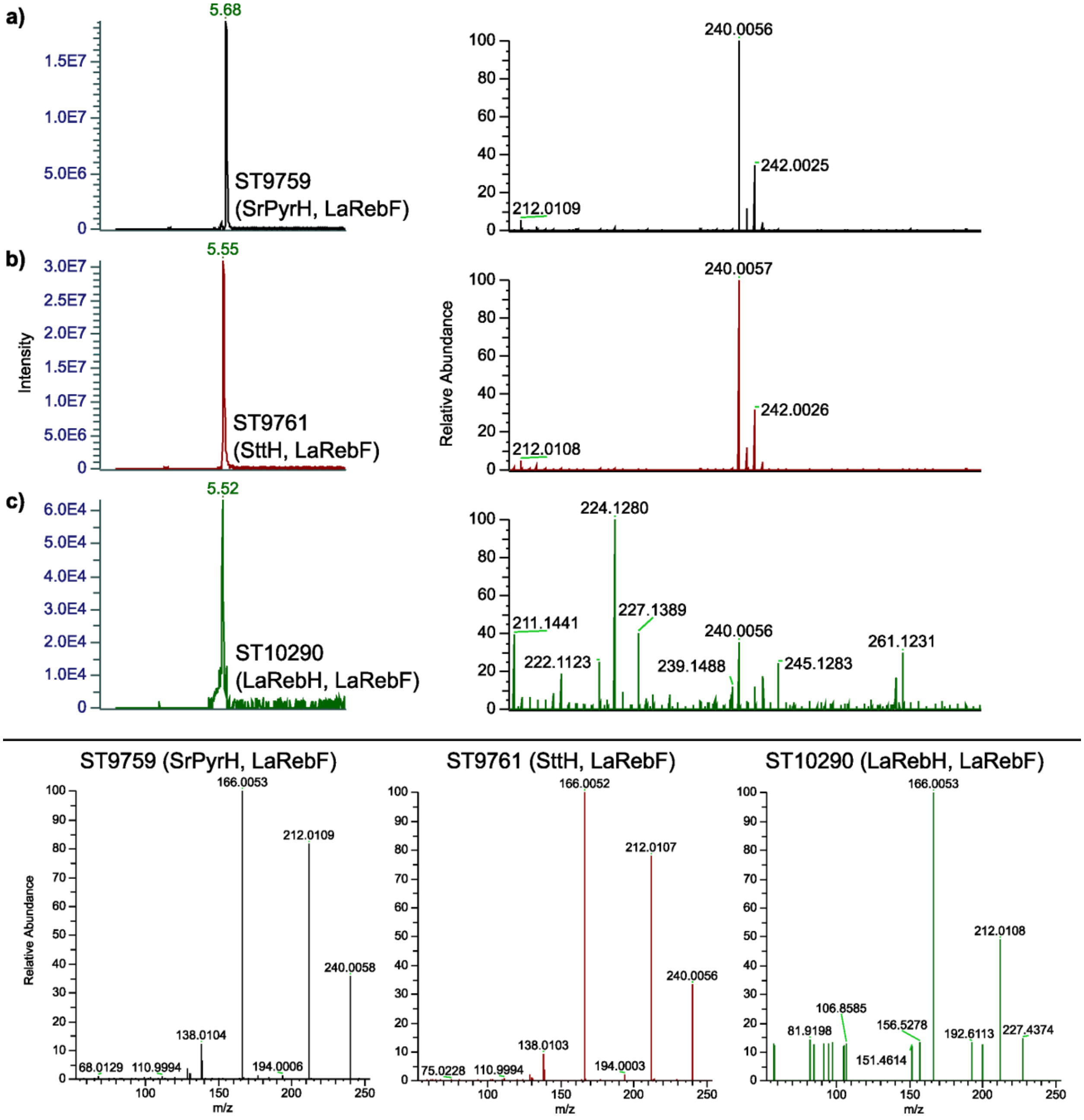

Figure S16. Production of chlorinated xanthurenic acid in engineered *S. cerevisiae* strains. LC-MS extracted ion chromatograms and corresponding mass spectra of the main peak for a) ST9759 (*SrPyrH*, *LaRebF*), b) ST9761 (*SttH*, *LaRebF*), c) ST10290 (*LaRebH*, *LaRebF*). Theoretical m/z of $[M+H]^+$ adducts with ^{35}Cl and ^{37}Cl isotopes is 240.0064 (most abundant) and 242.0034, respectively. Bottom panel: MS^2 of precursor ion corresponding to $[M+H]^+$ adduct containing ^{35}Cl. n.d.: not detected or precursor ion not fragmented due to low intensity.

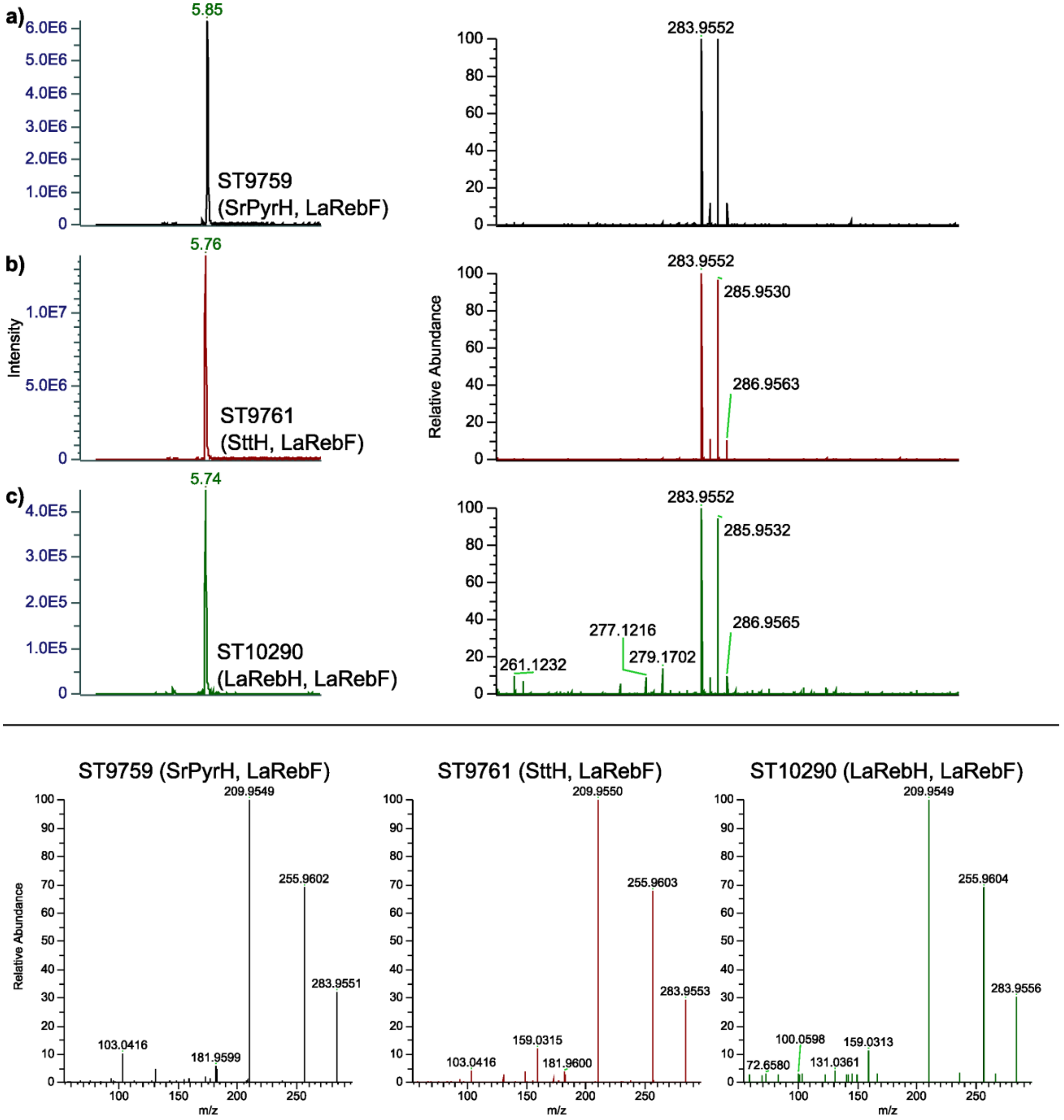

Figure S17. Production of brominated xanthurenic acid in engineered *S. cerevisiae* strains. LC-MS extracted ion chromatograms and corresponding mass spectra of the main peak for a) ST9759 (*SrPyrH*, *LaRebF*), b) ST9761 (*SttH*, *LaRebF*), c) ST10290 (*LaRebH*, *LaRebF*). Theoretical m/z of $[M+H]^+$ adducts with ^{79}Br and ^{81}Br isotopes is 283.9558 (most abundant) and 285.9538, respectively. Bottom panel: MS^2 of precursor ion corresponding to $[M+H]^+$ adduct containing ^{79}Br. n.d.: not detected or precursor ion not fragmented due to low intensity.

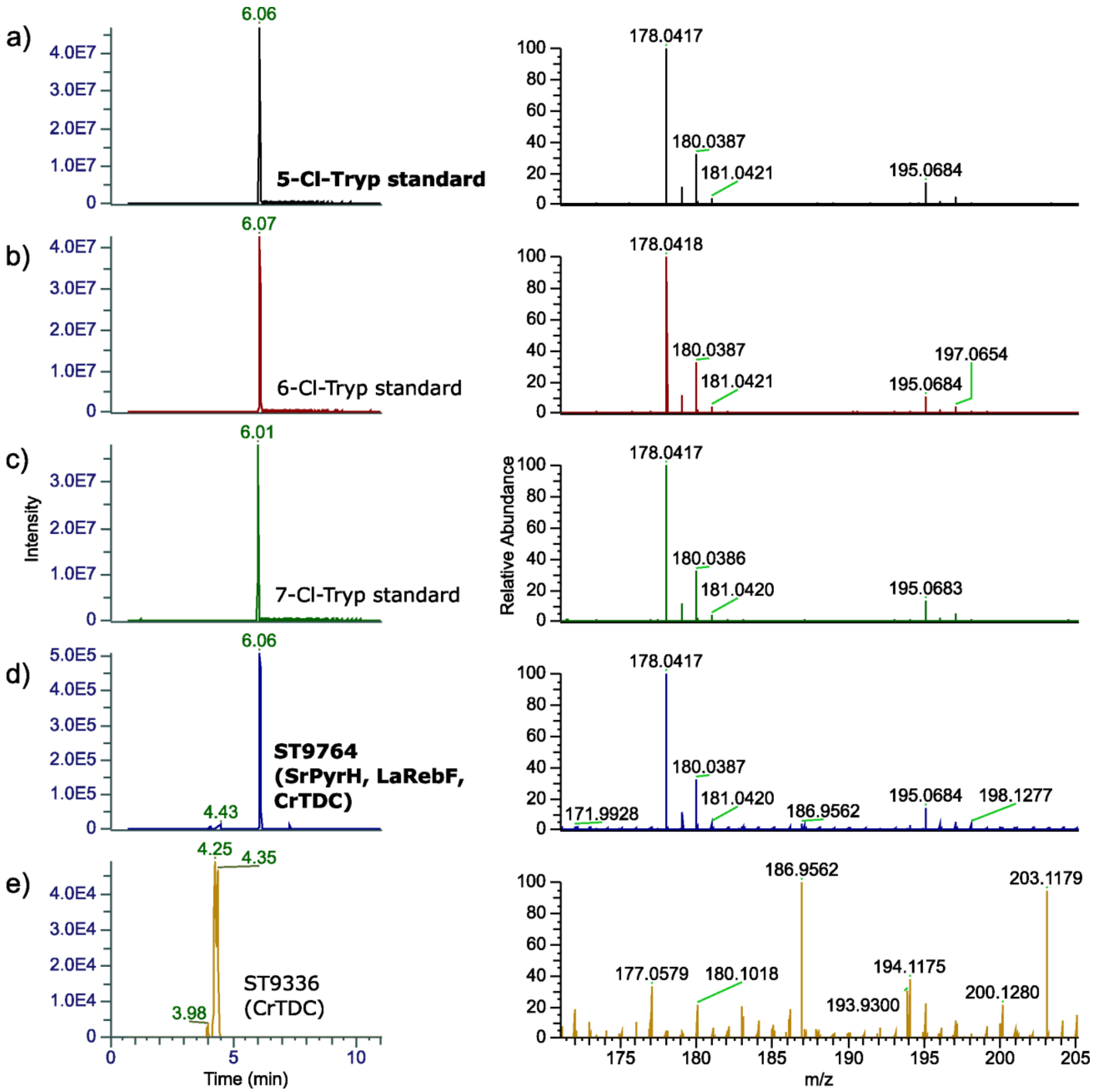

Figure S18. Production of 5-chlorotryptamine in engineered *S. cerevisiae* strains. LC-MS extracted ion chromatograms and corresponding mass spectra of the main peak for a) 5-chlorotryptamine standard, b) 6-chlorotryptamine standard, c) 7-chlorotryptamine standard, d) ST9764 (*SrPyrH*, *LaRebF*, *CrTDC*), e) ST9336 (Tryptamine control, *CrTDC*). Theoretical m/z of $[M+H]^+$ and $[M+H-NH_3]^+$ adducts with ^{35}Cl and ^{37}Cl isotopes is 195.0684/178.0418 (most abundant) and 197.0654/180.0389, respectively.

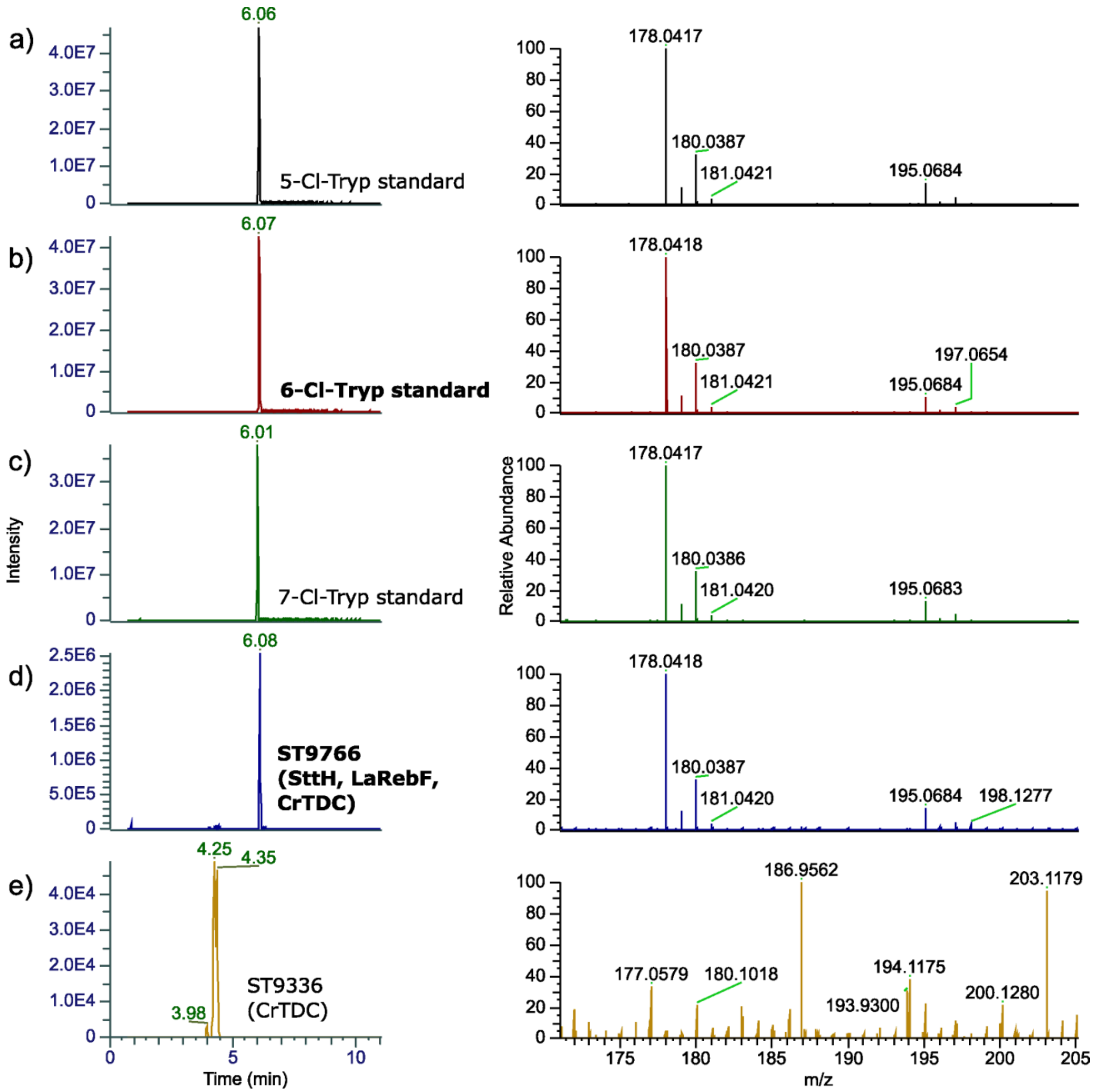

Figure S19. Production of 6-chlorotryptamine in engineered *S. cerevisiae* strains. LC-MS extracted ion chromatograms and corresponding mass spectra of the main peak for a) 5-chlorotryptamine standard, b) 6-chlorotryptamine standard, c) 7-chlorotryptamine standard, d) ST9766 (*SttH*, *LaRebF*, *CrTDC*), e) ST9336 (Tryptamine control, *CrTDC*). Theoretical m/z of $[M+H]^+$ and $[M+H-NH_3]^+$ adducts with ^{35}Cl and ^{37}Cl isotopes is 195.0684/178.0418 (most abundant) and 197.0654/180.0389, respectively.

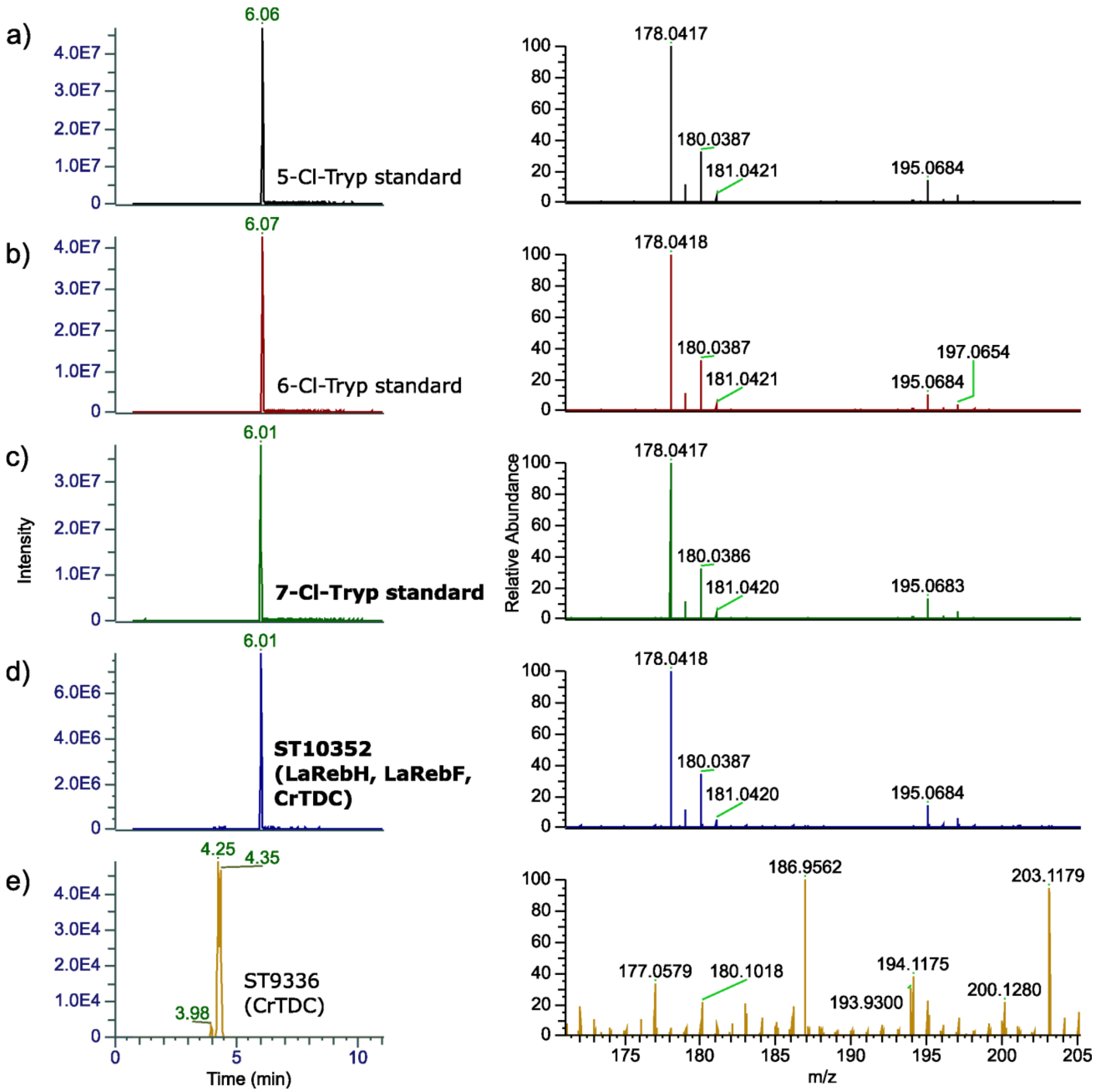

Figure S20. Production of 7-chlorotryptamine in engineered *S. cerevisiae* strains. LC-MS extracted ion chromatograms and corresponding mass spectra of the main peak for a) 5-chlorotryptamine standard, b) 6-chlorotryptamine standard, c) 7-chlorotryptamine standard, d) ST10352 (*LaRebH*, *LaRebF*, *CrTDC*), e) ST9336 (Tryptamine control, *CrTDC*). Theoretical m/z of $[M+H]^+$ and $[M+H-NH_3]^+$ adducts with ^{35}Cl and ^{37}Cl isotopes is 195.0684/178.0418 (most abundant) and 197.0654/180.0389, respectively.

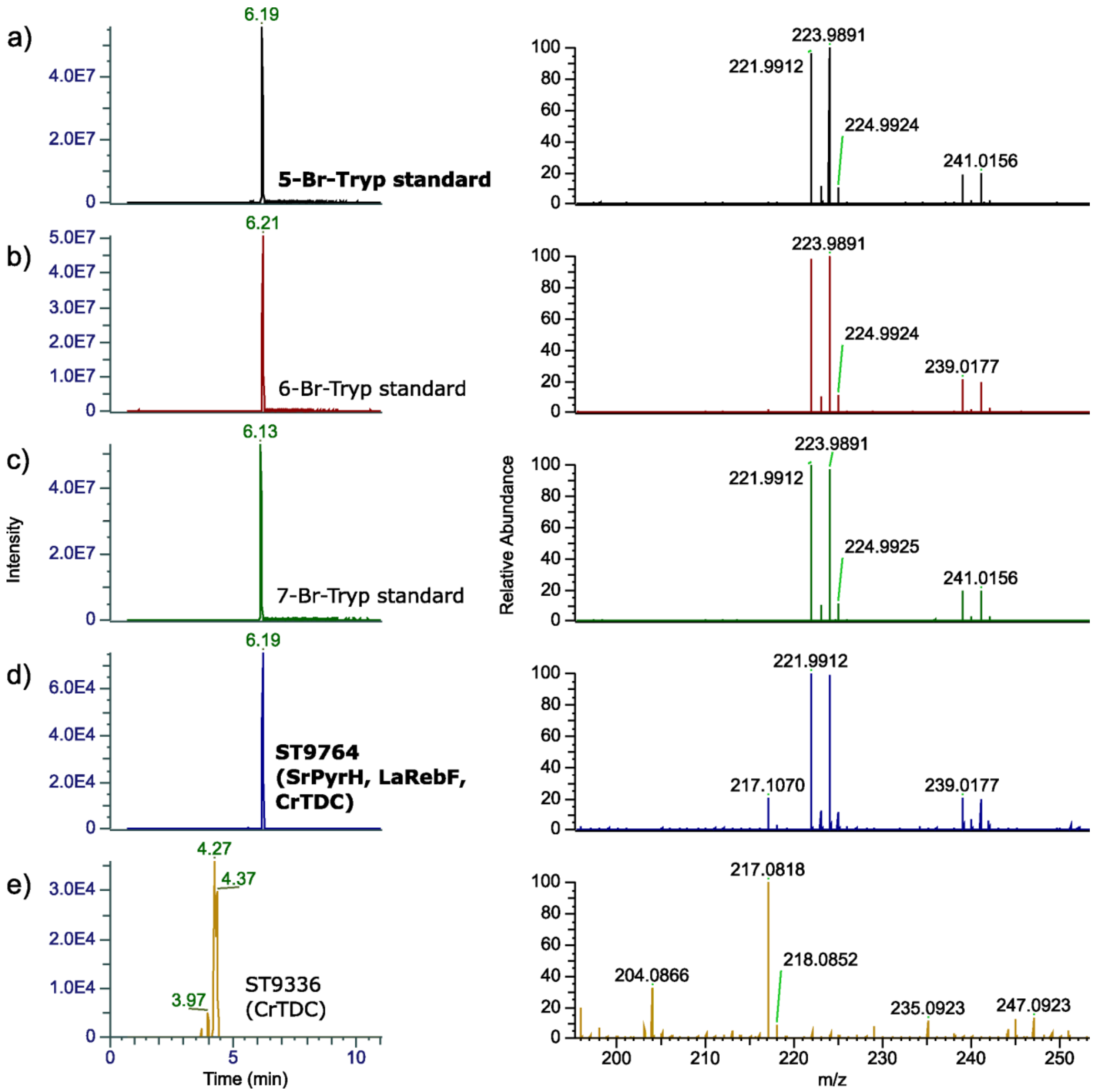

Figure S21. Production of 5-bromotryptamine in engineered *S. cerevisiae* strains. LC-MS extracted ion chromatograms and corresponding mass spectra of the main peak for a) 5-bromotryptamine standard, b) 6-bromotryptamine standard, c) 7-bromotryptamine standard, d) ST9764 (*SrPyrH*, *LaRebF*, *CrTDC*), e) ST9336 (Tryptamine control, *CrTDC*). Theoretical m/z of $[M+H]^+$ and $[M+H-NH_3]^+$ adducts with ^{79}Br and ^{81}Br isotopes is 239.0178/221.9913 (most abundant) and 241.0158/223.9832, respectively.

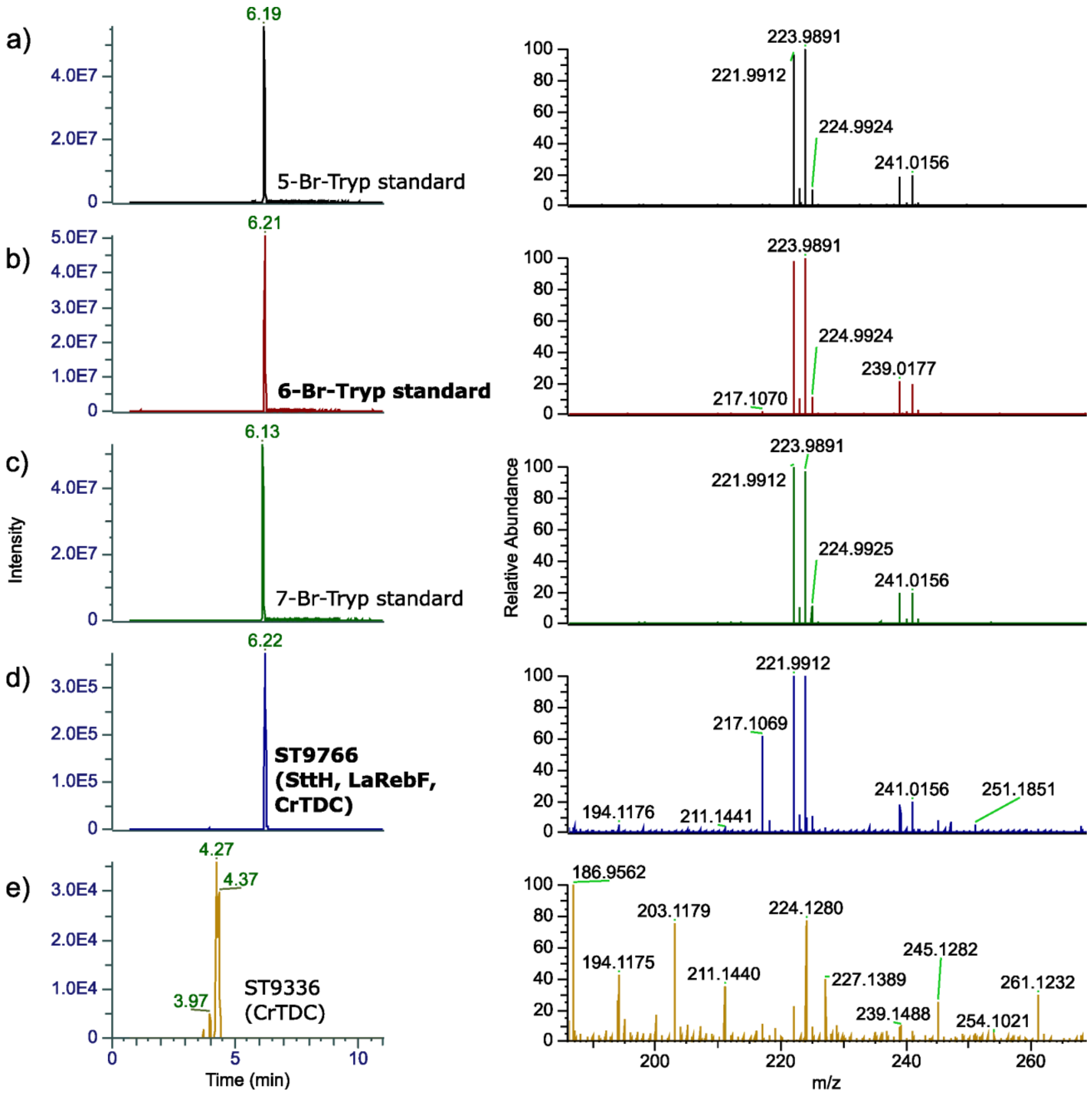

Figure S22. Production of 6-bromotryptamine in engineered *S. cerevisiae* strains. LC-MS extracted ion chromatograms and corresponding mass spectra of the main peak for a) 5-bromotryptamine standard, b) 6-bromotryptamine standard, c) 7-bromotryptamine standard, d) ST9766 (*SttH*, *LaRebF*, *CrTDC*), e) ST9336 (Tryptamine control, *CrTDC*). Theoretical m/z of $[M+H]^+$ and $[M+H-NH_3]^+$ adducts with ^{79}Br and ^{81}Br isotopes is 239.0178/221.9913 (most abundant) and 241.0158/223.9832, respectively.

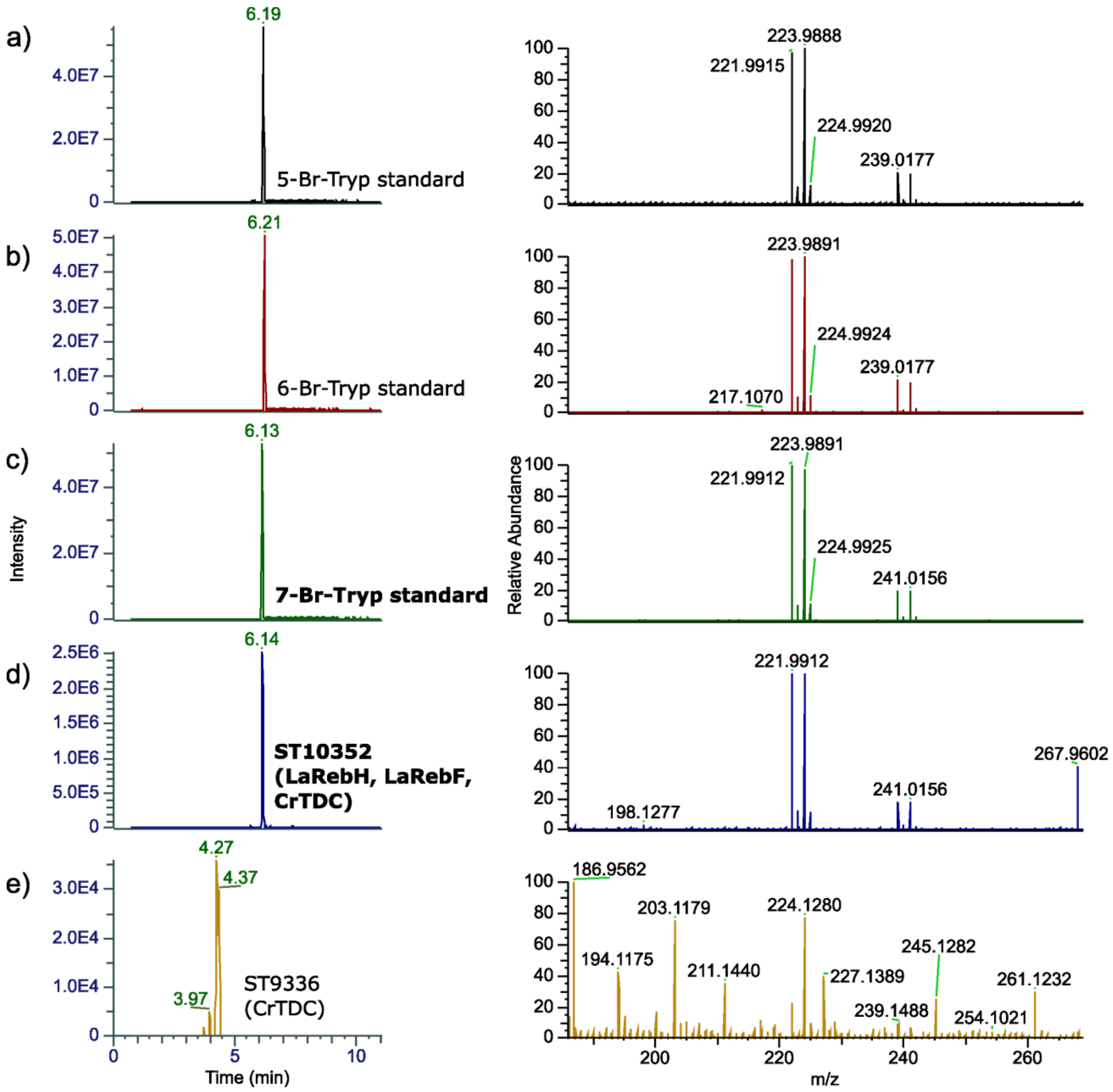

Figure S23. Production of 7-bromotryptamine in engineered *S. cerevisiae* strains. LC-MS extracted ion chromatograms and corresponding mass spectra of the main peak for a) 5-bromotryptamine standard, b) 6-bromotryptamine standard, c) 7-bromotryptamine standard, d) ST10352 (*LaRebH*, *LaRebF*, *CrTDC*), e) ST9336 (Tryptamine control, *CrTDC*). Theoretical m/z of $[M+H]^+$ and $[M+H-NH_3]^+$ adducts with ^{79}Br and ^{81}Br isotopes is 239.0178/221.9913 (most abundant) and 241.0158/223.9832, respectively.

a

Strain	*SrPyrH*	*SttH*	*LaRebH*	*LaRebF*	*CrTDC*
ST7574	−	−	−	−	−
ST9759	+	−	−	+	−
ST9760	+	−	−	−	−
ST9761	−	+	−	+	−
ST9762	−	+	−	−	−
ST10290	−	−	+	+	−
ST10291	−	−	+	−	−
ST9336	−	−	−	−	+
ST9764	+	−	−	+	+
ST9765	+	−	−	−	+
ST9766	−	+	−	+	+
ST9767	−	+	−	−	+
ST10352	−	−	+	+	+
ST10071	−	−	+	−	+

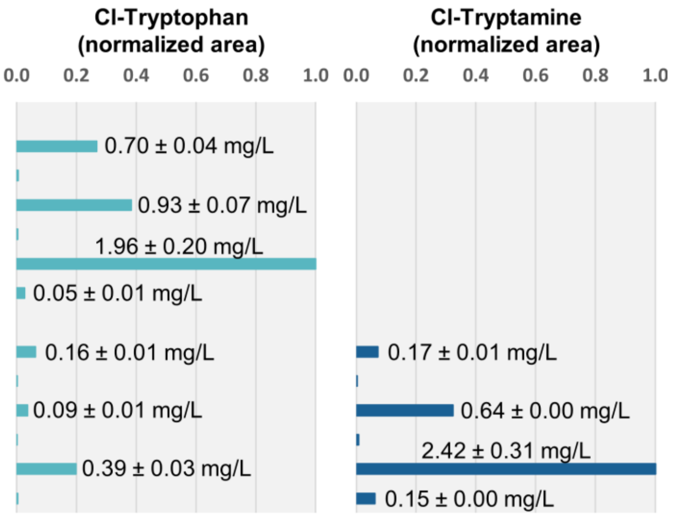

b

Strain	*SrPyrH*	*SttH*	*LaRebH*	*LaRebF*	*CrTDC*
ST7574	−	−	−	−	−
ST9759	+	−	−	+	−
ST9760	+	−	−	−	−
ST9761	−	+	−	+	−
ST9762	−	+	−	−	−
ST10290	−	−	+	+	−
ST10291	−	−	+	−	−
ST9336	−	−	−	−	+
ST9764	+	−	−	+	+
ST9765	+	−	−	−	+
ST9766	−	+	−	+	+
ST9767	−	+	−	−	+
ST10352	−	−	+	+	+
ST10071	−	−	+	−	+

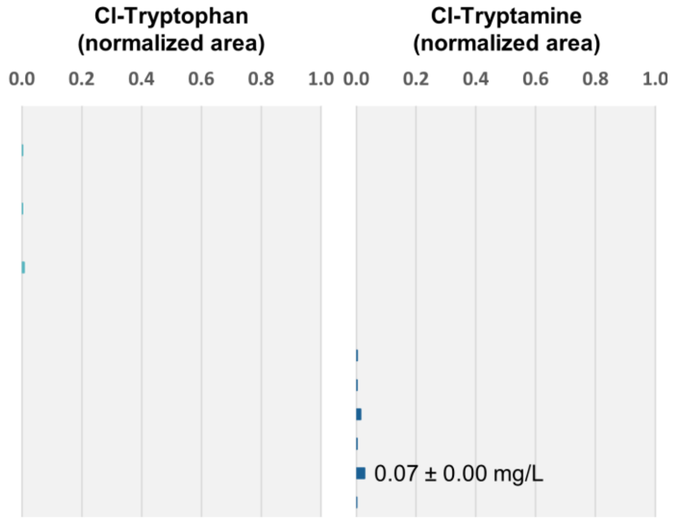

Figure S24. Production of chlorinated tryptophan and tryptamine in recombinant *S. cerevisiae* strains. Yeast strains were cultivated for 72 hours in synthetic mineral medium supplemented with a) 25 mᴍ KCl or b) 25 mᴍ KBr. "+" and "−" symbols indicate the presence or absence of the corresponding genetic modification, respectively. Cultivation broths of strains lacking *CrTDC* were subjected to the intracellular extraction protocol. Cultivation broths of strains expressing CrTDC were centrifuged and the supernatants were used for the analysis. Titers of halogenated products are reported as normalized peak areas, meaning that areas matching the retention time, expected m/z, and fragmentation pattern of the metabolite of interest have been normalized with respect to the highest-producing strain of that metabolite. Error values represent the standard deviation from two biological replicates. Data available in the Supplementary File 1.

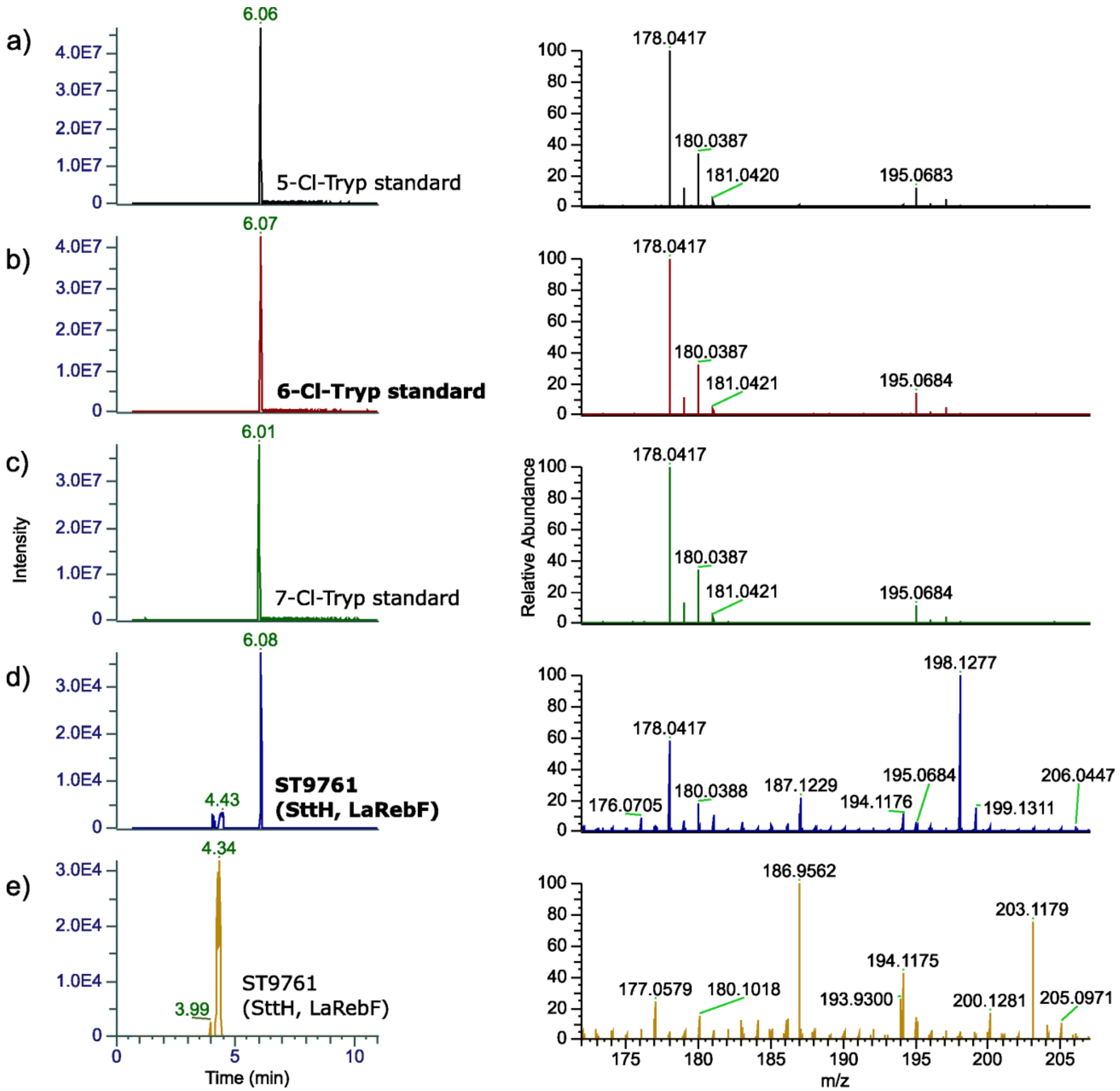

Figure S25. Direct chlorination of tryptamine in engineered *S. cerevisiae* strain expressing *SttH*. LC-MS extracted ion chromatograms and corresponding mass spectra of the main peak for a) 5-chlorotryptamine standard, b) 6-chlorotryptamine standard, c) 7-chlorotryptamine standard, d) ST9761 (*SttH*, *LaRebF*) fed with 1 mM tryptamine, e) ST9761 (*SttH*, *LaRebF*) without tryptamine feeding. Theoretical m/z of $[M+H]^+$ and $[M+H-NH_3]^+$ adducts with ^{35}Cl and ^{37}Cl isotopes is 195.0684/178.0418 (most abundant) and 197.0654/180.0389, respectively.

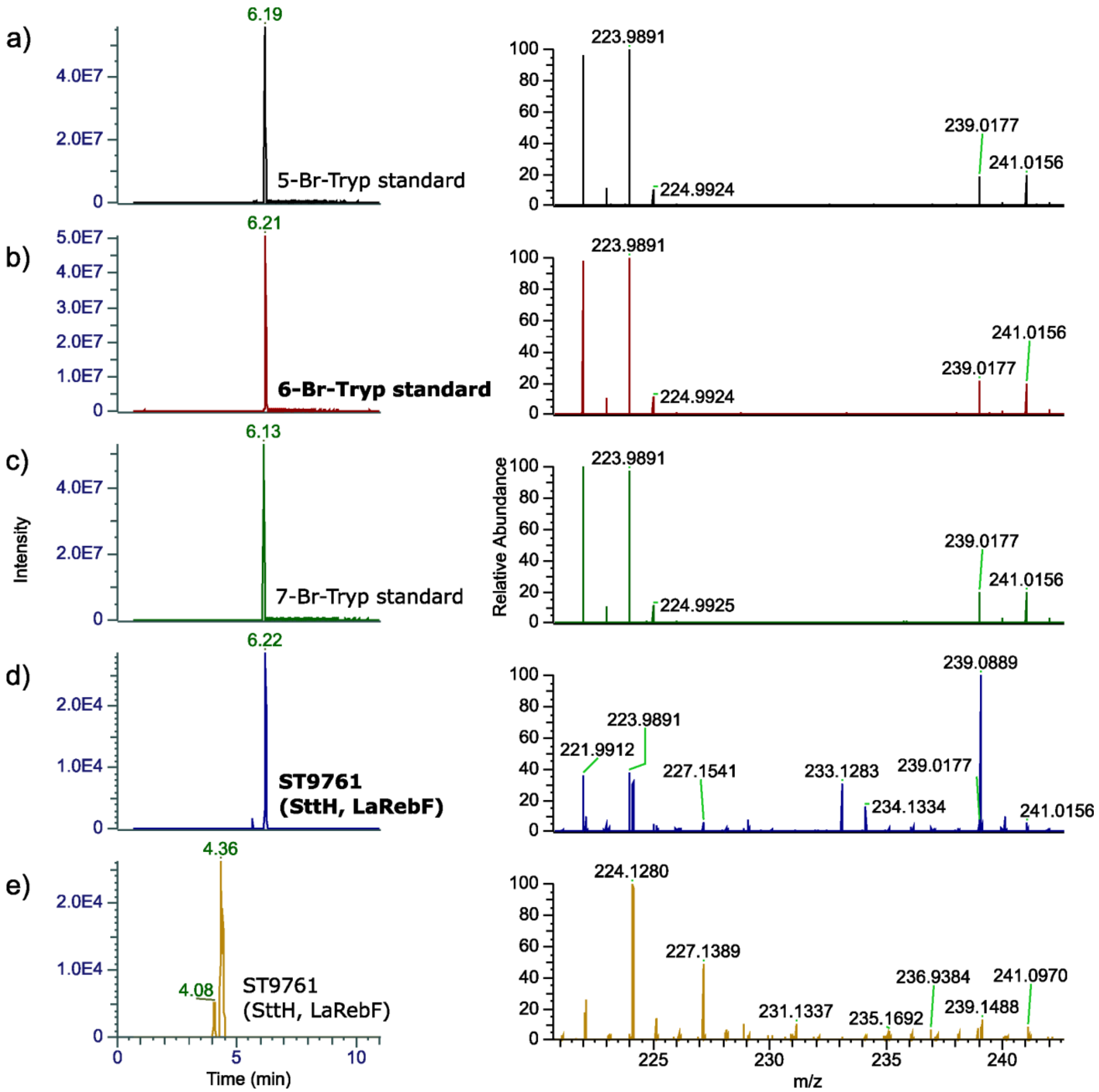

Figure S26. Direct bromination of tryptamine in engineered *S. cerevisiae* strain expressing *SttH*. LC-MS extracted ion chromatograms and corresponding mass spectra of the main peak for a) 5-bromotryptamine standard, b) 6-bromotryptamine standard, c) 7-bromotryptamine standard, ST9761 (*SttH*, *LaRebF*) fed with 1 mM tryptamine, e) ST9761 (*SttH*, *LaRebF*) without tryptamine feeding. Theoretical m/z of $[M+H]^+$ and $[M+H-NH_3]^+$ adducts with ^{79}Br and ^{81}Br isotopes is 239.0178/221.9913 (most abundant) and 241.0158/223.9832, respectively.

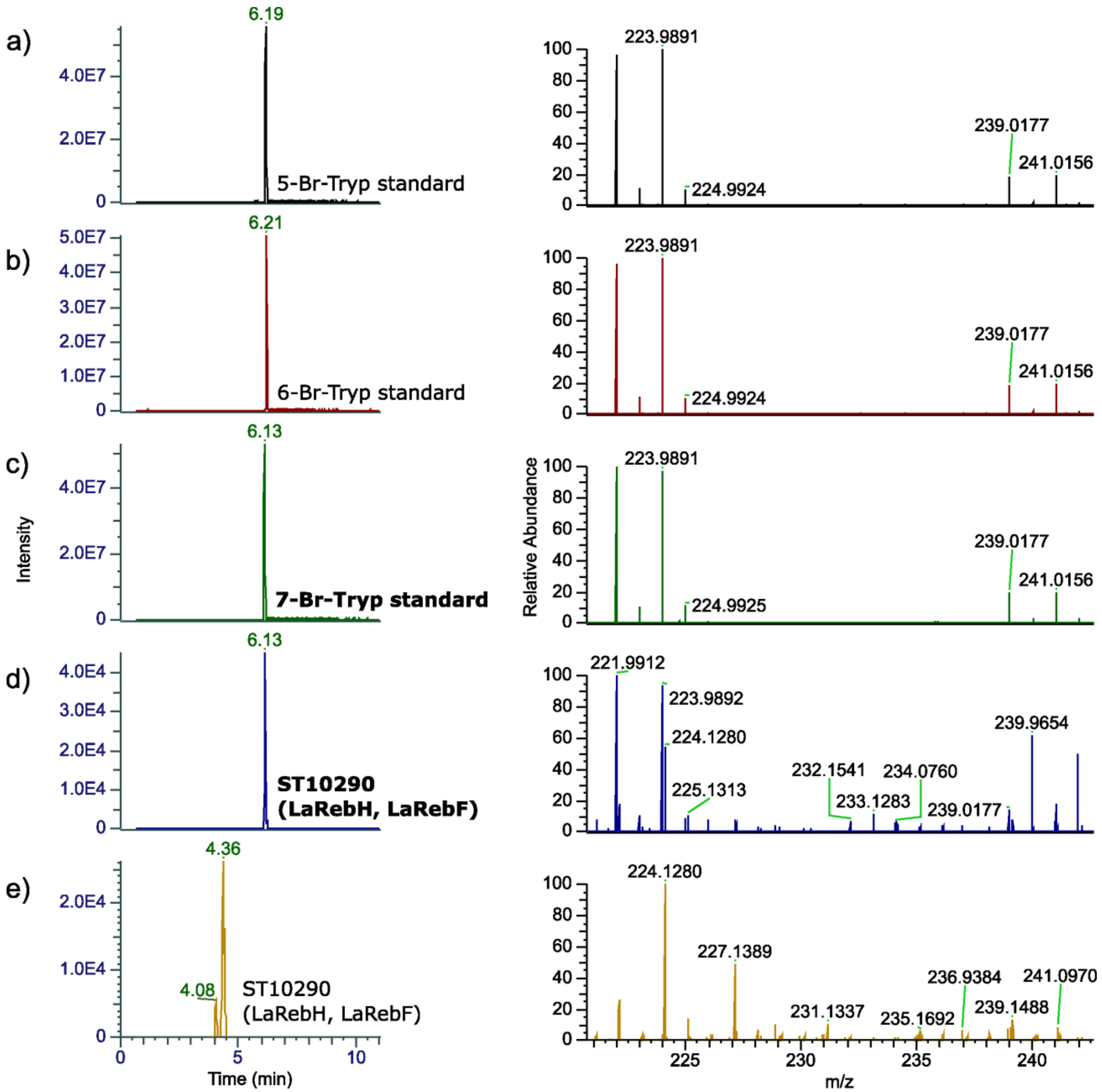

Figure S27. Direct bromination of tryptamine in engineered *S. cerevisiae* strain expressing *LaRebH*. LC-MS extracted ion chromatograms and corresponding mass spectra of the main peak for a) 5-bromotryptamine standard, b) 6-bromotryptamine standard, c) 7-bromotryptamine standard, ST10290 (*LaRebH*, *LaRebF*) fed with 1 mM tryptamine, e) ST10290 (*LaRebH*, *LaRebF*) without tryptamine feeding. . Theoretical m/z of $[M+H]^+$ and $[M+H-NH_3]^+$ adducts with ^{79}Br and ^{81}Br isotopes is 239.0178/221.9913 (most abundant) and 241.0158/223.9832, respectively.

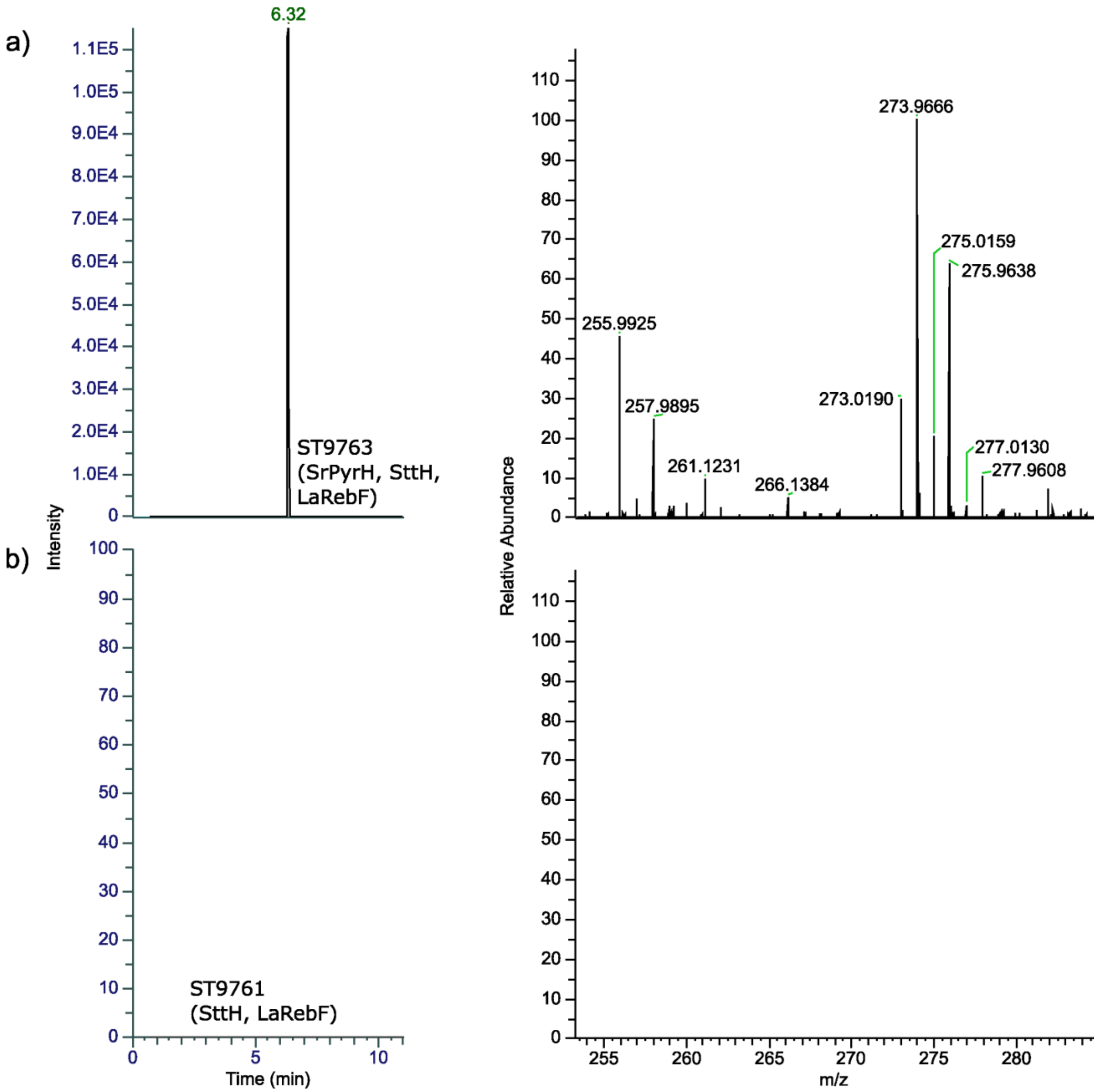

Figure S28. Production of 5,6-dichlorotryptophan in engineered *S. cerevisiae* strains. LC-MS extracted ion chromatograms and corresponding mass spectra of the main peak for a) ST9763 (*SrPyrH*, *SttH*, *LaRebF*), b) ST9761 (6-chlorotryptophan control, *SttH*, *LaRebF*). Theoretical m/z of $[M+H]^+$ and $[M+H-NH_3]^+$ adducts with $^{35}Cl/^{35}Cl$, $^{35}Cl/^{37}Cl$, and $^{37}Cl/^{37}Cl$ isotopes is 273.0192/255.9927 (most abundant), 275.0163/257.9897 and 277.0133/259.9868, respectively. Note the presence of a dichlorinated compound, likely dichlorinated xanthurenic acid, as the main halogenated product, with observed $[M+H]^+$ m/z of 273.9666 ($^{35}Cl/^{35}Cl$), 275.9698 ($^{35}Cl/^{37}Cl$), and 277.9608 ($^{37}Cl/^{37}Cl$).

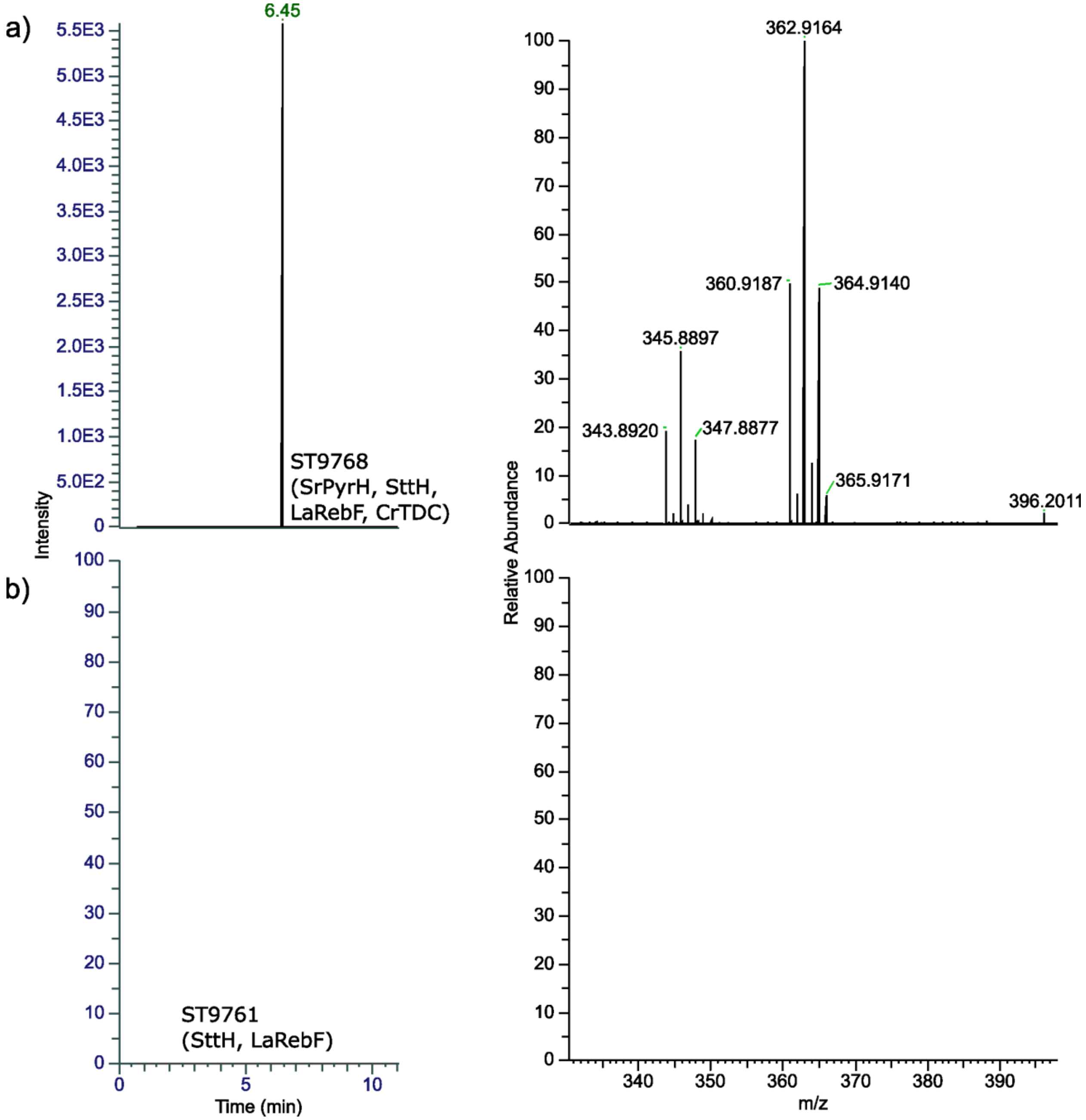

Figure S29. Production of 5,6-dibromotryptophan in engineered *S. cerevisiae* strains. LC-MS extracted ion chromatograms and corresponding mass spectra of the main peak for a) ST9768 (*SrPyrH*, *SttH*, *LaRebF*, *CrTDC*), b) ST9761 (6-chlorotryptophan control, *SttH*, *LaRebF*). Theoretical m/z of $[M+H]^+$ and $[M+H-NH_3]^+$ adducts with $^{79}Br/^{79}Br$, $^{79}Br/^{81}Br$, and $^{81}Br/^{81}Br$ isotopes is 360.9182/343.8916, 362.9161/345.8896 (most abundant), and 364.9141/347.8875, respectively.

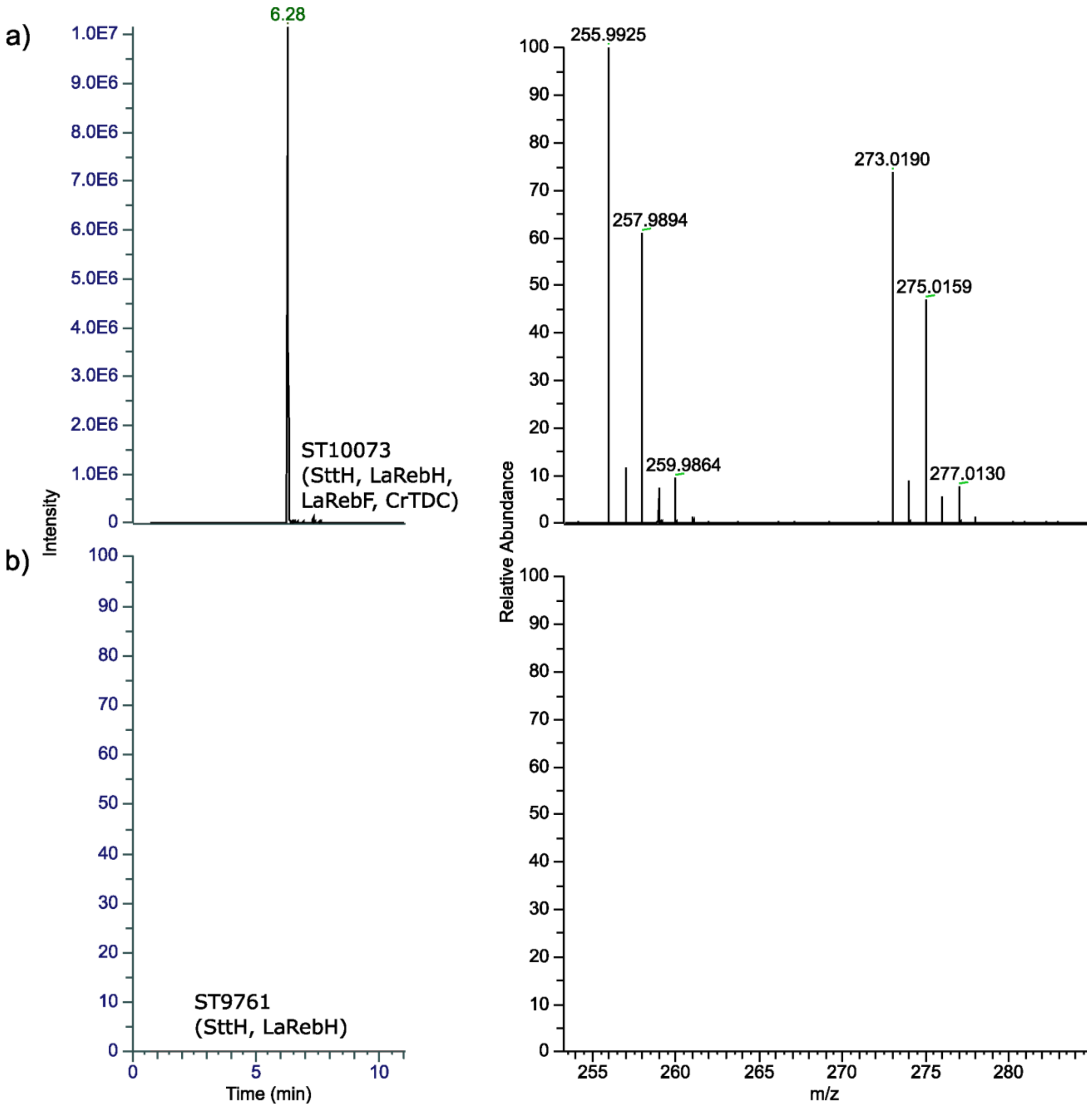

Figure S30. Production of 6,7-dichlorotryptophan in engineered *S. cerevisiae* strains. LC-MS extracted ion chromatograms and corresponding mass spectra of the main peak for a) ST10073 (*SttH*, *LaRebH*, *LaRebF*, *CrTDC*), b) ST9761 (6-chlorotryptophan control, *SttH*, *LaRebF*). Theoretical m/z of $[M+H]^+$ and $[M+H-NH_3]^+$ adducts with $^{35}Cl/^{35}Cl$, $^{35}Cl/^{37}Cl$, and $^{37}Cl/^{37}Cl$ isotopes is 273.0192/255.9927 (most abundant), 275.0163/257.9897 and 277.0133/259.9868, respectively.

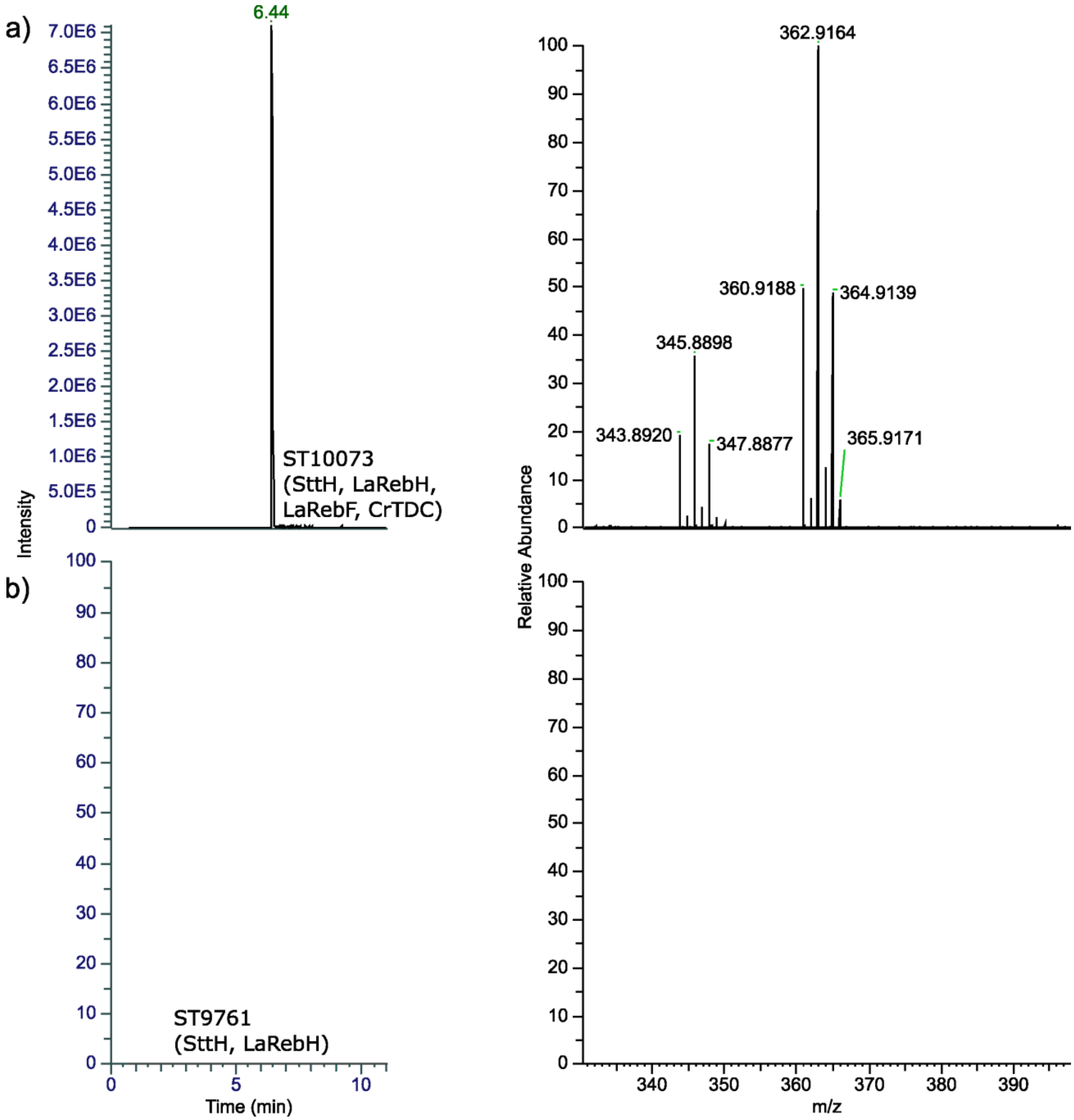

Figure S31. Production of 6,7-dibromotryptophan in engineered *S. cerevisiae* strains. LC-MS extracted ion chromatograms and corresponding mass spectra of the main peak for a) ST10073 (*SttH*, *LaRebH*, *LaRebF*, *CrTDC*), b) ST9761 (6-chlorotryptophan control, *SttH*, *LaRebF*). Theoretical m/z of $[M+H]^+$ and $[M+H-NH_3]^+$ adducts with $^{79}Br/^{79}Br$, $^{79}Br/^{81}Br$, and $^{81}Br/^{81}Br$ isotopes is 360.9182/343.8916, 362.9161/345.8896 (most abundant), and 364.9141/347.8875, respectively.

References:

[1] K.-D. Entian, P. Kötter, in *Methods in Microbiology, Vol. 36* (Eds.: I. Stansfield, M. J. Stark), Academic Press, **2007**, pp. 629-666.

[2] N. Milne, L. R. R. Tramontin, I. Borodina, *FEMS Yeast Res.* **2020**, *20*.

[3] M. M. Jessop-Fabre, T. Jakočiūnas, V. Stovicek, Z. Dai, M. K. Jensen, J. D. Keasling, I. Borodina, *Biotechnol. J.* **2016**, *11*, 1110-1117.

[4] N. Milne, P. Thomsen, N. Mølgaard Knudsen, P. Rubaszka, M. Kristensen, I. Borodina, *Metab. Eng.* **2020**, *60*, 25-36.

Authors' contribution statement

NM: Conceptualization, Methodology, Validation, Investigation, Visualization, Writing - original draft, Writing - Review & Editing. JSS: Investigation, Validation, Visualization, Writing - original draft, Writing - Review & Editing. AMN: Validation, Investigation, Visualization, Writing - original draft. JDK: Investigation, Validation, Visualization, Writing - Review & Editing. DR: Methodology, Validation, Investigation. TW: Methodology, Validation, Investigation. MK: Methodology, Validation, Investigation. IB: Funding acquisition, Project administration, Supervision, Writing - original draft.
